# Lithium-ion conducting self-assembled organic nanowires: optimizing mechanical performance and ionic conductivity through programmable supramolecular interactions†

**DOI:** 10.1039/d5sc00159e

**Published:** 2025-06-03

**Authors:** Vishwakarma Ravikumar Ramlal, Sam Sankar Selvasundarasekar, Akanksha Singh, Jenil Ankola, Rabindranath Lo, Subrata Kundu, Amal Kumar Mandal

**Affiliations:** a Analytical and Environmental Science Division and Centralized Instrument Facility, CSIR-Central Salt and Marine Chemicals Research Institute Bhavnagar Gujarat-364002 India akmandal@csmcri.res.in; b Academy of Scientific and Innovative Research (AcSIR) Ghaziabad-201002 India; c Electrochemical Process Engineering (EPE) Division, CSIR-Central Electrochemical Research Institute (CECRI) Karaikudi Tamil Nadu 630003 India; d Institute of Organic Chemistry and Biochemistry, Czech Academy of Sciences Flemingovo náměstí 542/2 Prague 160 00 Czech Republic

## Abstract

The emergence of wearable devices has led to a greater need for battery materials that are safe, resilient, and exhibit high levels of ionic conductivity. Here, we present a supramolecular design as a useful tactic through fine tuning of the noncovalent interactions to overcome the standard trade-off in solid state Li-ion conductors between ionic conductivity and mechanical resilience. We report solution processable self-assembled organic nanowires (SONs) with varying supramolecular interactions through structural mutation to boost Li-ion conductivity and mechanical integrity. The findings indicate that precise H-bonding plays a crucial role in achieving a maximum Young's modulus (1050.5 ± 38 MPa) and toughness (15 666 ± 423 kJ m^−3^), surpassing the impact of the number of H-bonding sites. The highly structured H-bonded morphology facilitated the creation of binding pockets, enhancing lithiation, in achieving the highest ionic conductivity (3.12 × 10^−4^ S cm^−1^) with a Li-ion transference number of 0.8 at 298 K. The molecular dynamics simulation demonstrates that, among the various interaction sites, the hopping of Li-ions through the axial pathway is favoured over the planar pathway. This study represents a pioneering example illustrating the methodology behind the impact of noncovalent interactions within nanoscale assemblies on the ion conductivity and mechanical characteristics of supramolecular Li-ion conductors.

## Abstract 2

We introduce self-assembled organic nanowires featuring programmable supramolecular interactions as an effective strategy to address the traditional trade-off between mechanical strength and ionic conductivity in solid-state Li-ion conductors.

## Graphical abstract

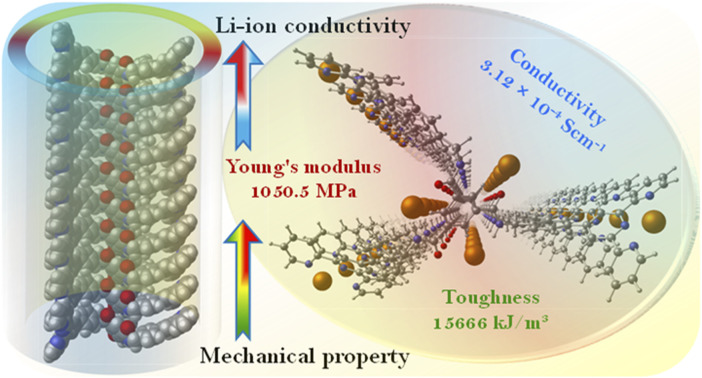

## Introduction

The dominance of smart portable electronics, electric vehicles, grid-scale storage systems, and wearable soft electronics in the current era of ubiquitous energy has significantly heightened the need for safer and more energy-efficient power sources.^1–4^ Due to a combination of high theoretical specific capacity, ultra-negative electrochemical potential, and low density, Li-ion batteries (LIBs) stand out as a prime contender for the next generation of portable energy storage units.^5–8^ Modern LIBs use flammable liquid electrolytes for ion transport, which has led to numerous battery fires and explosions due to their high flammability.^9–11^ Considering the challenges, researchers have focused on developing solid-state electrolytes (SSEs) to meet the performance demands of modern LIBs. SSEs are the battery's core component, and their quality directly affects performance. To meet the practical application, SSEs should have high ionic conductivity, transference number, mechanical stability, and excellent chemical, electrochemical, and thermal stability.^1,11,12^ In consequence, an intensive study to explore the underlying mechanisms and fundamental understandings is highly desirable to overcome the grand challenges of SSEs. Recent research has focussed on employing inorganic ceramics/glass or organic polymer-based materials as SSEs.^13–15^ The unique structural feature of inorganic SSEs favors achieving a wide electrochemical stability window with high ionic conductivity and transference number, although their rigid nature creates high interfacial resistance due to poor contact with the electrode surface. In addition, the poor chemical stability of some inorganic electrolytes unavoidably results in unstable solid electrolyte interphase layers. They are also more brittle, reduce processing performance, and create a challenge to obliterate the pores. Li-dendrites penetrate the electrolyte through these pores and short-circuit the cells under specific conditions.^11,16^ In this regard, the excellent flexibility and easy processability of organic polymers make them promising materials for SSEs,^17–19^ although sluggish ionic conductivity and transference number at room temperature restraint their practical application. According to the Vogel–Tamman–Fulcher (VTF) relationship, increased ionic conductivity in polymer electrolytes is correlated with a lower glass transition temperature (*T*_g_).^20^ Consequently, over the past four decades, research on polymer electrolytes has mostly focused on lowering *T*_g_ to improve ionic conductivity.^21–23^ Tactlessly, lowering the *T*_g_ of a polymer is deleterious to its mechanical strength and could lead to hazards such as short-circuiting *via* an external puncture or dendrite formation.^24,25^ It is, therefore, a challenge to develop polymer electrolytes with strong mechanical properties and good ionic conductivity. To avoid the canonical trade-off between ionic conductivity and the mechanical properties in SSEs, it is crucial to slot in material abundance.

Numerous polymer engineering strategies, such as nanoscale-phase separation, crosslinking with hairy nanoparticles, and the addition of ceramic fillers and framework materials, have been investigated for Li-ion conductors to broaden the accessible material pool.^26–31^ Even though these systems have produced encouraging results, it is still difficult to strike a balance between the necessity for high conductivity and mechanical robustness in a processable platform.^32–34^ For framework materials, the mechanical and ion transport properties vary amongst cells because of their unprocessability and nonuniform particle size.^35,36^ To fill these scientific fissures, self-assembled supramolecular nanowires (SONs) that emerge from the molecular organization of repeating organic units shaped through reversible noncovalent interactions have received considerable attention. Their ability to leverage the properties in the assembled state, easy solution processability, and mechanical and chemical robustness while remaining reversible and responsive make them ideal for exploration as solid Li-ion conductors. Some other features, like flexibility in the design of the purpose-built target molecules by incorporating a new synthetically accessible functionality, give the option to fine-tune the ion conductivity, interface stability, and electrochemical performances.^37–40^ Aside from that, their flexible, lightweight, and adaptable nature with tunable, reversible noncovalent interaction for tweaking mechanical strength makes them an important class of materials for designing efficient SSEs.^41–43^ Despite their significance, developing systematic design strategies for monomers and efficient techniques for producing SONs remains a formidable challenge. Furthermore, obtaining the desired SONs through self-assembly processes is hindered by the complexity of managing numerous noncovalent interactions in a dynamic environment.^44^ Our group recently developed an easy and facile self-assembly approach to preparing supramolecular nanostructures from a cationic guanidinium derivative with three-fold symmetry. The guanidinium derivatives in their zwitterion form self-assembled to form an organic nanosheet with arrays of one-dimensional channels that facilitates the movement of Li-ions, with an ion conductivity of 5.14 × 10^−5^ S cm^−1^ at ambient temperature.^41^ We have systematically changed the directional channel's size and location on the framework using positional isomers to further increase the ion conductivity (3.42 × 10^−4^ S cm^−1^).^38^ A self-assembled organic nanotube produced by imine condensation was recently described by Dichtel *et al.* as a supramolecular Li-ion conductor with an ion conductivity of 3.91 × 10^−5^ S cm^−1^.^45^ In certain instances, to increase conductivity, the nanochannel created *via* the supramolecular self-assembly technique was used to encourage the directional migration of Li-ions while preventing anions from entering *via* size exclusion.^46^ In some circumstances, high charge density has been integrated into self-assembled ordered organic backbones to improve ion transport *via* electrostatic repulsion.^47^ Yet, the contemporary literature lacks a thorough examination of the fundamental principles and mechanisms governing noncovalent interactions in self-assembled supramolecular conductors, which are crucial for enhancing ion conductivity and mechanical strength.

**Fig. 1:**
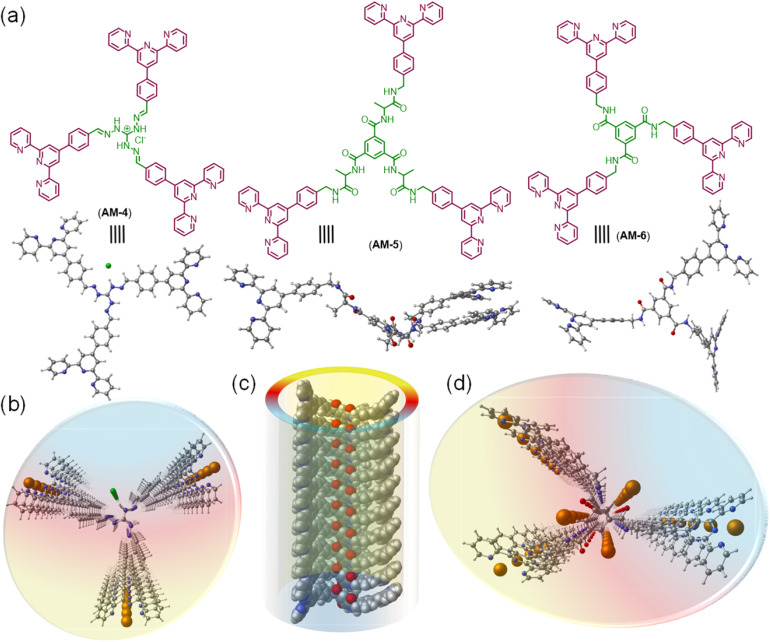
(a) Chemical and computationally optimized structures of *C*_3_-symmetrical molecules AM-4, AM-5, and AM-6, respectively. (b) Schematic representation of the top view of the SONs of AM-4 with Li-ion migration. (c and d) Schematic representation of the SONs of AM-6 with Li-ion migration through possible binding pockets (carbon = grey; nitrogen = blue; oxygen = red; chloride = green; Li = orange).

This work presents a novel class of supramolecular self-assembled organic nanowires (SONs) as solid-state Li-ion conductors that are used to boost mechanical properties, ion-pair dissociation, and ion conductivity. To elucidate the role of the various secondary interactions throughout the self-assembly process, structural mutations like varying numbers of central amide functionalities and guanidium units of three *C*_3_-symmetrical molecules (AM-4, AM-5, and AM-6) have been carried out (Fig. 1). We propose two crucial concepts for the structure's design: (1) the cationic guanidinium unit's incorporation into the structure increases ion-pair dissociation, improving ion conductivity and transference number; (2) the adroit design with varying numbers of H-bonding sites helps to tune the mechanical properties of SONs and well-defined directional channels for ion conduction. Self-assembly in the DMF/water solvent system produced a distinct nanowire morphology for three molecules. Variable temperature UV-vis spectroscopy and other conventional characterization methods are used to determine the thermodynamic parameters and the association constant for this morphological evolution caused by the delicate balancing of various supramolecular interactions. The thin film membranes derived from the dispersion of each type of nanorod offered valuable insights into the influence of non-covalent interactions on the mechanical properties of the nanowires, achieving a maximum Young's modulus of 1050.5 ± 38 MPa and a toughness of 15 666 ± 423 kJ m^−3^. Upon lithiation of these developed SONs, the highest ionic conductivity (3.12 × 10^−4^ S cm^−1^) was attained, accompanied by a Li-ion transference number of 0.8 at 298 K. A comparative analysis of the Li-ion conduction performance of SONs through computational and molecular dynamics (MD) simulations has demonstrated that the well-organized H-bonded structure of SONs promotes the formation of binding pockets, thereby improving lithiation. Additionally, the presence of a long-range ordered directional channel supports the efficient hopping of Li-ions along the axial pathway. Presumably, this is the first demonstration to articulate the design strategy of the role of non-covalent interactions on nanoscale assemblies in influencing the mechanical properties and ion conductivity in supramolecular Li-ion conductors.

## Results and discussion

The molecular design of three tripodal molecules (AM-4, AM-5, AM-6) aims to rationalize the mechanical properties, ion-pair dissociation, and ion conductivity of SONs through a delicate balance of several noncovalent interactions like hydrophobic effects, electrostatic interactions, H-bonding and π–π stacking during the self-assembly process (Fig. 1). The basic design strategy of AM-4 involves a central *C*_3_-symmetric positively charged guanidium unit appended with peripheral terpyridine units. We expect that during the self-assembly process, the central guanidium unit will favor the formation of directional H-bonding, and following its application, the positive charge will cause ion pair dissociation. The role of the terpyridine moiety is to form π–π stacking interactions, while at the same time creating a hydrophobic pocket to shield the core H-bonding during the self-assembly process. The design strategy of AM-5 and AM-6 is based on the well-studied *C*_3_-symmetrical benzene-1,3,5-tricarboxamide core due to its ability to form triple H-bonds with exterior terpyridine units to direct the self-assembly process. For AM-5, the core unit was extended with an alanine spacer to increase the number of H-bonding sites. We anticipate that the terpyridine moiety and central benzene unit can form two different hydrophobic pockets through π–π stacking during the self-assembly process to guard the core H-bonding as well as alter the Li-ion dynamics during its application as SSEs. A divergent synthetic approach was used for the synthesis of three tripodal molecules. A common synthetic intermediate was synthesized in four high-yielding steps followed by standard amide coupling with the corresponding benzene-1,3,5-tricarboxamide derivatives to yield AM-5 and AM-6 (Fig. S1†). Three high-yielding steps followed by condensation of corresponding guanidium derivatives are necessary to synthesize AM-4 (Fig. S1†). The experimental and characterization details of all the synthesized compounds are shown in Fig. S2–S11†.

**Fig. 2:**
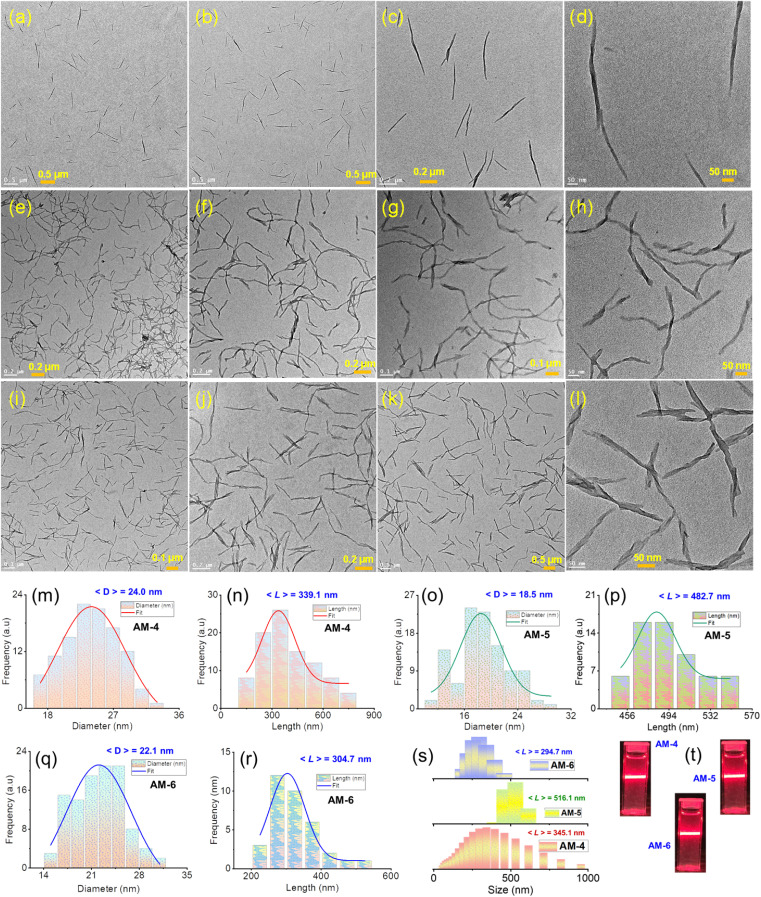
TEM images of the SON morphology of (a–d) AM-4, (e–h) AM-5, and (i–l) AM-6 were obtained in a 1 : 1 v/v DMF/water solvent system. Corresponding statistical size distribution in terms of diameter and length for (m and n) AM-4, (o and p) AM-5, and (q and r) AM-6, respectively. (s) DLS size distribution profile of the as-prepared SON morphology. (t) Corresponding Tyndall scattering of the dispersed SON morphology.

To investigate the self-assembled morphology, a solution of all three molecules (AM-4, AM-5, and AM-6) in DMF (500 μL, 1.0 mg mL^−1^) was exposed to 500 μL of deionized water at 298 K. The solution was mixed thoroughly and left undisturbed for about 10 min to complete the automated self-assembly process. An aliquot of these suspensions was drop-cast on the transmission electron microscopy (TEM) grid and dried at room temperature. The visualized images display the well-dispersed consistent formation of nanowire morphology for all three molecules (Fig. 2a–l). Statistical analysis of the respective images revealed the average diameter (24.0 nm, 18.5 nm, and 22.1 nm) and average length (339.1 nm, 482.7 nm, and 304.7 nm) of the formed nanowire morphology for AM-4, AM-5, and AM-6, respectively (Fig. 2m–r). Dynamic light scattering (DLS) measurements disclose the self-assembled nanowire dispersion of AM-4 having a wide size distribution with an average size of 345.1 nm (Fig. 2s). Likewise, for the as-prepared nanowire dispersion of AM-5 and AM-6, a small size distribution with an average size of 516.1 nm and 294.7 nm was perceived (Fig. 2s). The distinct Tyndall scattering of laser light provided visible confirmation of the colloidal stability of each of these nanostructures (Fig. 2t).

**Fig. 3:**
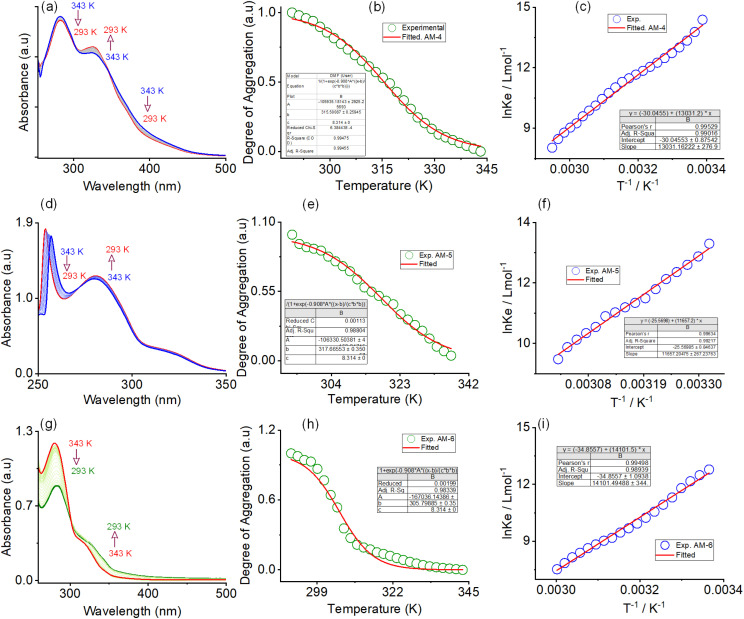
Variable-temperature UV-vis spectra of (a) AM-4 and (d) AM-5 and (g) AM-6 in DMF/water (1 : 1, v/v). Corresponding plot of the temperature-dependent degree of aggregation and corresponding isodesmic fit for (b) AM-4, (e) AM-5, and (h) AM-6, respectively. Corresponding van't Hoff plot for (c) AM-4, (f) AM-5, and (i) AM-6, respectively.

The formation of nanowire morphology through the self-assembly process was systematically monitored by UV-vis and fluorescence spectroscopy in a DMF/water solvent system. A gradual decrease in the absorption maxima of all three molecules was observed upon systematically increasing the water percentage in DMF solutions (Fig. S12–S14†). The corresponding trend of spectral changes signposts the transformation of the monomeric state into a self-assembled state with increasing water content. For AM-4, a steady increase in the emission intensity with 49% enhancement at 368 nm was observed with a systematic increase in the water content in DMF (Fig. S12†). The trend of spectral changes suggests that the presence of water favors the self-assembly process and restricts the internal rotation of the –C=N bond responsible for the observed emission enhancement. In contrast, with AM-5 and AM-6, the self-assembly process resulted in a consistent drop in emission intensity as water concentrations increased (Fig. S13 and S14†). The thermodynamic parameters of the self-assembly process were evaluated using temperature-dependent UV-vis spectroscopy upon cooling the solution (1 : 1 DMF/water v/v) from 343 to 293 K at a cooling rate of 2 K per 5 min (Fig. 3 and S15–S17†). Upon systematic cooling, AM-4 showed a steady drop in absorbance at 282 and 394 nm and a rise in the absorption maxima at 327 nm. The appearance of two distinct isosbestic points at 302 and 345 nm in the spectrum change trend suggests that the cooling process converts the monomeric state into an aggregate state (Fig. 3a). Likewise, for AM-5, an isosbestic point at 272 nm was observed with synchronized growth and bleach of absorption maxima at 282 nm and 263 nm (Fig. 3d). The solution absorption for AM-6 increased at 340 nm, decreased at 281 nm, and reached an isosbestic point at 301 nm upon cooling (Fig. 3g). For all three self-assembly processes, the distinct features of the absorbance change and the degree of aggregation (*α*_agg_) as a function of temperature point to an isodesmic aggregation process (Fig. 3b, e and h).^38,42,43,48^ The aggregation association constants (*K*_iso_) at various temperatures were determined by fitting the degree of aggregation with the thermal isodesmic aggregation model (Fig. S15–S17†). The aggregation association constants (*K*_iso_) of 9.56 × 10^5^ M^−1^, 7.95 × 10^5^ M^−1^, and 1.22 × 10^6^ M^−1^ were estimated at 297 K for the self-assembly process of AM-4, AM-5, and AM-6, respectively. The thermodynamic parameters for the three self-assembly processes were obtained from the van't Hoff plot of the reciprocal temperature and the logarithm of (*K*_iso_) (Fig. 3c, f and i). For AM-4, the estimated values of standard enthalpy (Δ*H*^0^) and entropy (Δ*S*^0^) were −108.34 kJ mol^−1^ and −249.75 J mol^−1^ K^−1^, respectively. Similarly, standard enthalpies of −96.91 kJ mol^−1^, −117.23 kJ mol^−1^, and entropies of −212.5 J mol^−1^ K^−1^, −289.7 J mol^−1^ K^−1^ were estimated for the self-assembly process of AM-5 and AM-6, respectively. Each of the three self-assembly processes is enthalpy-driven, as indicated by the negative values of Δ*H*^0^ and Δ*S*^0^. An additional indication of the enthalpy and entropy value pattern is that AM-6 entangled the greatest number of molecules in the aggregation process, with AM-4 and AM-5 following suit.^38,42^ The trend of aggregation association constant suggests that AM-6 forms the most stable self-assembled structure, followed by AM-4 and AM-5, respectively.

**Fig. 4:**
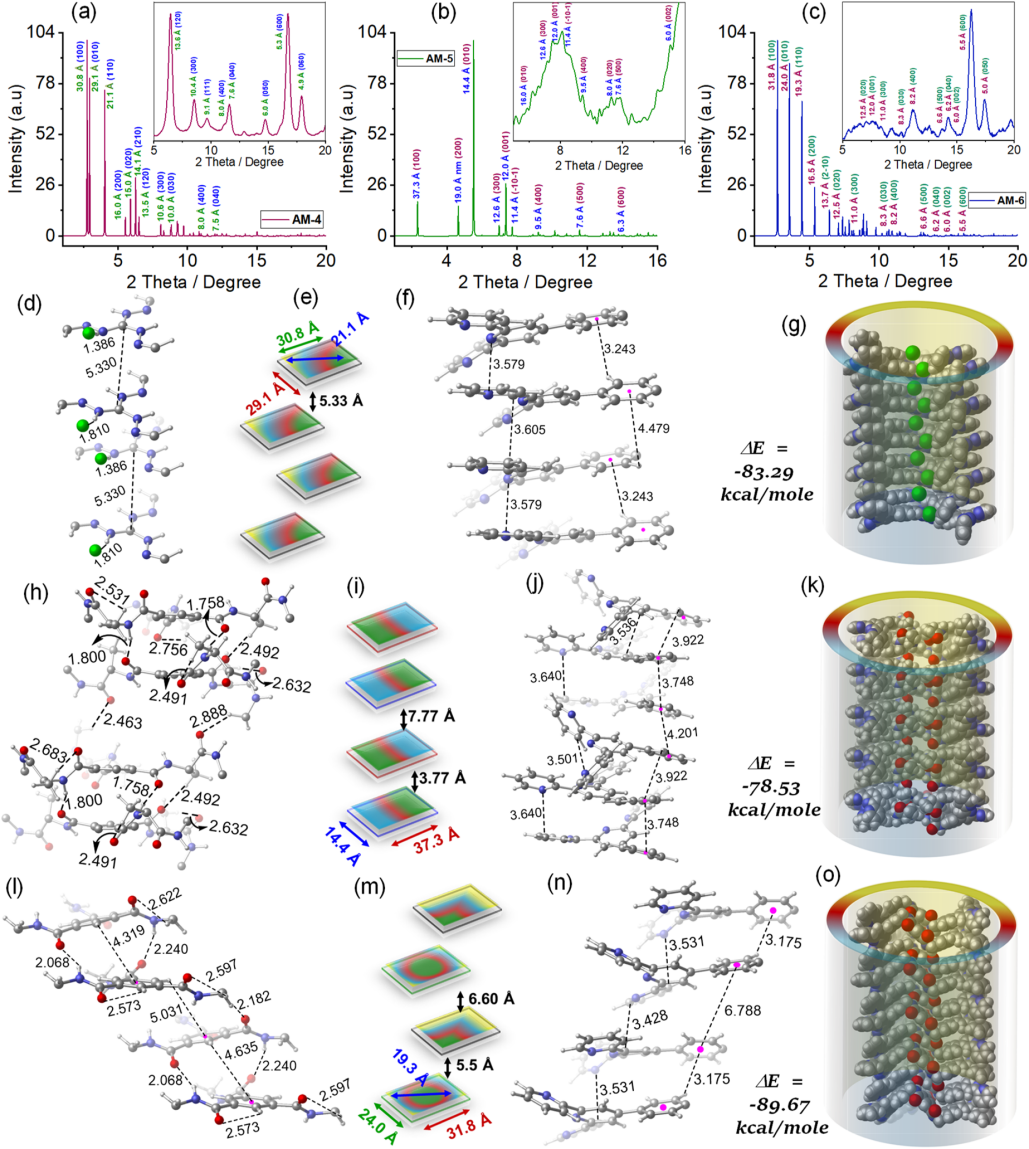
Simulated XRD profile for the SONs of (a) AM-4 (inset experimental XRD profile), (b) AM-5 (inset experimental XRD profile), and (c) AM-6 (inset experimental XRD profile), respectively. Schematic representation and optimized geometries with possible noncovalent interactions for (d–g) SONs of AM-4, (h–k) SONs of AM-5 and, (l–o) SONs of AM-6, respectively. The unit of bond distances is in Å [C: grey, N: blue, H: white, O: red, Cl: green].

To envisage the molecular interaction responsible for this self-assembly process, we have optimized all geometries under periodic conditions using VASP software and the theoretical X-ray diffraction (XRD) is simulated by the VESTA Crystallographic Software (Fig. 4). Simulated XRD spectra from the optimized structure of AM-4 revealed peaks with the *d*-spacing values of 30.8 Å (100), 29.1 Å (010), and 21.1 Å (110), corresponding to the length, width and diagonal length of the molecule (Fig. 4a and e). The calculated *d*-spacing values of 30.8, 16.0, 10.6, 8.0, and 6.4 Å, respectively, designating diffraction from (001), (002), (003), (004), and (005) planes, indicate the ordered lamellar packing (Fig. 4a). The experimentally obtained well-defined sharp reflection peaks at 2*θ* = 8.52°, *d* = 10.4 Å (300), 2*θ* = 11.12°, *d* = 8.0 Å (400), 2*θ* = 11.61°, *d* = 7.6 Å (040), 2*θ* = 14.73°, *d* = 6.0 Å (050), 2*θ* = 16.73°, *d* = 5.3 Å (600) and, 2*θ* = 17.92°, *d* = 4.9 Å (060), respectively further suggest the ordered lamellar packing (inset Fig. 4a). To gain a deeper understanding of the supramolecular interactions, a detailed analysis of the packing and hydrogen bonding interaction of AM-4 was conducted (Fig. S18†). The tripodal molecule AM-4 is oriented in double layers in a zigzag pattern with an interlayer distance of 5.3 Å (Fig. 4e and g). The double-layer is held together by C–H⋯π (3.2 and 4.4 Å) and π⋯π (3.5 and 3.6 Å) stacking interactions between the peripheral phenyl terpyridine moieties (Fig. 4f). The central guanidium unit displayed a short N–H⋯Cl (1.4 and 1.8 Å) H-bonding with a –C–C– distance of 5.3 Å (Fig. 4d). Interestingly, the experimentally obtained *d*-spacing value of 5.3 Å, corresponding to the characteristic intense reflection peak at 2*θ* = 16.73° associated with diffraction of the (600) plane, matches well with the intermolecular –C–C– distance. Fig. 4g depicts the space-fill model of the one-dimensional (1D) growth of AM-4 to a *syn*-oriented catemer with stabilization energy Δ*E* = −83.29 kcal mole^−1^ through a delicate balance of various noncovalent interactions through the self-assembly process. In the optimized asymmetric unit of AM-5, two of the three tripodal arms interconnect through a series of noncovalent interactions (Fig. S19†). A close analysis shows that two –C–H⋯O H-bonding interactions with distance 2.6 and 2.7 Å along with π⋯π (3.5–3.9 Å) stacking interactions are responsible for this intramolecular stacking of two arms (Fig. 4h and j). Following that, it forms an *anti*-oriented intermolecular double-layered motif that is bound together by two potent H-bonding interactions: –C–H⋯O (2.5 Å) and –N–H⋯O (1.8 and 1.7 Å). Additionally, π⋯π stacking interaction between the central benzene ring (3.7 Å) and peripheral phenyl terpyridine moiety (3.6–3.7 Å) was noted (Fig. 4j). The double layer motif progresses in a one-dimensional fashion to form an *anti*-oriented catemer, with a stabilization energy of −78.53 kcal mole^−1^ (Fig. 4k). This transformation is aided by intermolecular interactions such as C–H⋯O (2.4 and 2.8 Å) H-bonding and π–π (4.2 Å) stacking interactions. The molecule's length and width are represented by the peaks with the *d*-spacing values of 37.3 Å (100), and 14.4 Å (010) in the optimized structure of AM-5's simulated XRD spectra (Fig. 4b and i). The ordered lamellar packing is indicated by the computed *d*-spacing values of 37.3, 19.0, 12.6, 9.5, 7.6, and 6.3 Å, respectively, denoting diffraction from the (001), (002), (003), (004), (005) and (006) planes (Fig. 4b).

Further evidence for the ordered packing is provided by the experimentally produced well-defined sharp reflection peaks at 2*θ* = 5.51°, *d* = 14.6 Å (010), 2*θ* = 6.97°, *d* = 12.6 Å (300), 2*θ* = 7.36°, *d* = 12.0 Å (001), 2*θ* = 9.30°, *d* = 9.5 Å (400), 2*θ* = 11.05°, *d* = 8.0 Å (020), 2*θ* = 11.63°, *d* = 7.6 Å (500) and, 2*θ* = 14.75°, *d* = 6.0 Å (002), respectively (inset Fig. 4b). The experimental *d*-spacing values of 3.7 and 7.7 Å are particularly significant as they match the distinct diffraction patterns of (003) and (020) planes. These values also closely correlate with the intermolecular centroid distance between core benzene rings. The optimized geometry of AM-6 exhibits a configuration reminiscent of a tripodal propeller shape (Fig. S20†). In the elongated catemer structure, the repeating units are organized in a zigzag fashion, with each alternating repeating unit aligned in a *syn*-orientation. The strength of the double-layered assembly is maintained by the presence of –N–H⋯O (2.0 and 2.2 Å) and –C–H⋯π (4.3 Å) interactions at the core, along with –C–H⋯π (3.1 Å) and π⋯π (3.5 Å) stacking interactions surrounding the phenyl terpyridine moiety at the periphery (Fig. 4l and n). The progression of the double layer motif follows a one-dimensional zigzag trajectory, resulting in the formation of an elongated catemer with a stabilization energy of −89.67 kcal mole^−1^, facilitated by –C–H⋯O (2.1 Å) and π–π (3.4 Å) stacking interactions (Fig. 4o). The XRD patterns simulated based on the optimized geometry exhibited distinct peaks at *d*-spacing values of 31.8 Å (100), 24.0 Å (010), and 19.3 Å (110), indicative of the molecule's length, width, and diagonal distance (Fig. 4c and n). The *d*-spacing measurements of 31.8, 16.5, 10.6, 8.2, 6.6, and 5.5 Å correspond to diffraction from (001), (002), (003), (004), (005), and (006) planes, demonstrating the organized lamellar arrangement (Fig. 4c). The well-defined and precise reflection peaks observed in the experimental results at 2*θ* = 8.28°, *d* = 10.6 Å (300), 2*θ* = 11.05°, *d* = 8.2 Å (400), 2*θ* = 13.40°, *d* = 6.6 Å (500) and, 2*θ* = 16.20°, *d* = 5.5 Å (600), indicate a highly organized arrangement of layers (inset Fig. 4c). Notably, the experimental *d*-spacing values of 5.5 and 6.6 Å align with the characteristic diffraction patterns of (600) and (500) planes, respectively, which also closely correspond with the intermolecular centroid distance between core benzene rings.

X-ray photoelectron spectroscopy (XPS) analyses were conducted to gain insight into the composition and chemical environment of the SONs (Fig. S21†). The XPS survey spectra corroborate the presence of constitutive elements such as carbon, nitrogen, oxygen, and chlorine within the corresponding SONs. Calculating nitrogen-to-oxygen abundance ratios for AM-5 and AM-6 involved adjusting the peak area by photoionization cross-section, resulting in 15.17 : 6.93 and 14.35 : 4.10, respectively. The experimental value demonstrated a satisfactory level of agreement with the estimated value derived from the molecular structures of AM-5 and AM-6, taking into consideration an associated error margin of 10%. Deconvolution of the high-resolution C 1s spectrum revealed three distinct Gaussian curves, each accounting for the presence of –C–C–, –C–N–, and –C–O– components, respectively. Through deconvolution, the N 1s spectrum was resolved into two Gaussian curves with the binding energy of 399.0 eV and 405.5 eV, each indicative of the respective contributions from –N_(terpy)_ and –N_(amide)_ moieties. Thermogravimetric analysis (TGA) was employed to analyse the solvent interactions and thermal stability of SONs. Across all cases, a two-stage weight loss process was evident in the TGA profile (Fig. S22†). The expulsion of H-bonded solvent molecules within AM-4 resulted in an initial weight loss of approximately 5.0%. The subsequent reduction in weight as temperature increases can be attributed to the decomposition of AM-4 (27.5%). Weight losses of approximately 8.0% and 4.9% were noted in relation to the H-bonded solvent molecules, followed by weight losses of 50.0% and 64.0% corresponding to the decomposition of AM-5 and AM-6 as temperature increased.

**Fig. 5:**
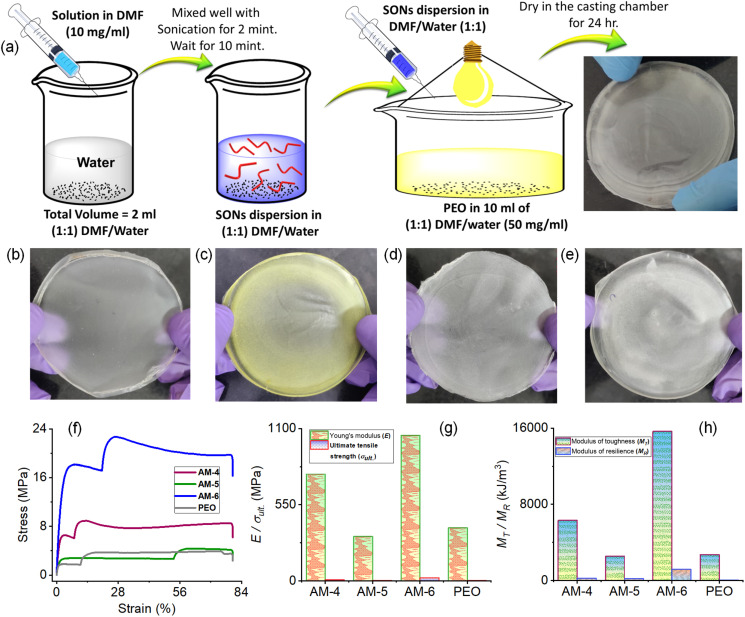
(a) Schematics of the preparation methods of films from the corresponding SONs. Photographs of the corresponding films for (b) PEO, (c) AM-4, (d) AM-5, and (e) AM-6, respectively. (f) Stress–strain diagram from mechanical testing of the corresponding films. (g) A comparison plot of Young's modulus (*E*) and ultimate tensile strength (*σ*_ult._) for the corresponding films. (*h*) Comparison plot of modulus of toughness (*M*_T_) and modulus of resilience (*M*_R_) for the corresponding films.

Composite films of SON assemblies were prepared to explore the effect of various non-covalent interactions on the mechanical properties. Details of the preparation methods of composite films with schematics are shown in Fig. 5a. First, a solution of all three molecules (AM-4, AM-5, and AM-6) in DMF (10 mg mL^−1^) was exposed to 1 mL of deionized water. The solution was mixed thoroughly and left undisturbed for about 10 minutes to complete the automated self-assembly process. This SON dispersion was added to a stirred solution of 10 mL of 1 : 1 DMF/water, and 50 mg mL^−1^ polyethylene oxide (PEO). The homogeneous viscous solution was cast on a Petri dish and dried in the casting chamber for 24 h to obtain composite films. Fig. 5b–e display the photograph and Fig. S30 and 31† reveal the SEM and XRD pattern of the corresponding films. The mechanical properties of the composite films (AM-4, AM-5, and AM-6) were determined using uniaxial tensile stress–strain tests and compared with those of the PEO film. A typical uniaxial tensile stress–strain profile for ductile materials was displayed by all of the composite films (Fig. 5f).^49^ In the initial phase, they demonstrate linear elastic characteristics, where stress and strain maintain a linear relationship. As the strain escalates, the deformation becomes irreversible, resulting in a shift towards non-linear plastic behavior. Table S1† compiles the values obtained from the tensile tests. The elastic segment of the stress–strain curve is defined by parameters such as Young's modulus (*E*) and the upper yield point, while the plastic segment is characterized by ultimate tensile strength (*σ*_ult._) and elongation at break (*ε*_f_).

The composite film derived from the SON assembly of AM-6 revealed the highest (*E*) of 1050.5 ± 38 MPa, surpassing AM-4 (770.8 ± 32 MPa) and AM-5 (320.3 ± 23 MPa) by factors of 1.5 and 3.3, respectively (Fig. 5g). The trend for *σ*_ult._ is similar to that of *E*, with the highest value of 22.7 ± 0.12 MPa observed for AM-6 (Fig. 5g). The composite films derived from the SON assemblies exhibit a significant capacity for energy absorption per unit volume, as demonstrated by their moduli of toughness (*M*_T_). Toughness serves as a critical parameter for assessing the mechanical characteristics of SSEs, as it quantifies the energy absorbed during deformation while encompassing both strength and extensibility. The analysis of the curve reveals that energy absorption is primarily concentrated in the region of plastic deformation, where the modulus of resilience (*M*_R_) achieves its maximum level of 8% of *M*_T_. The trend observed for *M*_T_ aligns with those of *σ*_ult._ and *E*, revealing a peak value of 15 666 ± 423 kJ m^−3^ for AM-6. This value is 2.5 and 6.1 times greater than the values recorded for AM-4 (6321 ± 324 kJ m^−3^) and AM-5 (2554 ± 183 kJ m^−3^), respectively (Fig. 5h). The results strongly suggest that various non-covalent interactions like H-bonding, C–H⋯π and π–π stacking interactions strongly affect the mechanical properties of the SON assembly. The results also reveal that well-ordered directional H-bonding in the SON assembly is important in comparison with the number of H-bonding sites to enhance the mechanical properties. The SON assembly of AM-6 is associated with three –N–H⋯O bonding and two-π–π stacking interaction sites like the central phenyl ring and peripheral terpyridine moiety, whereas the SON assembly of AM-4 is linked with central –N–H⋯Cl and π–π stacking interactions of the terpyridine unit. As a result, the SON assembly of AM-6 displays superior mechanical properties in comparison with AM-4. In contrast, although the SON assembly of AM-5 is associated with six–N–H⋯O bonding and two-π–π stacking interaction sites, the flexible nature of the side arm is the main constraint to form the highly ordered structure. The observed trend in the mechanical properties aligns with the sequence of aggregation association constants and stabilization energies obtained through both experimental and computational methods. The observed mechanical properties are comparable to those of several supramolecular polymers,^50,51^ and, in some cases, surpass those of synthetic polymers and actin filaments.^52,53^

**Fig. 6:**
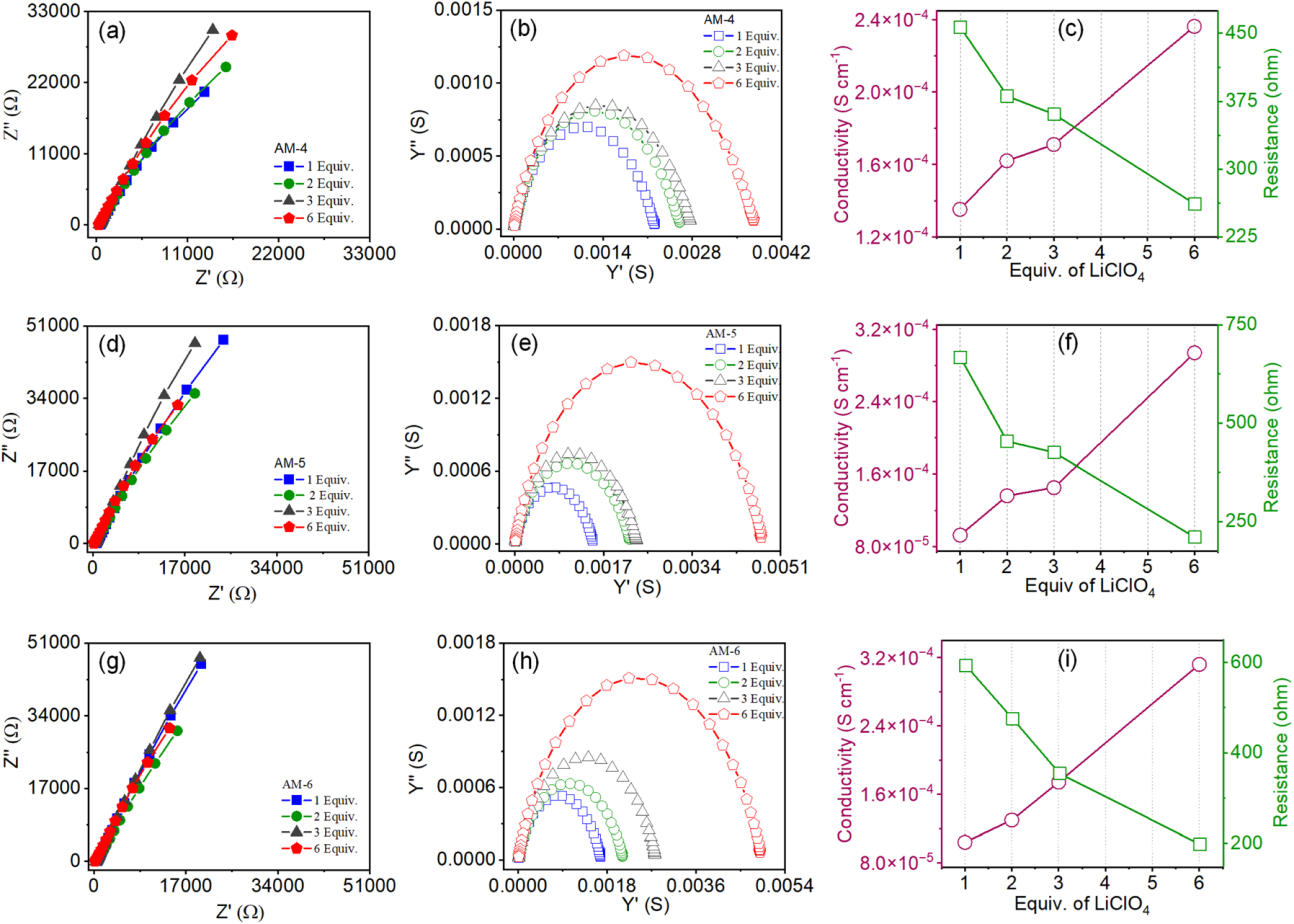
Nyquist plots of SONs doped with varying equivalents of LiClO_4_ for (a) AM-4, (d) AM-5, and (g) AM-6, respectively. Corresponding admittance plots for SONs of (b) AM-4, (e) AM-5, and (h) AM-6, respectively. Corresponding plot of resistance and conductivity data with varying equivalents of LiClO_4_ for SONs of (c) AM-4, (f) AM-5, and (i) AM-6, respectively.

The excellent mechanical properties with the well-defined nanostructure of SONs enthused us to study the role of various noncovalent interactions in Li-ion conductivity properties. To do so, SONs of AM-4, AM-5, and AM-6 were prepared in the presence of varying concentrations of LiClO_4_. The microscopic study indicated that the morphology remained intact after Li-ion incorporation for both cases. The Li-ion conductivity was measured through our recently established method and is mentioned in the ESI.†^38,41,42^ For each SON morphology, we have prepared four sets of samples with varying equivalents of LiClO_4_. The Nyquist plots obtained from ac impedance measurements, with a clear Warburg line, indicate a diffusion-controlled process mainly governed by mass transfer for each set of samples (Fig. 6a, d and g). Hence, the charge transfer is minimal.^41^ The corresponding admittance plots revealed the same (Fig. 6b, e and h). As the admittance plot is inversely proportional to the *Z* value, the kinetics resembles the predominantly produced characteristic feature with a distorted semicircle. Fig. 6 displays the corresponding plots of ion conductivity and resistance with varying equivalents of LiClO_4_, and the corresponding data are shown in Tables S2–S4.† For all sets of samples, a systematic upsurge of ion conductivity was observed with increasing LiClO_4_ concentration under the same conditions. In the case of SONs of AM-4, a 1.8-fold increase in the conductivity value from 1.35 × 10^−4^ S cm^−1^ to 2.36 × 10^−4^ S cm^−1^ was observed with increasing the LiClO_4_ concentration from 1 to 6 equivalents at room temperature (Fig. 6c).

Noticeably, a three-fold boost of ion conductivity with a maximum value of 2.94 × 10^−4^ S cm^−1^ was witnessed in the presence of 6 equivalents of LiClO_4_ for SONs of AM-5 under identical conditions (Fig. 6f). SONs of AM-6 display the highest conductivity of 3.12 × 10^−4^ S cm^−1^ under identical conditions, a 3-fold enhancement in comparison with one equivalent of LiClO_4_ (Fig. 6i). Excitingly, the observed highest ion conductivity value is 9.3-fold higher than that of our recently reported zwitterionic organic nanosheets and comparable with the other positional isomers.^38,41^ The observed ion conductivity value is much higher than those of the previously reported self-assembled organic nanotubes and was found to be comparable with those of the best reported covalent and metal–organic frameworks.^19,32,36,45^ A comparison chart of the best-reported materials with lithium-ion conductivity values is presented in Table S5.† The Bruce–Vincent method was used for the calculation of the Li-ion transference number (*t*_Li^+^_). The details of the measurement with relevant plots and data are shown in Fig. S23–S25 and Table S6.† SONs of AM-4 and AM-6 display Li-ion transference numbers of 0.8 at room temperature. Notably, a decrease in transference numbers of 0.4 was observed for SONs of AM-5 under identical conditions. The higher transference number emphasizes the presence of a central cationic guanidium unit for AM-4 and the structural superiority of AM-6 in comparison with the SONs of AM-5. The measured transference numbers are comparable with some previously reported porous crystalline and supramolecular polymer ion conductors.^19,38,54,55^ A chronoamperometry study was carried out using a Ti/SONs/Li asymmetric cell to examine the electrochemical stability of the SONs. Corresponding potentiostatic spectra for the SONs of AM-4, AM-5, and AM-6 are shown in Fig. S26–S28.† The spectra for all cases demonstrate improved stability over a 10-hour period with little curve degradation. In an asymmetric cell setup, though the charge transfer process of Li/Li^+^ is highly restricted, the stability of the SONs under potentiostatic conditions is ensured, which is well in accordance with the observed steep curves.

**Fig. 7:**
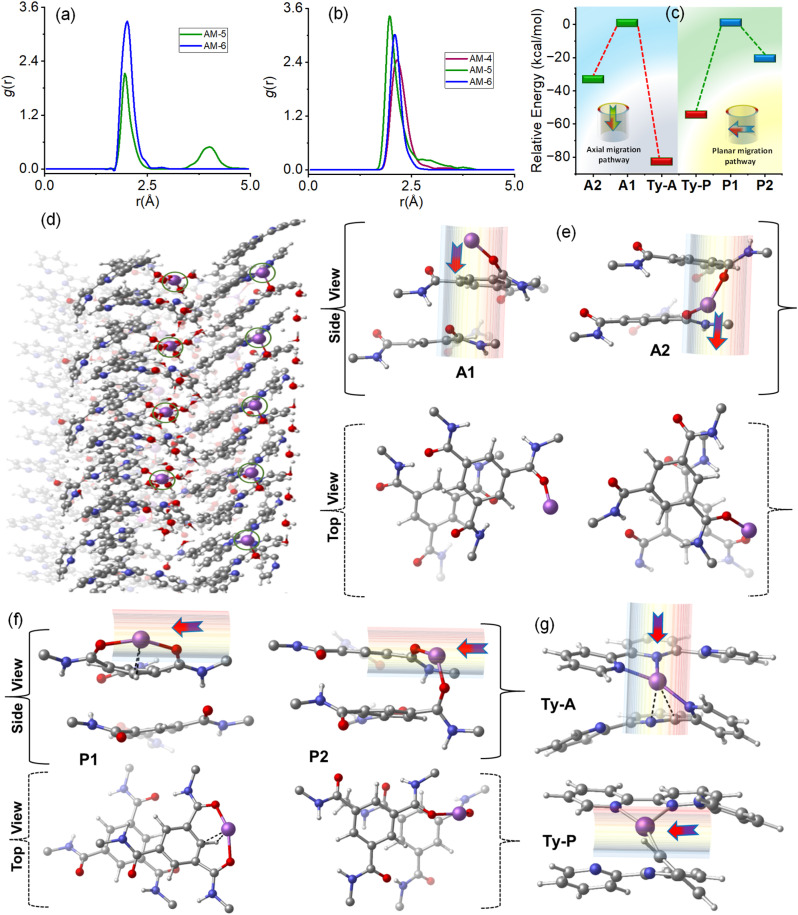
Radial distribution functions of the SONs for the interaction of (a) Li-ions and O atoms of the amide group and (b) Li-ions and N atoms of the terpyridine unit. (c) The plot of the relative energy of the optimized geometries of the different orientations for SONs of AM-6 displays the Li-ion migration in axial and planar pathways. (d) The snapshot is taken from MD simulation for SONs of AM-6 with Li-ion and water molecules at five ps. The Li-ion (in violet color) is shown in a ring for better visibility. The optimized geometries display the Li-ion migration along with amide functionality (e) in axial pathways and (f) in planar pathways, respectively. The arrow shows the direction of Li-ion migration. (g) The optimized geometries display the Li-ion migration along with the terpyridine functionality in axial and planar pathways, respectively [C: grey, N: blue, H: white, Li: violet, O: red].

Molecular dynamics (MD) simulations of the respective SONs were performed to get a deep understanding of the trend of ion conductivity and the Li-ion transference number (Fig. S29†). In a periodic MD-simulation, the stacked geometry confined the Li-ions and water molecules. We consider a dimeric system, where each simulation box includes fifty water molecules and five Li-ions. During the simulation time, Li-ions are solvated by water molecules. Such solvated Li-ions interact mainly with the amide carbonyl oxygen (O) and terpyridine nitrogen (N) of the SONs (Fig. S29†). The radial distribution functions involving Li-ions, amide carbonyl oxygen (O), and terpyridine nitrogen (N) obtained from MD simulations are compared for the respective SONs. In the case of AM-5, two peaks were observed for the interaction between Li-ions and amide oxygen as this contains two types of amide groups (Fig. 7a). In comparison, AM-6 displays one peak, and no such peak is observed for AM-4 (Fig. 7a). The peak height of the plots also suggests that SONs of AM-6 form a stronger interaction with Li-ions in the amide oxygen sites than SONs of AM-5 (Fig. 7a). Fig. 7b displays the corresponding plots of radial distribution function for the interactions of Li-ions and terpyridine nitrogen (N) sites of the respective SONs. The trend of the peak height suggests that SONs of AM-5 display the strongest interaction as the Li-ions interact simultaneously with two terpyridine units of the intramolecular stacked arms. Meanwhile, the cation–cation repulsion of the central guanidinium cation and Li-ion are responsible for the lowest peak height for SONs of AM-4. The Li-ion migration is investigated for SONs of AM-6 through two types of anisotropic pathways: parallel and perpendicular to the axially stacked pores, referred to as axial and planar pathways, respectively. The calculated results show that Li-ion migration through the amide group in the axial path leads to two optimized geometries, A1 and A2 (Fig. 7e). In state A1, the Li-ion interacts with a single oxygen atom of the amide group, whereas in A2, the Li-ion sandwiched between two amide groups interacts simultaneously with two oxygen atoms. The relative energy of the A2 geometry (−32.2 kcal mol^−1^) is much lower than that of the A1 state (Fig. 7c). Meanwhile, the Li-ion migration through the planar pathway indicates that two amide carbonyl groups from two different molecules (P2) interact with the Li-ion more effectively than the amide carbonyl groups from the same molecule (P1) (Fig. 7f). The relative energy of the P2 geometry (−21.0 kcal mol^−1^) is much lower than that of the P1 state (Fig. 7c). The migration of Li-ion through the terpyridine binding sites both for axial and planar pathways leads to one optimized geometry, where Li-ion is sandwiched between two terpyridine units and interacts simultaneously with the nitrogen atoms (Fig. 7g). Notably, the relative energy of the axial migration (Ty-A, −82.3 kcal mol^−1^) is much lower than that of the planar migration (Ty-P, −55.0 kcal mol^−1^) (Fig. 7c). Thus, the notable finding is that for both interaction sites the hopping of the Li-ions through the axial pathway is more preferred in comparison with the planar pathway (Fig. 7c). Among the two interaction sites the hopping of Li-ion through the terpyridine sites is more ideal in comparison with amide sites.

For solid conductors, Li-ion concentration and ionic mobility in the nanostructure are two dominant factors that govern the conductivity value. In general, solid conductors with higher Li-ion binding sites produce higher Li-ion concentrations in the framework.^19,46,55–57^ Likewise, the directed channel formed by the nanostructured morphologies *via* H-bonding offers a means of transport for free ions.^32,38,40^ The radial distribution function of the three different SONs revealed that the Li-ion concentration at the respective binding sites is highest for AM-5 followed by AM-6 and AM-4, respectively. Among the three different SONs, the most stable, well-defined rigid directional channel formed through H-bonding that favors fast Li-ion migration is responsible for the observed highest ion conductivity values for AM-6. Under identical conditions, the higher ion conductivity of the SONs of AM-5 in comparison with that of AM-4 indicates that the Li-ion concentration at the respective binding sites plays an important role in determining the conductivity value. The use of positively charged guanidinium core in the SONs of AM-4 promotes the ion pair splitting to produce free mobile Li-ions, responsible for the higher transference number in comparison with AM-5, whereas the structural superiority with a well-defined directional channel for SONs of AM-6 liable for higher transference number.

## Conclusions

This study presents, for the first time, a thorough fundamental analysis of rational design methodologies, showcasing their utility in precisely adjusting noncovalent interactions for effectively addressing the conventional trade-off between ionic conductivity and mechanical characteristics in SSEs. Our research led to the development of three solution processable SONs characterized by distinct supramolecular interactions, which were attained through structural modifications. These SONs function as solid-state Li-ion conductors, employing a strategy designed to enhance mechanical properties, promote ion-pair dissociation, and increase ion conductivity. Through the utilization of diverse spectroscopic and computational methodologies, an analysis of these SONs has been undertaken to establish their thermodynamic attributes, packing configurations, elemental compositions, and supramolecular interaction classifications. The effectiveness of the mechanical and Li-ion conducting characteristics is linked to various factors, including the quantity of hydrogen bonding sites, the ionic framework, and the interactions of π–π stacking. The research findings reveal that the accurate and structured directional H-bonding in the assembly of SONs is vital for reaching an optimal Young's modulus of 1050.5 ± 38 MPa and toughness of 15 666 ± 423 kJ m^−3^, thereby overshadowing the effect of the number of H-bonding sites. The comprehensive computational study indicated that the highly organized H-bonded architecture of SONs supported the development of binding pockets, thereby enhancing lithiation. Furthermore, the directional channels contributed to efficient Li-ion mobility, leading to the highest recorded ionic conductivity of 3.12 × 10^−4^ S cm^−1^, with a Li-ion transference number of 0.8 at 298 K. According to the molecular dynamics simulation, it is evident that, among the multiple interaction sites, the migration of Li-ions along the axial pathway is favored over the migration along the planar pathway. This study delineates the methodology that examines the effect of noncovalent interactions in nanoscale assemblies on the ion conductivity and mechanical features of supramolecular Li-ion conductors. More broadly, this research emphasizes the potential of integrating synthetic design with nanoscale assembly to create materials endowed with beneficial properties.^58–60^ It is our conviction that the strategy discussed herein, which employs supramolecular dynamic bonding to create stretchable ion conductors, opens up a valuable pathway for the fabrication of resilient materials designed for energy storage devices that are inherently stretchable.

## Author contributions

R. R. V. contributed to the synthesis, characterization and data analysis. S. S. S. was involved in electrochemical study and data analysis. A. S. and J. A. were involved in some characterization and mechanical study processes. S. K. supervised the electrochemical study. R. L. contributed to the computational study. A. K. M. proposed the idea, designed the experiments, and carried out data analysis, overall supervision, and final drafting of the manuscript. The manuscript was written through contributions of all authors. All authors have given approval to the final version of the manuscript.

## Conflicts of interest

There are no conflicts to declare.

## Supplementary Material

SC-OLF-D5SC00159E-s001

## Data Availability

All experimental data are available in the ESI.†
