# Supplementary material for: Lithium-ion conducting self-assembled organic nanowires: optimizing mechanical performance and ionic conductivity through programmable supramolecular interactions
Source: Chem Sci. 2025 Jun 3;16(27):12534–47. doi: 10.1039/d5sc00159e (PMC12152969; doi:10.1039/d5sc00159e)
Supplement: SC-016-D5SC00159E-s001 [file SC-016-D5SC00159E-s001.pdf]

Supplementary Information (SI) for Chemical Science.
This journal is © The Royal Society of Chemistry 2025

Supplementary Information

Lithium-Ion Conducting Self-Assembled Organic Nanowires: Optimizing Mechanical Performance and Ionic Conductivity through Programmable Supramolecular Interactions.

Vishwakarma Ravikumar Ramlal, Sam Sankar Selvasundarasekar, Akanksha Singh, Jenil Ankola, Rabindranath Lo, Subrata Kundu, * Amal Kumar Mandal.**

V. R. Ramlal, A. Singh, J. Ankola, A. K. Mandal.
Analytical and Environmental Science Division and Centralized Instrument Facility,
CSIR-Central Salt and Marine Chemicals Research Institute,
Bhavnagar, Gujarat-364002, India.
Email: akmandal@csmcri.res.in

V. R. Ramlal, A. Singh, J. Ankola, A. K. Mandal.
Academy of Scientific and Innovative Research (AcSIR), Ghaziabad-201 002, India.

S. S. Selvasundarasekar, S. Kundu.
Electrochemical Process Engineering (EPE) Division,
CSIR-Central Electrochemical Research Institute (CECRI),
Karaikudi, Tamil Nadu, 630003, India.
E-mail: skundu@cecri.res.in

R. Lo.
Institute of Organic Chemistry and Biochemistry,
Czech Academy of Sciences Flemingovo náměstí 542/2, Prague 160 00, Czech Republic.
Email: rabindranath.lo@uochb.cas.cz

No	Table of Contents	Page No.
1.	Materials and Characterization	3
2.	Synthesis routs of AM-4, AM-5 and AM-6	4
3.	Detailed synthesis methodology	4
4.	^{1}H NMR, ^{13}C NMR, IR and MS spectra of (1)	8
5.	^{1}H NMR, ^{13}C NMR, IR and MS spectra of (2)	10
6.	^{1}H NMR, ^{13}C NMR, IR and MS spectra of (3)	11
7.	^{1}H NMR, ^{13}C NMR, IR and MS spectra of (AM-4)	14
8.	^{1}H NMR, ^{13}C NMR, IR and MS spectra of (4)	15
9.	^{1}H NMR, ^{13}C NMR, IR and MS spectra of (5)	17
10.	^{1}H NMR, ^{13}C NMR, IR and MS spectra of (6)	19
11.	^{1}H NMR, ^{13}C NMR, IR and MS spectra of (7)	21
12.	^{1}H NMR, ^{13}C NMR, IR and MS spectra of (AM-5)	23
13.	^{1}H NMR, ^{13}C NMR, IR and MS spectra of (AM-6)	25
14.	UV−vis and fluorescence response	27
15.	Determination of the degree of aggregation (α_{agg})	29
16.	Temperature dependent UV-Vis study	30
17.	Computationally optimized geometries	31
18.	XPS of SONs of AM-4, AM-5 and AM-6	33
19.	TGA profile for SONs of AM-4, AM-5 and AM-6	33
20.	Mechanical study of the composite films	33
21.	Sample preparation for Li-ion conductivity	34
22.	Details of conductivity measurements	34
23.	Conductivity data	35
24.	Comparable table of Li-ion conductivity data	36
25.	Calculation of Li-ion transference number (t_{Li+})	36
26.	Electrochemical stability of SONs	38
27.	Computational details	39
28.	SEM images of the composite films	40
29.	PXRD pattern of the composite films	40
30.	X-ray refinements and sample preparation for PXRD measurements	40
31.	Reference	42

1. Materials and Characterization:

Materials: Chemicals like N-bromosuccinimide (NBS), benzoylperoxide (BPO), 2-Acetyl Pyridine, 4-methyl benzaldehyde, potassium phthalimide, liquid ammonia, potassium hydroxide, oxalyl chloride, EDC (1-Ethyl-3-(3-dimethylaminopropyl)carbodiimide), gallic acid, sodium iodide, HOBT(1-Hydroxybenzotriazole), 1-bromododecane, alanine, potassium carbonate, triphenyl phosphine, hydrazine hydrate, 1,3,5-benzenetricarboxylic acid, Europium nitrate hexahydrate, terbium nitrate hexahydrate, lithium aluminium hydride, sodium hydroxide, triethylamine, purchased from commercial suppliers and used directly without further purification. Solvents such as CCl_4, dimethyl formamide (DMF), methanol (MeOH), ethanol (EtOH), chloroform ($CHCl_3$) acetone and hydrochloric acid procured from commercial sources. All solvents were of reagent grade, which were further dried and distilled prior to use following standard procedures. Anhydrous conditions achieved by double-neck flasks by flaming with a heat gun under vacuum and then purging with Argon. The inert atmosphere was maintained using N_2-filled balloons equipped with a syringe and needle that was used to penetrate the silicon stoppers used to close the flasks necks. Additions of liquid reagents were performed using glass syringes. A Barnstead System, U.S., employed to get high purity nanopure water for making all the aqueous solutions.

Characterization: *Thin layer chromatography* (TLC) was conducted on pre-coated aluminium sheets with 0.20 mm *Machevery-Nagel* Alugram SIL G/UV254 with fluorescent indicator UV254.
Column chromatography was carried out using *Spectrochem* silica gel 60 (particle size 100-200 µm).
Nuclear magnetic resonance (NMR) (^1H, and ^{13}C) spectra were obtained on a 500 MHz NMR (*Jeol JNM EX-400*) or 500 MHz (Bruker 500 MHz FT NMR, model: Advance-DPX 500). Chemical shifts were reported in ppm according to tetramethylsilane using the solvent residual signal as an internal reference. Coupling constants (*J*) were given in Hz. Resonance multiplicity was described as *s* (singlet), *d* (doublet), *t* (triplet), *dd* (doublet of doublets), *dt* (doublet of triplets), *td* (triplet of doublets), *q* (quartet), *m* (multiplet) and *bs* (broad signal). Carbon spectra were acquired with a complete decoupling for the proton.
Liquid chromatography-mass spectrometry (LC-MS) measurements were conducted on an *Agilent 6200 series TOF mass spectrometer* equipped with ESI and APCI ionization sources and a Time of Flight (TOF) detector, operating in positive mode. The analytes solution was delivered to the ESI or APCI source by an *Agilent 1200 series LC system* at a flow rate of 0.25 ml/min. The typical elution gradient starts from H_2O (90%) to CH_3CN (100%) for 20 minutes. ESI mode: Typical ESI conditions were capillary voltage 2.0 kV; cone voltage 65 V; source temperature 150 °C; desolvation temperature 250 °C; drying gas 5 L/min, nebulizer 60 psig. APCI: Typical APCI conditions were, capillary voltage 2.0 kV; cone voltage 65 V; source temperature 250 °C; desolvation temperature 350 °C; drying gas 5L/min; nebulizer 60 psi. Dry nitrogen was used as the ESI and APCI gas.
Parkin Elmer 883 spectrometer was used to record the FT-IR data using the KBr pellet. Using a Zetasizer Nano-ZS90 (Malvern) instrument with a 632.8 nm He-Ne laser DLS, experiments were carried out at 298 K.
Melting point: The melting point was conducted using a 10002D MEL-TEMP apparatus while heating from room temperature.

Transmission electron microscopy images were collected using a JEOL JEM-2100 electron microscope working at 200 kV accelerating voltage. The samples were prepared on the surface of lacey-carbon-supported copper TEM grids.

The JEOL JSM-7100F instrument working at 18 kV accelerating voltage was used to record the FE-SEM data. Before taking the FE-SEM images, the thin coating of Au (~ 4 nm) was coated using a vacuum evaporator.

Powder X-ray diffraction (PXRD) patterns were recorded at room temperature on a Philips X'pert X-ray powder diffractometer using Cu-Ka radiation (λ=1.5418 A) in the 2θ range of 5–50º.

X-ray photoelectron spectra (XPS) were obtained from a Thermo-Scientific NEXSA. Source Properties: The NEXSA uses very low-energy X-rays (Al K-Alpha). Energy: 1486.6 eV. For survey spectra, a pass energy of 200 eV and step size of 1 eV is used. For higher energy resolution, in narrow scan spectra, a pass energy of 50 eV with a step size of 0.1 eV is used. Vacuum Details: Operated in the Ultra-High Vacuum (UHV) range from 10-8 to 10-10 mbar. Charge Correction: C 1s (reference) - 284.8 eV.

Thermo-gravimetric analysis (TGA) was conducted using an Auto TG 209 (F1) apparatus under a nitrogen flow of 100 mL min^{-1} while heating from room temperature.

UV-Vis absorption spectra were recorded using Shimadzu crop 80109 UV- Vis spectrophotometer.

Fluorescence Spectroscopy was carried out on a Horiba QM-400 spectrophotometer.

The conductivity study has been done using the electrochemical work station (VSP Biologic, France).

Mechanical property: The Xforce P tensile testing machine (Zwick/Roell, Germany), with a sensitivity of approximately 2mV/V and a force measurement capacity of up to 500N, was utilized to evaluate the mechanical properties.

2. Synthesis routs of AM-4, AM-5 and AM-6:

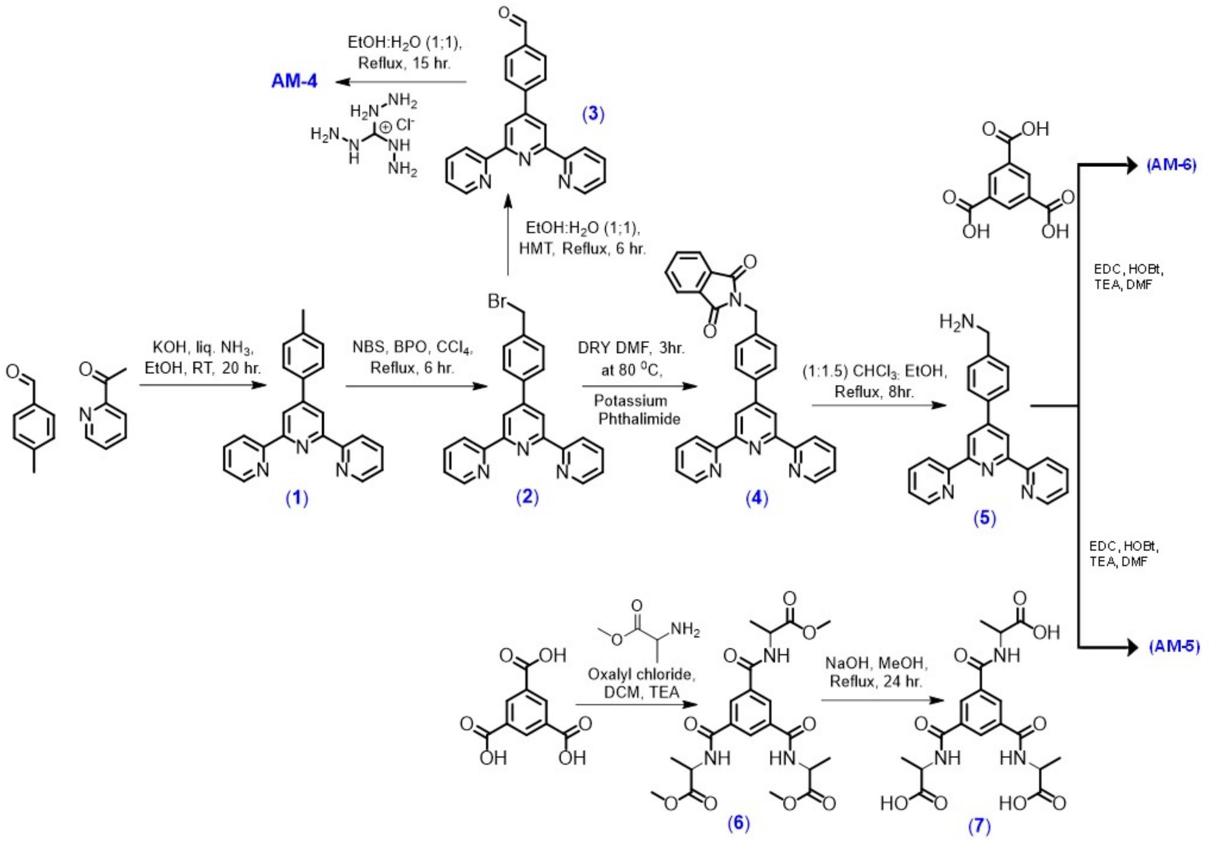

Figure S1 Methodology adopted for the synthesis of AM-4, AM-5, and AM-6.

3. Detailed synthesis methodology:

Synthesis of (1): In an oven-dried 250 mL double-neck round bottom flask, 4-methyl benzaldehyde (2.12 g, 17.65 mmol) and 2-acetyl pyridine (4.27 g, 35.31 mmol) were dissolved in 100 mL of ethanol. To this stirred solution, KOH (3.07 g, 54.24 mmol) in 58 mL of liquid ammonia was added dropwise. The reaction mixture was stirred at room temperature for 20 hrs. Off-white precipitates formed in the reaction mixture were filtered through a G3 crucible and washed 3-4 times with cold ethanol and finally with ether. The residue was dried in a vacuum to isolate the desired product as an off-white solid. Yield (4.12 g, 72.28 %): ^{1}H NMR (500 MHz, $CDCl_3$, δ ppm): 8.75 (4H, *bs*, H^5, H^1); 8.69 (2H, *d*, J = 5.0 Hz, H^4); 7.89 (2H, *dt*, J = 7.5 Hz, H^2); 7.85 (2H, *d*, J = 5.0 Hz, H^6); 7.37 (2H, *t*, J = 7.5 Hz, H^3); 7.34 (2H, *d, J* = 5.0 Hz, H^7) 2.45 (3H, s, H^8). ^{13}C NMR (125 MHz, $CDCl_3$, δ ppm): 156.3, 155.8, 150.1, 149.1, 139.7, 136.8, 135.4, 129.6, 127.3, 123.7, 121.3, 118.6, 21.2. IR (KBR, cm^{-1}): 3224.87, 3011.18, 1587.46, 1557.93, 1469.55, 1394.67, 1265.63, 819.90, 779.76, and 732.83. ESI-MS: calc. for $C_{22}H_{17}N_3$: 323.14, Found 324.15 $[M+H]^+$. Melting Point: 155 -158℃.

Synthesis of (2): In an oven-dried 250 mL double neck round bottom flask, compound (1) (2.00 g, 6.19 mmol), NBS (1.66 g, 9.32 mmol) and BPO (0.06 g, 0.24 mmol) were dissolve in anhydrous CCl_4 (50 mL) and the reaction mixture was purged with nitrogen gas for 15 mints. Then the reaction mixture was stirred and refluxed for 6 hrs. The reaction mixture was filtered through a G3 crucible, evaporating the filtrate in a rotary evaporator. The crude reaction mixture was extracted three times (3 × 30 mL) with $CHCl_3$. Collected combined organic layer was dried with anhydrous $MgSO_4$, The combined organic layer was removed by evaporation, and the crude product was recrystallized in *n*-hexane to produce a light yellow solid as a product (2.05 g, yield: 82.41%). ^{1}H NMR (500 MHz, $CDCl_3$, δ ppm): 8.73 (4H, *bs*, H^5, H^1), 8.68 (2H, *d*, J = 10.0 Hz, H^4), 7.92-7.87 (4H, *m*, H^2, H^6), 7.55 (2H, *d*, J = 10.0 Hz, H^7), 7.36 (2H, *t*, J = 5.0 Hz H^2), 4.57 (2H, *s*, H^8). ^{13}C NMR (125 MHz, $CDCl_3$, δ ppm): 156.0, 155.9, 149.4, 149.1, 138.6, 138.5, 136.9, 129.6, 127.7, 123.9, 121.3, 118.8, 32.9. IR (KBR, cm^{-1}): 3411.78, 3052.31, 3014.18, 1602.82, 1585.01, 1467.69, 1388.38, 1222.54, 1077.02, 788.10, and 741.24. ESI-MS: m/z calc. for $C_{22}H_{16}BrN_3$: 401.05, Found 402.06 $[M + H]^{+\cdot}$. Melting Point: 148 -152 ℃.

Synthesis of (3): In an oven-dried 250 mL double-neck round bottom flask, compound (2) (2.00 g, 4.98 mmol) and hexamethylenetetramine (2.09 g, 14.96 mmol) in a mixture of EtOH / H_2O (v/v = 1:1) (60 ml) was purged with nitrogen gas. The reaction mixture was stirred and refluxed for 6 hrs, and then 10 ml of conc. HCl was added, followed by reflux for another 30 min. The reaction mixture was cooled to room temperature and neutralized with $NaHCO_3$ solution. The neutralized reaction mixture was extracted three times (3 × 30 mL), and the combined organic layer was dried over anhydrous $MgSO_4$. The crude product was purified by precipitate out in diethyl ether to give a light-yellow solid as a product. (Yield: 0.60 g, 35.72%): ^{1}H NMR (500 MHz, $CDCl_3$, δ ppm): 10.03 (1H, *s*, H^8), 8.69 (2H, *s*, H^5), 8.67 (2H, *d*, J =5.0 Hz, H^4), 8.62 (2H, *d*, J = 10.0 Hz, H^1), 8.00 (4H, *dd*, J = 10.0 Hz, H^7, H^6), 7.83 (2H, *t*, J = 7.5 Hz, H^2), 7.31 (2H, *t*, J = 5.0 Hz, H^3). ^{13}C NMR (125 MHz, $CDCl_3$, δ ppm): 190.8, 155.2, 154.8, 148.1, 147.8, 143.4, 135.9, 135.4, 129.2, 127.0, 123.4, 120.4, 118.0. IR (KBR, cm^{-1}): 3413.51, 2323.23, 1695.50, 1604.10, 1585.36, 1567.39, 1468.46, 1387.44, 1212.06, 1172.92, 1038.27, 827.22, 788.01, and 732.31. ESI-MS: m/z calc. for $C_{22}H_{15}N_3O$: 337.12, Found 338.12 $[M + H]^+$. Melting Point: 165 -170℃.

Synthesis of (AM-4): In an oven-dried 100 mL double-neck round bottom flask, compound (3) (0.50 g, 1.48 mmol) in ethanol (50 ml) was refluxed for 50 minutes to dissolve. Trihydrazineylmethane chloride (65 mg, 0.46 mmol) in 10 ml water was added to the reaction mixture, and the mixture was refluxed for 15 hrs. Yellow precipitate appeared after completion of the reaction. The precipitate was filtered at room temperature and washes the precipitate several times with water and ethanol. Drying the precipitate in a vacuum yielded a yellow solid as a product. (Yield: 0.60 g, 35.72%). ^{1}H NMR (500 MHz, DMSO-*d*$_6$, δ

ppm): 8.80-8.77 (12H, *m*, H[5], H[4]), 8.75-8.66 (9H, *m*, H[1], H[8]), 8.16-8.03 (18H, *m*, H[7], H[6], H[3]), 7.56 (6H, *t*, *J* = 5.0 Hz, H[2]). ^{13}C NMR (125 MHz, DMSO-d_6, δ ppm): due to very low solubility, we are unable to record the ^{13}C NMR spectra after several attempts. IR (KBR, cm^{-1}): 3422.15, 2923.66, 1642.07, 1586.70, 1468.87, 1390.97, 1261.38, 1094.86, 1042.54, 788.28, 701.95, and 659.93. ESI-MS: m/z calc. for $C_{67}H_{48}ClN_{15}$: 1097.39. Found 1062.16 $[M – Cl]^+$. Melting Point: 218-220℃.

Synthesis of (4): In an oven-dried 100 mL double neck round bottom flask, compound (2) (0.10 g, 0.29 mmol), potassium phthalimide (0.05g, 0.29 mmol) in Dry DMF (20 mL) was purged with nitrogen gas. The reaction mixture was stirred and refluxed for 3 hrs. The reaction mixture was cooled down to room temperature. Immediately white precipitate appeared adding 50 ml of chloroform to the reaction mixture. The white precipitate was filtered and washed with 100 ml of chloroform. The organic portion was extracted with water and 0.1 M of NaOH. The combined organic layer was dried with anhydrous $MgSO_4$. The solvent was removed with a rotary evaporator and the crude product was precipitated out in diethyl ether. The desired product was obtained as a light yellow solid. (0.07 g, yield: 80.41%). ^{1}H NMR (500 MHz, $CDCl_3$, δ ppm): 8.73-8.72 (2H, *m*, H[4]), 8.71 (2H, *s*, H[5]), 8.67-8.66 (2H, *d*, H[1]), 7.90-7.87 (6H, *m*, H[3], H[9], H[10]), 7.74-773 (2H, *m*, H[2]), 7.60-7.58 (2H, *d*, *J* = 5 MHz, H[6]) 7.37-7.34 (2H, *dd*, H[7]), 4.94 (2H, *s*). ^{13}C NMR (125 MHz, $CDCl_3$, δ ppm): 166.9, 155.0-155.8, 148.6, 148.0, 137.5, 132.9, 131.0, 128.1, 126.5, 122.7, 122.3, 120.2, 117.7, 40.2. IR (KBR, cm^{-1}): 3130.34, 2922.75, 2851.92, 1717.42, 1590.56, 1457.04, 1399.79, 1337.75, 1226.95, 1125.33, 1013.35, 763.96, and 723.37. ESI-MS: m/z calc. For $C_{30}H_{20}N_4O_2$: 468.12, Found 468.90 $[M\ ]^{+\cdot}$ Melting Point: 232 -234℃.

Synthesis of (5): In an oven-dried 100 mL double neck round bottom flask, compound (3) (0.10 g, 0.21 mmol) was dissolved in $CHCl_3$/EtOH (1:1.5 v/v, 25 mL) and the reaction mixture was purged with nitrogen gas. Hydrazine (55%, 0.3 ml, 5.00 mmol) was added to it and the reaction mixture was stirred and refluxed for 8 hrs. After completion of the reaction the mixture was cooled down to room temperature and 50 ml of $CHCl_3$ was added to the reaction mixture. The obtained white precipitate was filtered and washed with 100 ml of $CHCl_3$. The organic portion was extracted with water and 1 M NaOH solution. Combined organic layers were dried over anhydrous $MgSO_4$. After removing the solvent from the rotary evaporator the desired product was obtained as a light yellow solid (0.07 g, yield: 70.51%). ^{1}H NMR (500 MHz, $CDCl_3$, δ ppm): 8.67 (4H, *bs*, H[5], H[4]), 8.61-8.60 (2H, *d*, *J* = 5.0 Hz, H[1]), 7.84-7.80 (4H, *m*, H[3,6]), 7.40-7.38 (2H, *d*, *J* = 10.0 Hz, H[7]), 7.30-7.28 (2H, m, H[2]), 3.89 (2H, *s*, H[8]). 1.68 (2H, *bs*),^{13}C NMR (125 MHz, $CDCl_3$, δ ppm):155.2, 154.8, 148.9, 148.1, 143.0, 135.8, 126.6, 126.4, 122.8, 120.3, 117.7, 45.0. IR (KBR, cm^{-1}): 3056.57, 2923.02, 2364.16, 1658.52, 1586.94, 1078.01, 1466.02, 1391.43, 1264.5, 1078.01, 1051.1, 988.01, 825.35, 794.07, 742.94 ESI-MS: m/z calc. For $C_{22}H_{18}N_4$: 338.15, Found 339.08 $[M + H]^{+\cdot}$. Melting Point: 120 -122℃.

Synthesis of (6): In an oven-dried 100 mL double-neck round bottom flask, benzene-1,3,5-tricarboxylic acid (0.50 g, 2.38 mmol) dissolve in dry DCM, and placed in an ice bath to maintain 0℃-5℃. To this solution, oxalyl chloride (1.22 ml, 14.28 mmol) and 2-3 drop of DMF as a catalyst were added. The reaction mixture was stirred at room temperature for 8 hrs under N_2 ATM. After this time the reaction mixture evaporates immediately at reduced pressure to remove excess oxalyl chloride. The crude product was dissolved in dry DCM and maintain an inert atmosphere. To the stirred solution, D-alanine methyl ester hydrochloride (1.99 g, 1.4.28 mmol) was added dropwise at ice-cold conditions. The reaction mixture was stirred for 24 hrs at room temperature. After completion of the reaction, the solvent was evaporated under a vacuum. The crude product was extracted thrice with CH_2Cl_2 (3 x 50 mL). The combined organic layer was dried with anhydrous Na_2SO_4 and evaporated under reduced pressure. The crude product was precipitated out using diethyl ether to obtain the desired product as a white solid. (0.31g, yield: 28.00%). ^{1}H NMR (500 MHz, $CDCl_3$, δ ppm): 8.20 (3H, *s*, H[5]), 7.55 (3H, *d*, *J* = 5.0 Hz, H[4]), 4.75 (3H, *m*, H[2]), 3.80 (9H, *s*, H[3]), 1.59 (9H, *d*, *J* =10.0 Hz, H[1]). ^{13}C NMR (125 MHz, $CDCl_3$, δ ppm): 174.0, 166.1, 135.1, 128.9, 52.7, 49.0, 17.6. IR (KBR, cm^{-1}): 3130.97, 1750.75, 1643.31, 1562.30,

1400.70, 1214.50, 1164.47, 1131.34, 1055.13, and 690.56. ESI-MS: m/z calc. for $C_{21}H_{27}N_3O_9$: 466.17, Found 488.16 [M + Na]$^+$. Melting Point-280 - 282 C.

Synthesis of (7): In a two-neck 100 mL round bottom flask, compound (7) (0.30 g, 0.64 mmol) was dissolved in 30 ml of methanol. Then conc. aqueous NaOH solution (3 ml) was added dropwise to the stirred solution and the reaction mixture was refluxed for 20 hrs at 65° C. Then the reaction mixture was cooled down to room temperature and acidified with an aqueous solution of 1M HCl to achieve pH (2-3). The obtained white precipitate was filtered through a G3 crucible. The precipitate was washed several times with water to remove excess acid. Then the precipitate was dried in a vacuum desiccator. The desired product was obtained as a white solid (Yield: 0.21g, 68.06 %). ^{1}H NMR (500 MHz, DMSO *d*^{6}, *δ* ppm): 12.58 (3H, *s*, H^1). 4.48-4.43 (3H, *q*, H^2), 1.43-141 (9H, *d*, H^3), 8.99-8.98 (3H, *d*, H^4), 8.48 (3H *s*, H^5). ^{13}C NMR (125 MHz, $CDCl_3$, *δ* ppm): 173.1, 164.8, 133.7, 128.5, 47.7, 16.2. IR (KBR, cm^{-1}): 3056.57, 2923.02, 2364.16, 1658.52, 1586.94, 1078.01, 1466.02, 1391.43, 1264.5, 1078.01, 1051.1, 988.01, 825.35, 794.07, 742.94 ESI-MS: m/z calc. $C_{18}H_{21}N_3O_9$: 423.15, Found 423.39. Melting Point-270 - 272 C.

Synthesis of (AM-5): In an oven-dried 100 mL double neck round bottom flask under nitrogen atmosphere, compound (4) (0.05 g, 0.11 mmol) was dissolved in dry DMF and placed in an ice bath to maintain 0℃-10℃. Then HOBt (72 mg, 0.047mmol) was added and the reaction mixture was stirred for 20 min. After this time compound (8) (0.15 g, 0.47 mmol), EDC.HCl (0.13 g, 0.70 mmol) and trimethylamine (0.1 mL) were added to this solution. The reaction mixture was stirred at room temperature for 48 hrs under. After completion of the reaction, the solvent was evaporated under a vacuum. Then the reaction mixture was evaporated to remove the solvent. The crude product was dispersed in 20 ml of water and sonicated for 10 minutes. The obtained precipitate was filtered through a G3 crucible and washed several times with water. The precipitate was dried in a vaccume decicatore. The dried residue again was dispersed in 30 ml of chloroform and sonicated for 10 minutes to remove the unreacted starting materials. After filtration and drying of the obtained precipitates the desired product was obtained as a off white solid (Yield: 0.04 g, 19.18 %). ^{1}H NMR (500 MHz, DMSO- *d*^{6} δ ppm): 8.97 (3H, *bs*, H^9,), 8.74-8.73 (6H, *d*, *J* = 8.0 Hz, H^4), 8.66-(6H, *s*, H^5), 8.64-8.63 (8H, *dd*, *J* = 8.5 Hz, H^1), 8.03-8.00 (6H, *t*, *J* = 8.0 Hz H^3), 7.86-7.85 (6H, *d*, H^6). 7.52-7.43 (12H, *m*, H^{2,7}), 4.62-4.59 (3H, *t*, H^{11}), 4.41 (6H, *bs*, H^8), 1.45-1.44 (9H, *d*, H^{10}).^{13}C NMR (125 MHz, DMSO-*d*^{6}, *δ* ppm): 172.1, 165.2, 155.3, 154.6, 148.9, 148.8, 140.7, 136.8, 135.5, 134.0, 129.1, 127.6, 126.4, 124.1, 120.5, 117.4, 49.07, 41.4, 17.7. IR (KBR, cm^{-1}): 3280.23, 3056.31, 2979.45, 1644.91, 1585.56, 1537.75, 1518.37, 1388.75, 1263.37, 1039.13, 789.98, and 739.10. ESI-MS: m/z calc. $C_{84}H_{69}N_{15}O_6$: 1383.56, Found 1406.55 [M + Na$^+$]. Melting Point-272 - 274 C.

Synthesis of (AM-6): In an oven-dried 100 mL double neck round bottom flask under nitrogen atmosphere, compound (4) (0.05 g, 0.23 mmol) was dissolve in dry DMF and placed in an ice bath to maintain 0℃-10℃. Then HOBt (172 mg, 0.83 mmol) was added and the reaction mixture was stirred for 20 min. After this time compound (6) (0.28 g, 0.82 mmol), EDC.HCl (0.24 g, 0.1.3 mmol) and trimethylamine (0.2 mL) were added to this solution. The reaction mixture was stirred at room temperature for 48 hrs under. After completion of the reaction, solvent was evaporated under vacuum. Then reaction mixture was evaporated to remove solvent. The crude product was dispersed in 20 ml of water and sonicated for 10 minutes. The obtained precipitate was filtered through a G3 crucible and washed several times with water. The precipitate was dried in a vaccume decicatore. The dried residue again dispersed in 30 ml of chloroform and sonicated for 10 minutes to remove the unreacted starting materials. After filtration and drying of the obtained precipitates the desired product was obtained as a off white solid (Yield: 0.10 g, 75.53 %). ^{1}H NMR (500 MHz, DMSO- *d*^{6}, *δ* ppm): 9.44 (3H, *s*, H^9), 8.74-8.73 (6H, *d*, H^1), 8.70 (6H, *s*, H^5), 8.67-8.66 (6H, *d*, H^4), 8.61 (6H, *s*, H^{10}), 8.04-80.3 (6H, *t*, H^3),

7.94-7.92 (6H, *d*, H^6), 7.60-7.58 (6H, *d*, H^7), 7.52-5.50 (6H, *t*, H^2), 4.64-4.63 (6H, *s*, H^8). ^{13}C NMR (125 MHz, DMSO- d^6, δ ppm): 165.7, 155.7, 155.0, 149.3, 141.0, 137.5, 136.2, 135.0, 129.0, 128.5, 127.0, 124.5, 121.3, 117.1, 42.7. IR (KBR, cm^{-1}): 3785.34, 3129.54, 1652.08, 1585.34, 1543.04, 1457.76, 1400.015, 1288.5, 1265.31, 1115.81, 1039.42, 790.82, and 740.82. ESI-MS: m/z calc. $C_{75}H_{54}N_{12}O_3$: 1170.44, Found 1193.43 [M + Na^+]. Melting Point: 252-257 C.

4. ^1H NMR, ^{13}C NMR, IR and MS spectra of (1):

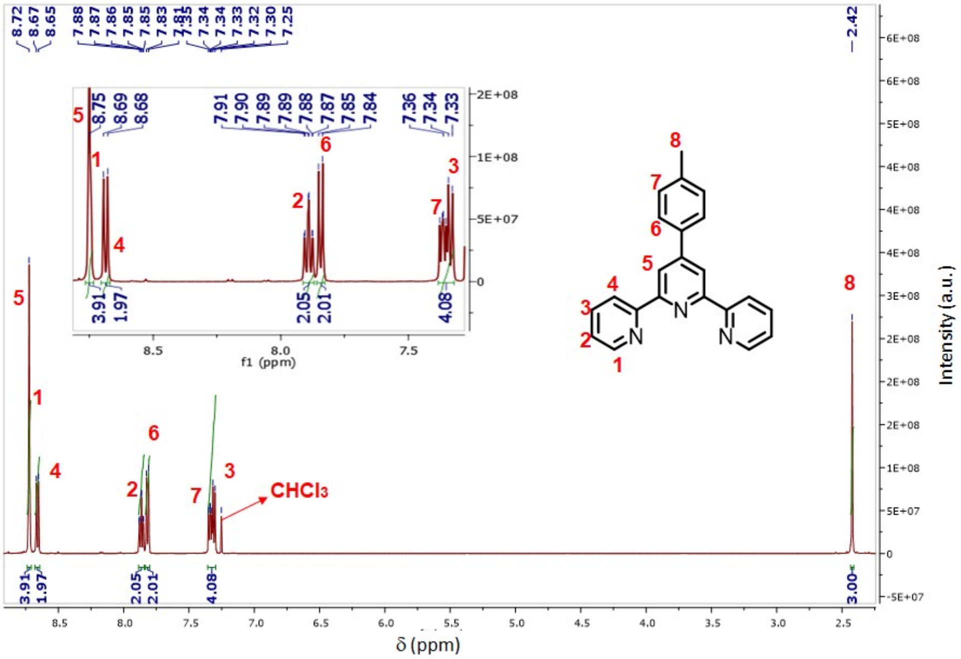

Figure S2a. ^1H-NMR (500 MHz, $CDCl_3$, 298K) spectrum for the compound (1) recorded at room temperature.

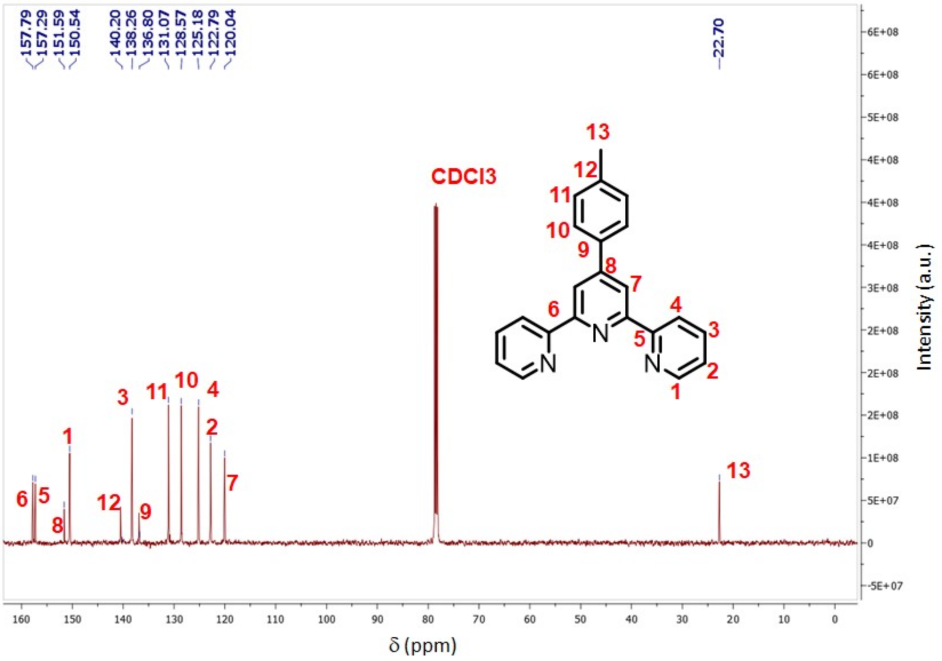

Figure S2b. ^{13}C-NMR (125 MHz, $CDCl_3$, 298K) spectrum for the compound (1) recorded at room temperature.

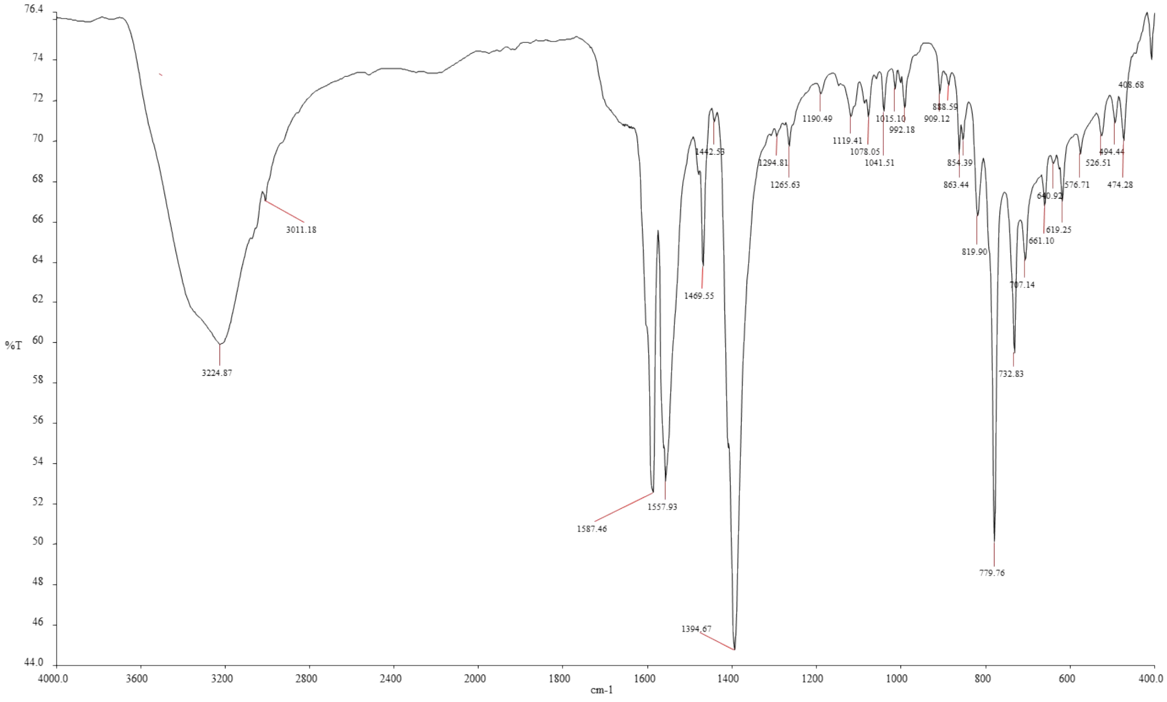

Figure S2c. IR spectrum of compound (1) recorded in solid state.

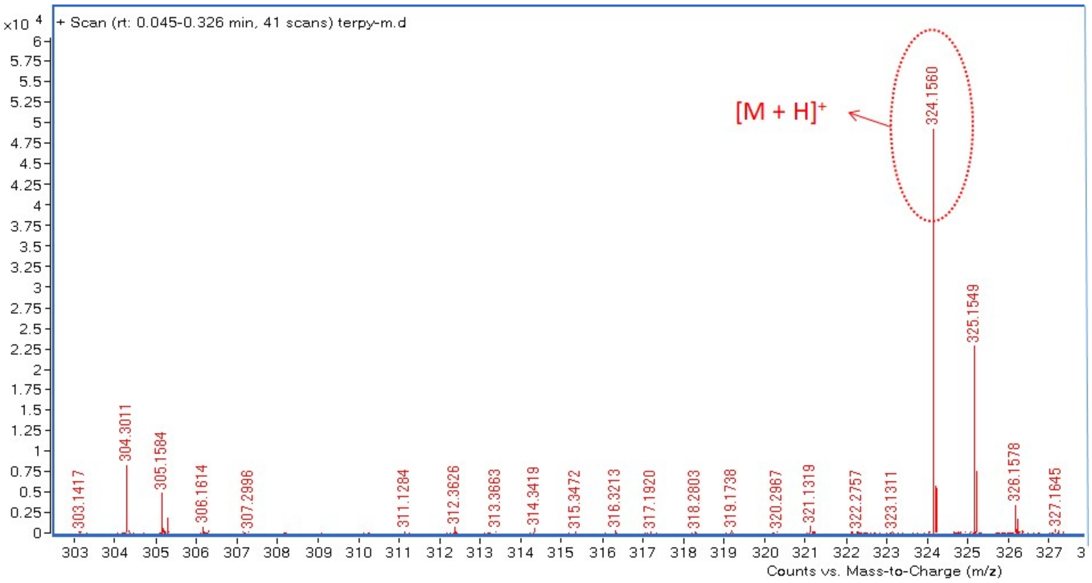

Figure S2d. LCMS of compound (1) recorded in (MeOH) solvent.

5. ^{1}H NMR, ^{13}C NMR, IR and MS spectra of (2):

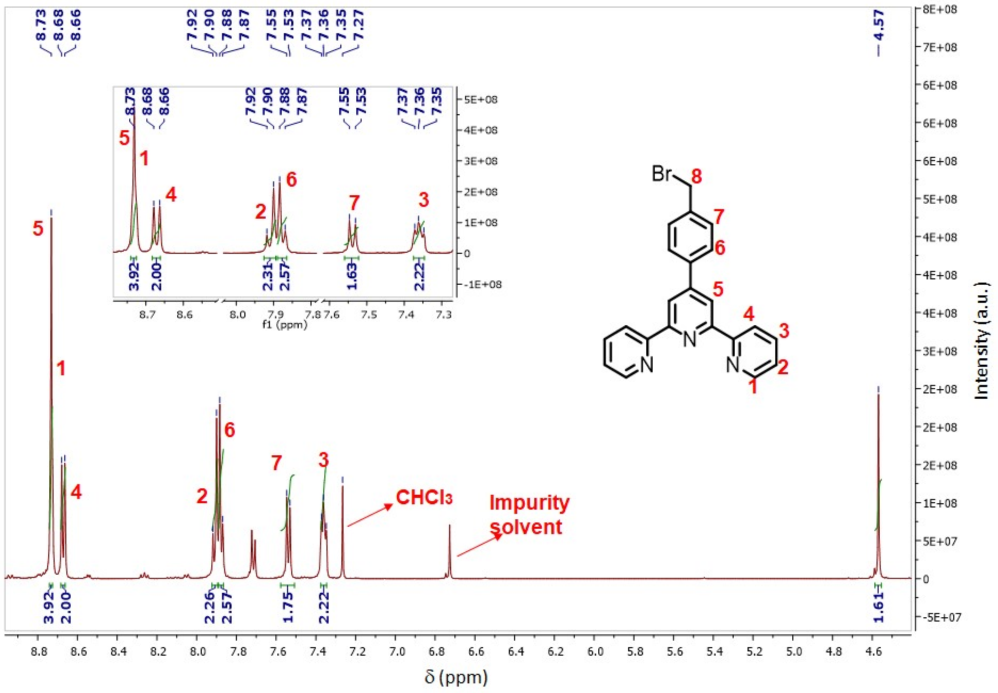

Figure S3a. ^{1}H-NMR (500 MHz, $CDCl_3$, 298K) spectrum for the compound (2) recorded at room temperature.

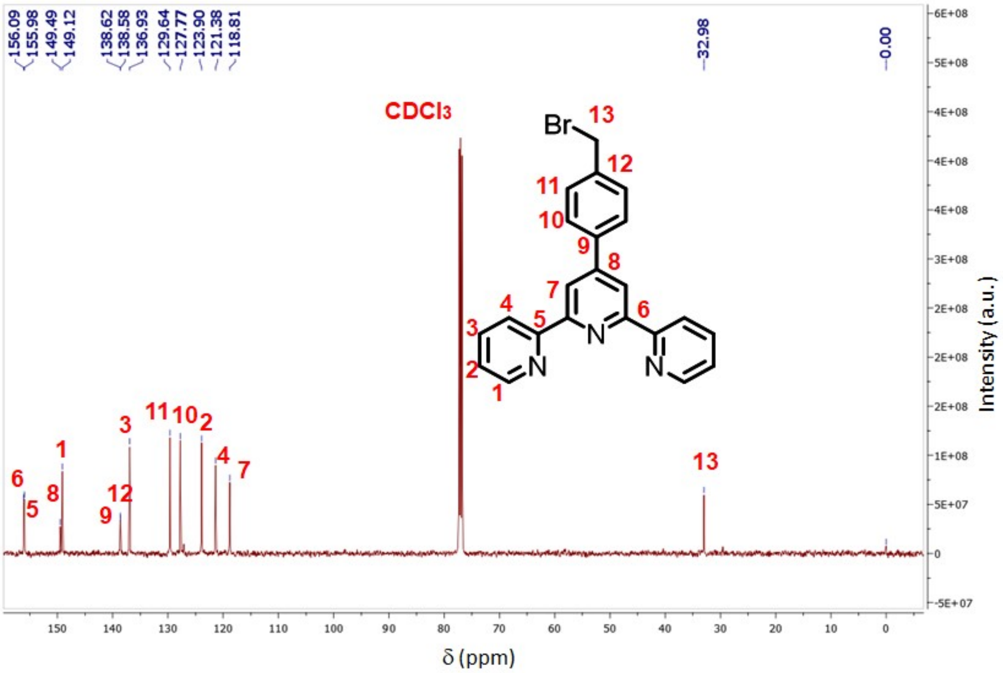

Figure S3b. ^{13}C-NMR (125 MHz, $CDCl_3$, 298K) spectrum for the compound (2) recorded at room temperature.

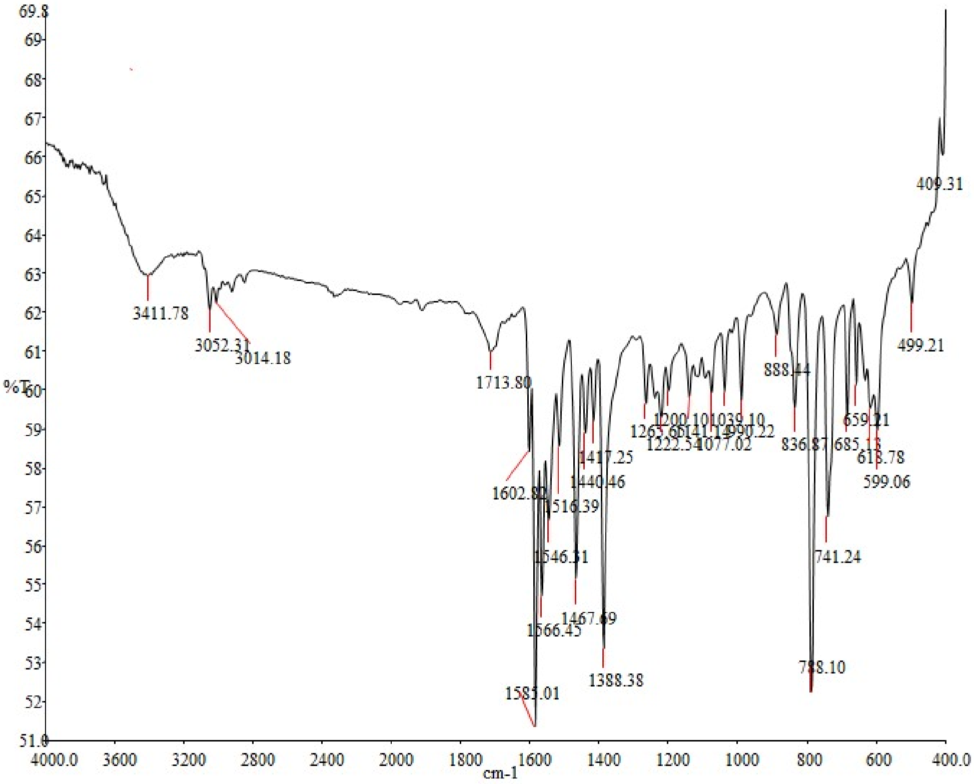

Figure S3c. IR spectrum of compound (2) recorded in solid state.

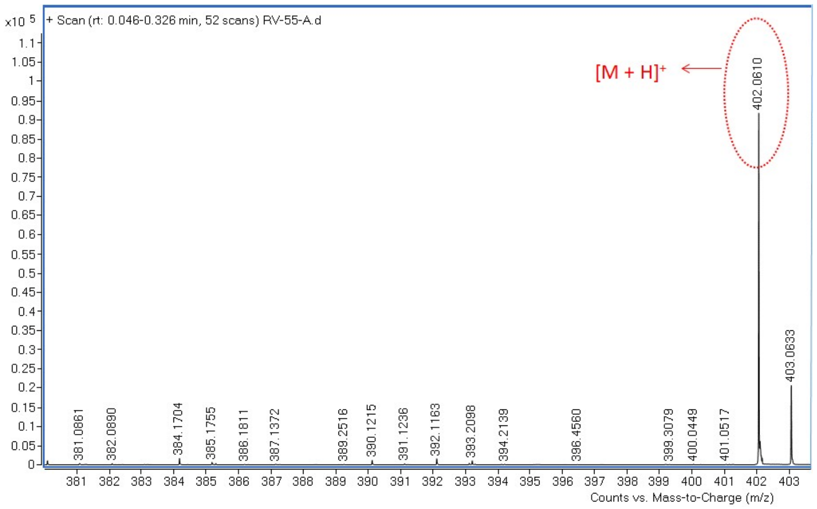

Figure S3d. LCMS of compound (2) recorded in (MeOH) solvent.

6. ^{1}H NMR, ^{13}C NMR, IR and MS spectra of (3):

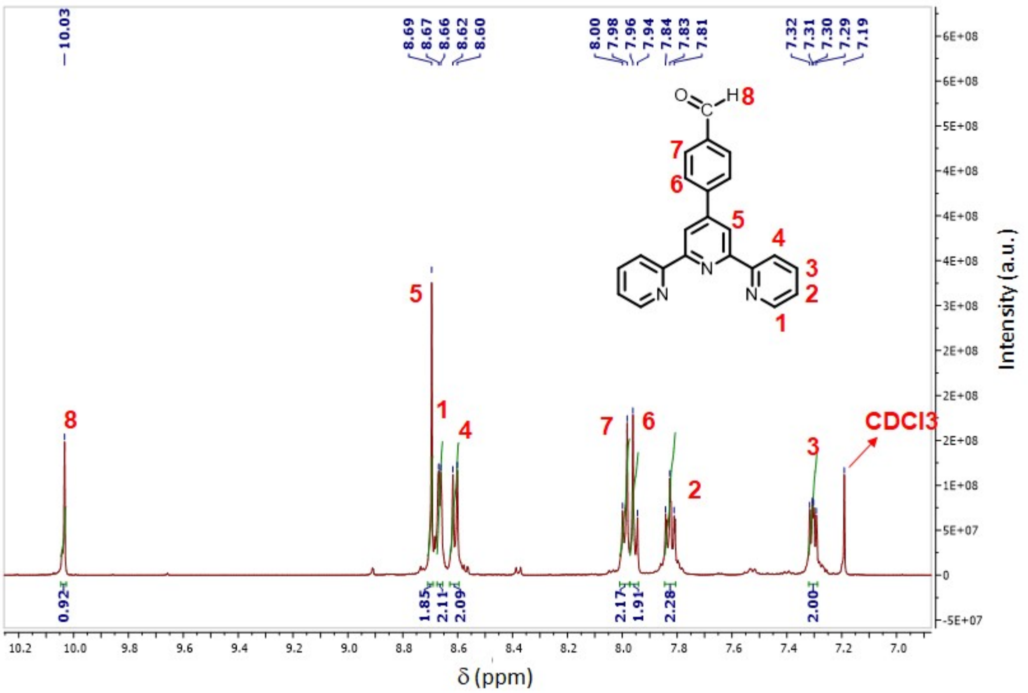

Figure S4a. ^{1}H-NMR (500 MHz, $CDCl_3$, 298K) spectrum for the compound (3) recorded at room temperature.

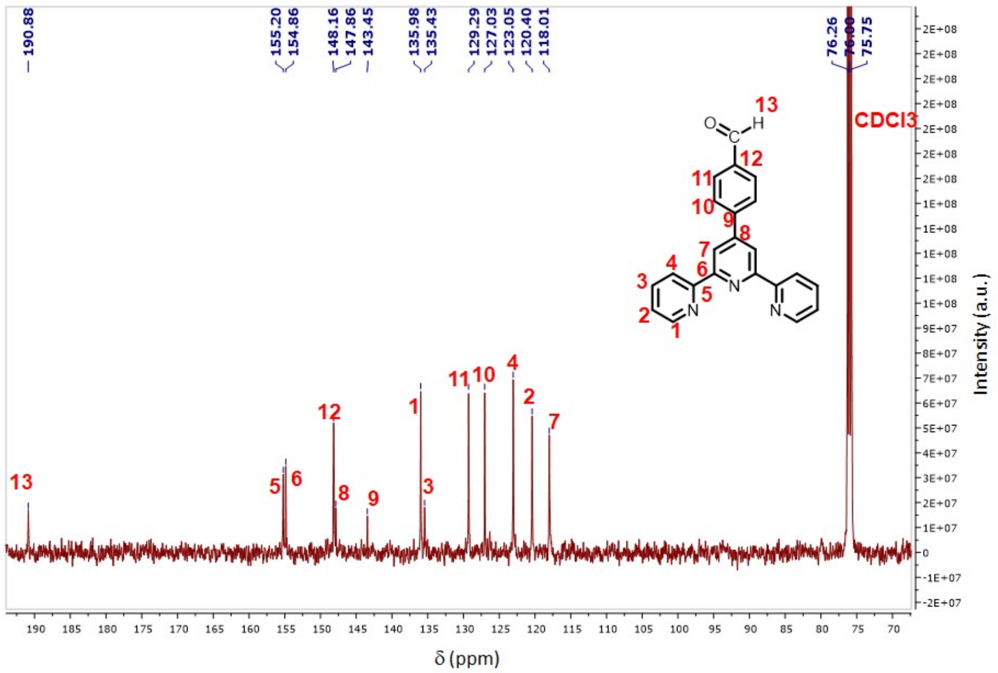

Figure S4b. ^{13}C-NMR (125 MHz, $CDCl_3$, 298K) spectrum for the compound (3) recorded at room temperature.

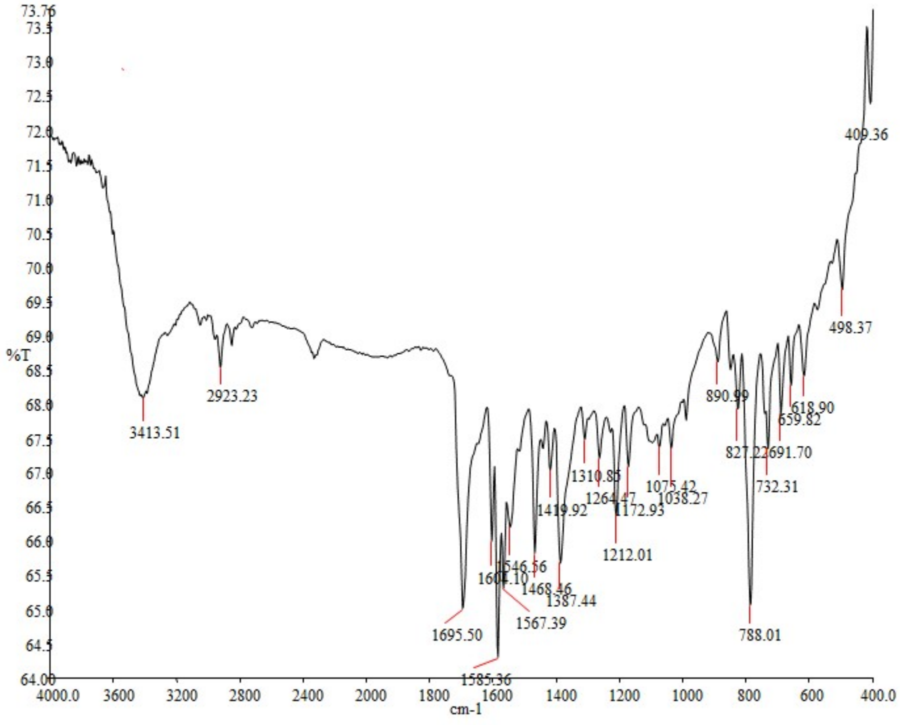

Figure S4c. IR spectrum of compound (3) recorded in solid state.

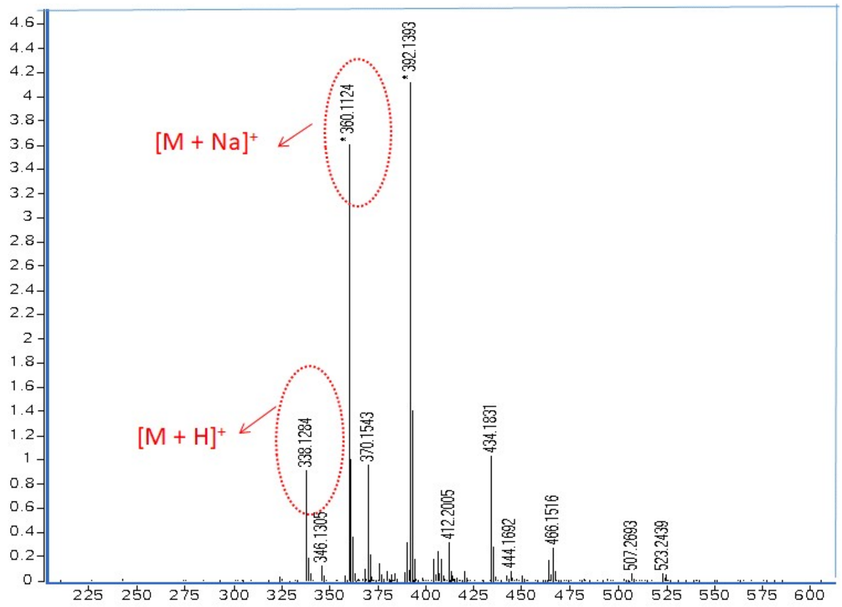

Figure S4d. LCMS of compound (3) recorded in (MeOH) solvent.

7. ^{1}H NMR, ^{13}C NMR, IR and MS spectra of (AM-4):

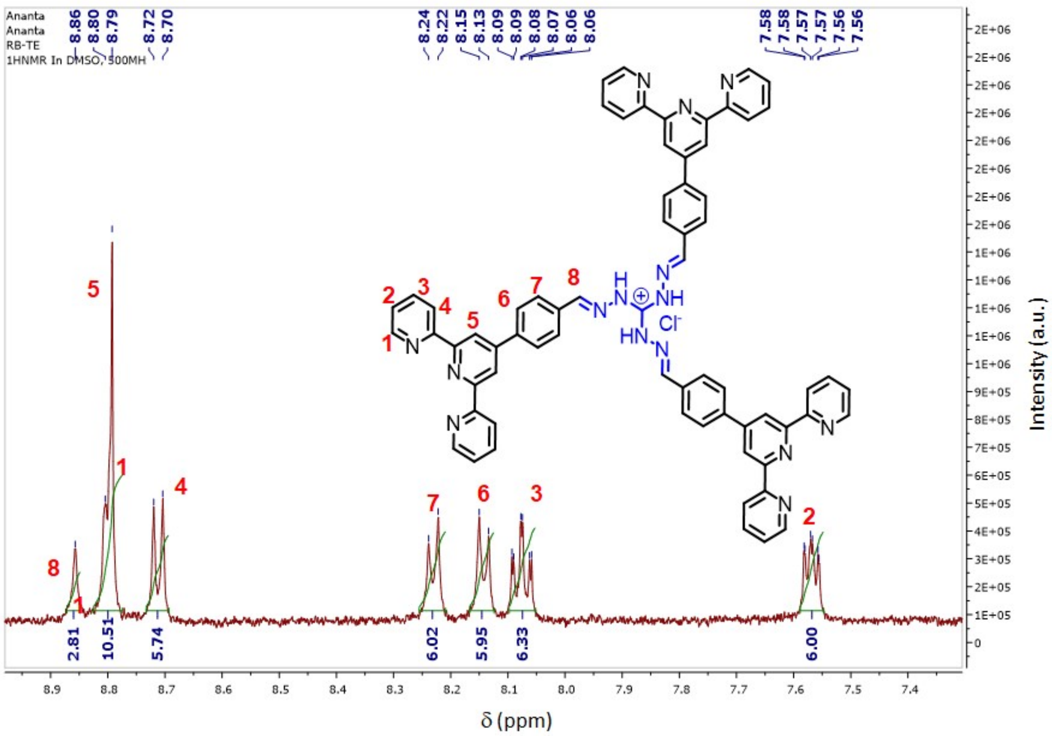

Figure S5a. ^{1}H-NMR (500 MHz, DMSO-d^{6}, 298K) spectrum for the compound (AM-4) recorded at room temperature.

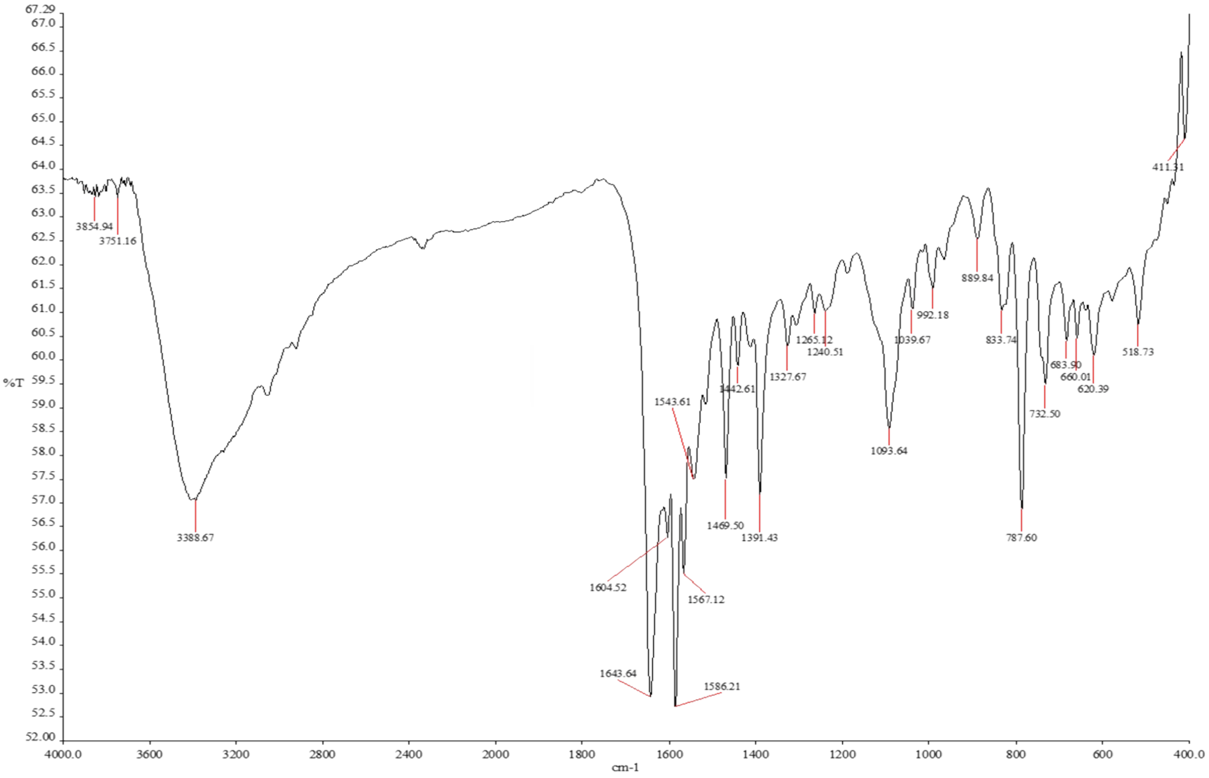

Figure S5b. IR spectrum of compound (AM-4) recorded in solid state.

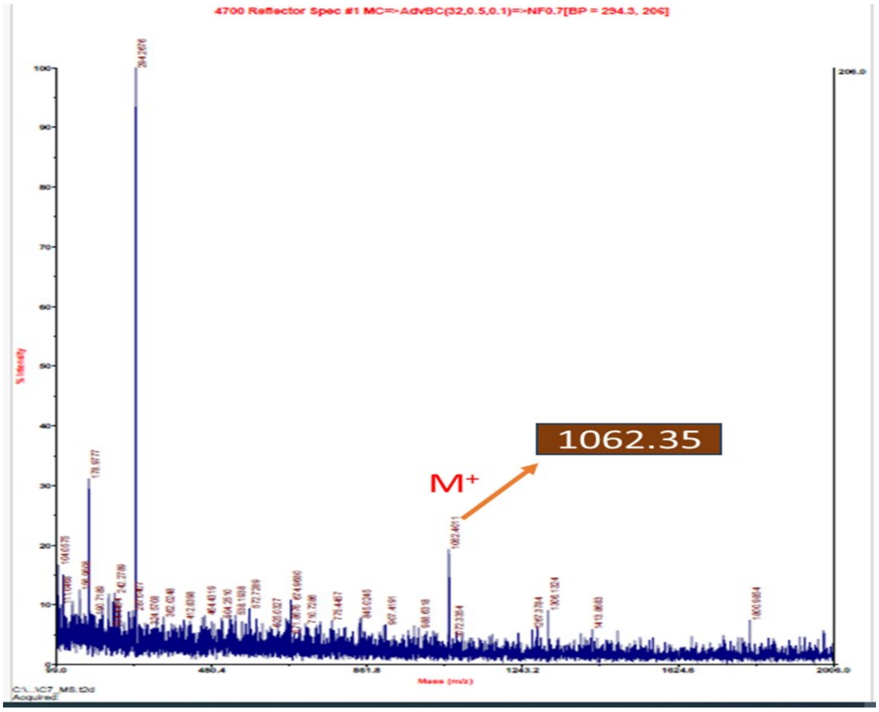

Figure S5c. LCMS of compound (AM-4) recorded in (DMF) solvent.

8. ^{1}H NMR, ^{13}C NMR, IR and MS spectra of (4):

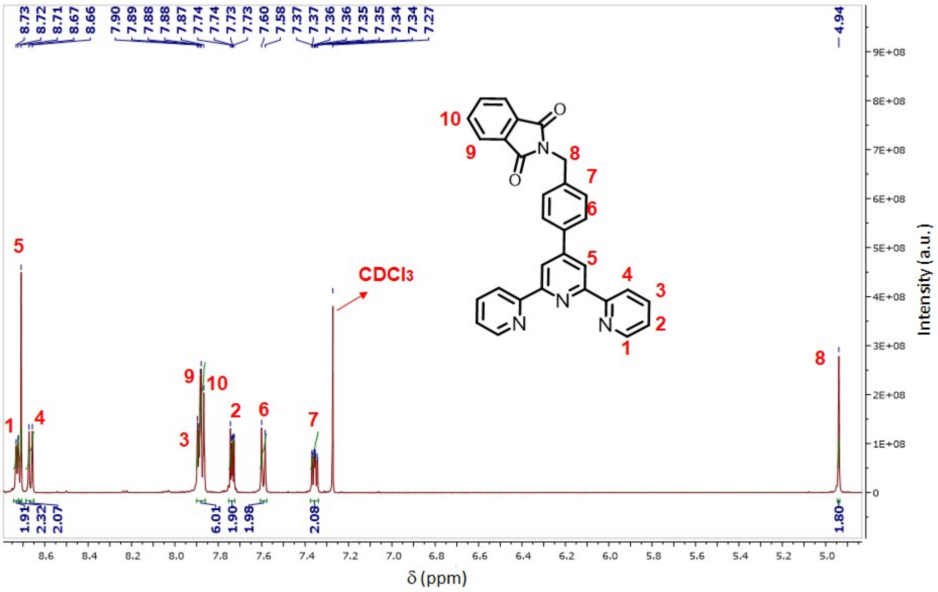

Figure S6a. ^{1}H-NMR (500 MHz, $CDCl_3$, 298K) spectrum for the compound (4) recorded at room temperature.

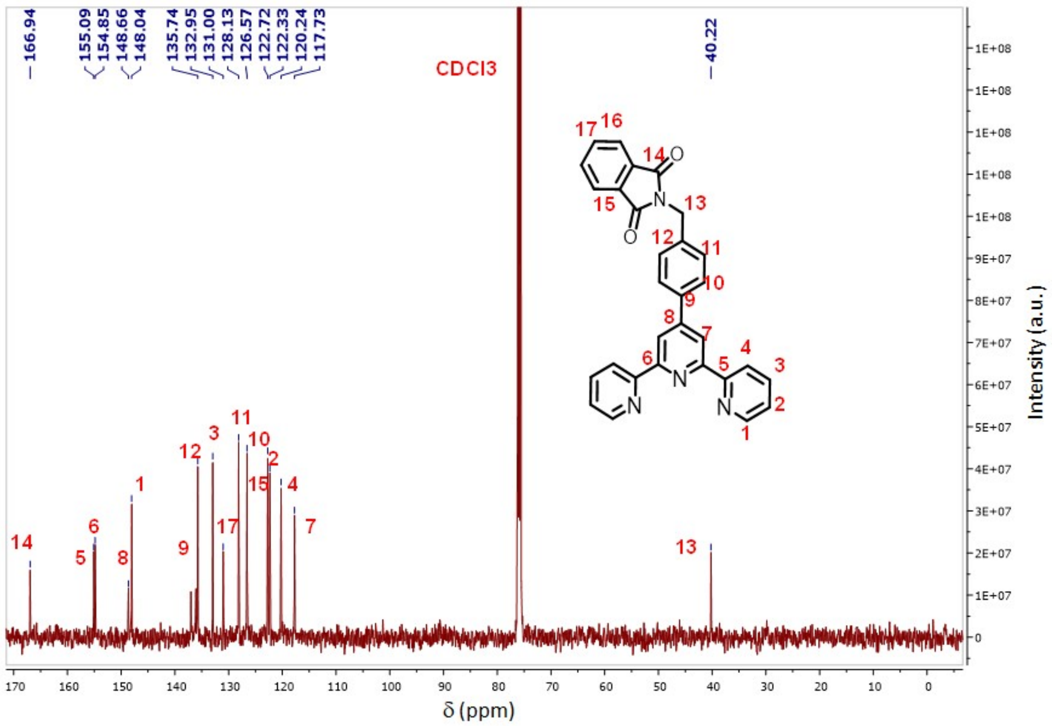

Figure S6b. ^{13}C-NMR (125 MHz, $CDCl_3$, 298K) spectrum for the compound (4) recorded at room temperature.

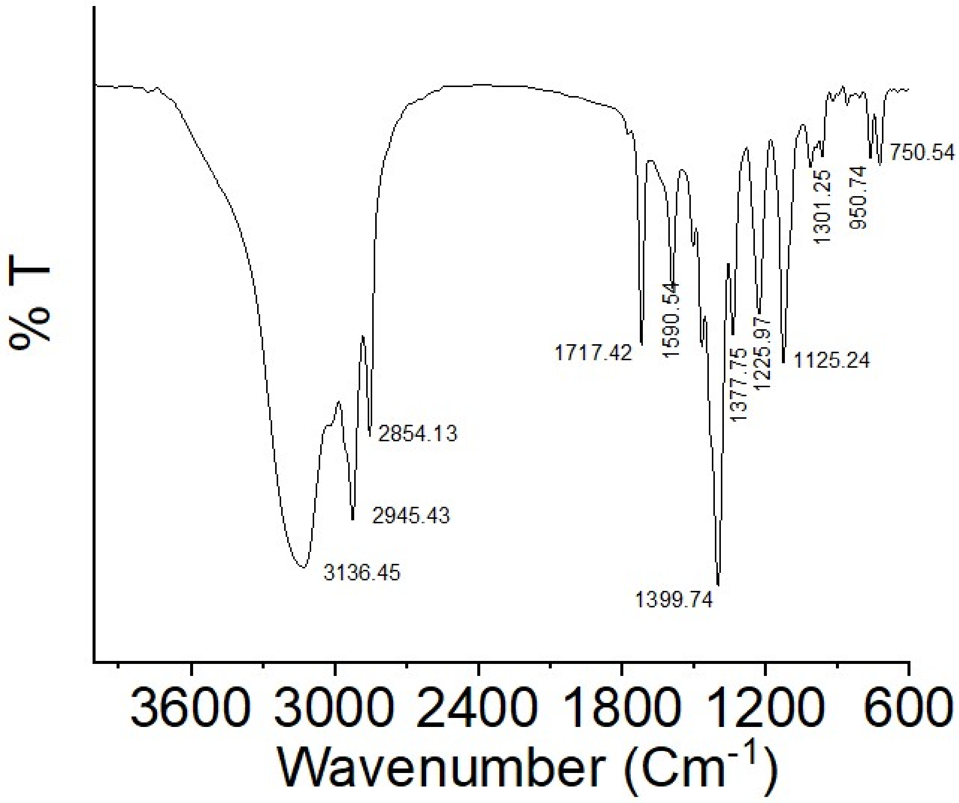

Figure S6c. IR spectrum of compound (4) recorded in solid state.

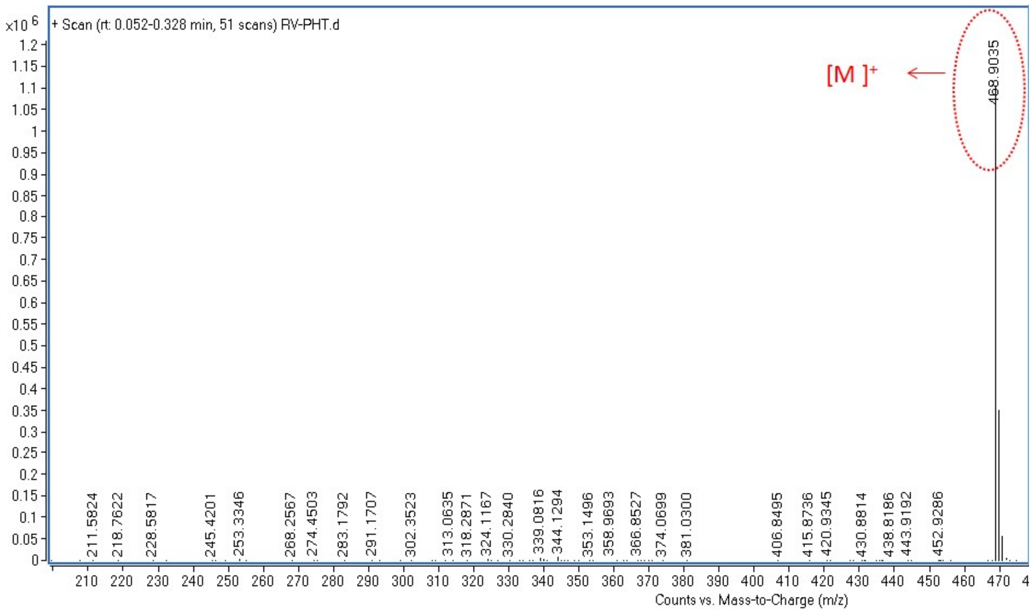

Figure S6d. LCMS of compound (4) recorded in (MeOH) solvent.

9. ^{1}H NMR, ^{13}C NMR, IR and MS spectra of (5):

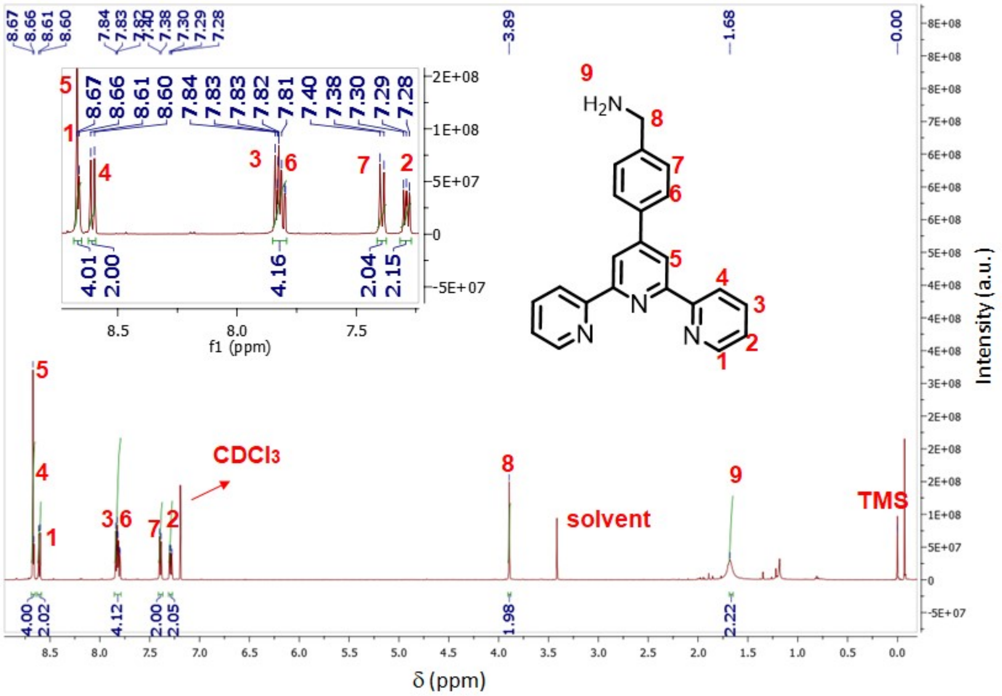

Figure S7a. ^{1}H-NMR (500 MHz, $CDCl_3$, 298K) spectrum for the compound (5) recorded at room temperature.

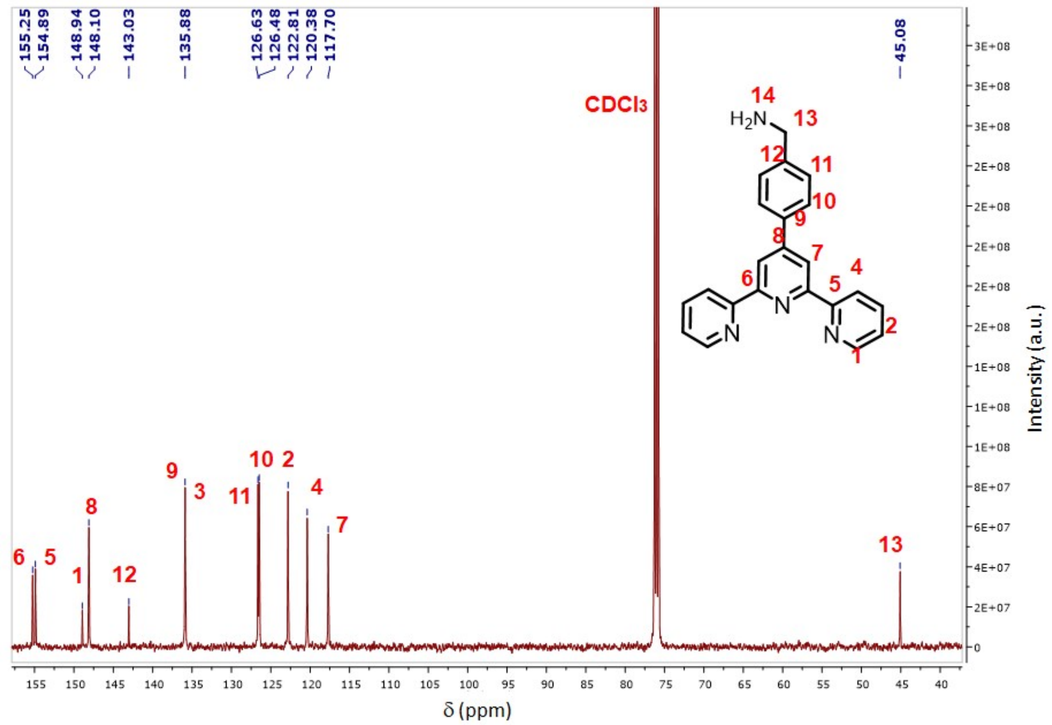

Figure S7b. ^{13}C-NMR (125 MHz, $CDCl_3$, 298K) spectrum for the compound (5) recorded at room temperature.

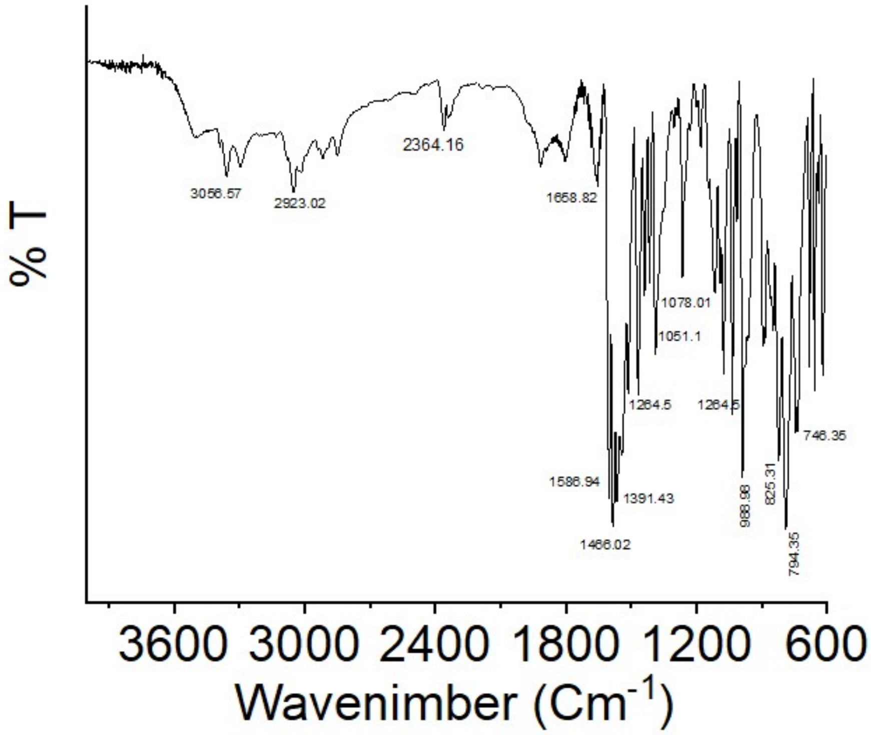

Figure S7c. IR spectrum of compound (5) recorded in solid state.

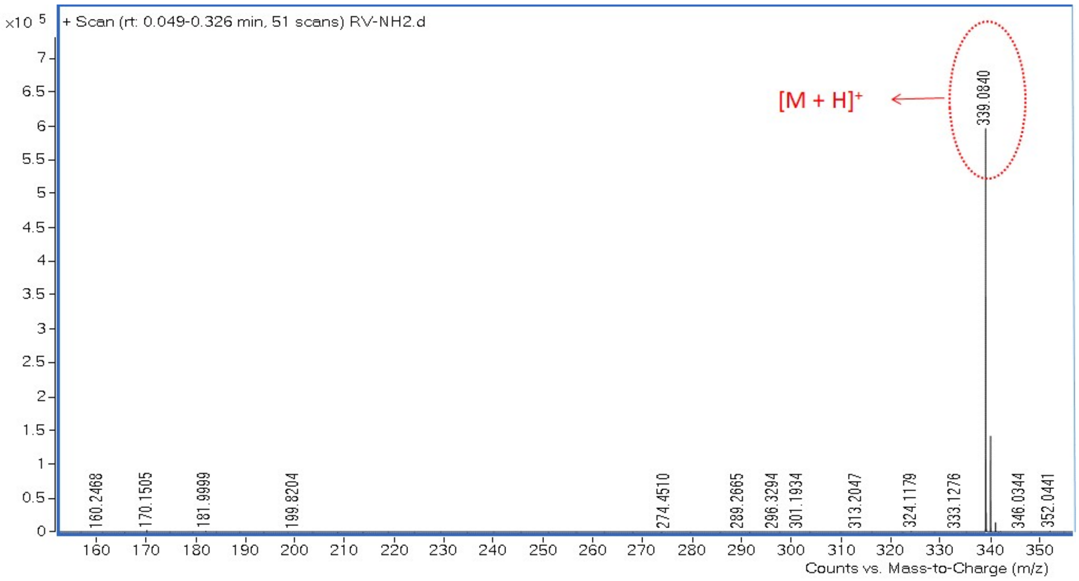

Figure S7d. LCMS of compound (5) recorded in (MeOH) solvent.

10. ^{1}H NMR, ^{13}C NMR, IR and MS spectra of (6):

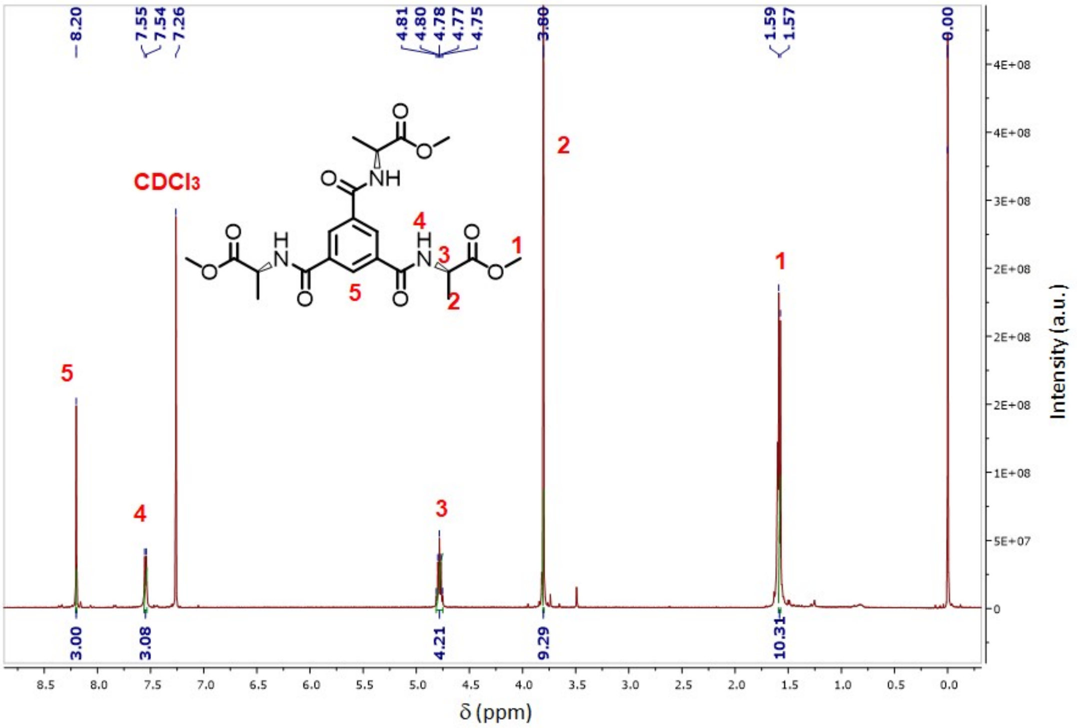

Figure S8a. ^{1}H-NMR (500 MHz, $CDCl_3$ 298K) spectrum for the compound (6) recorded at room temperature.

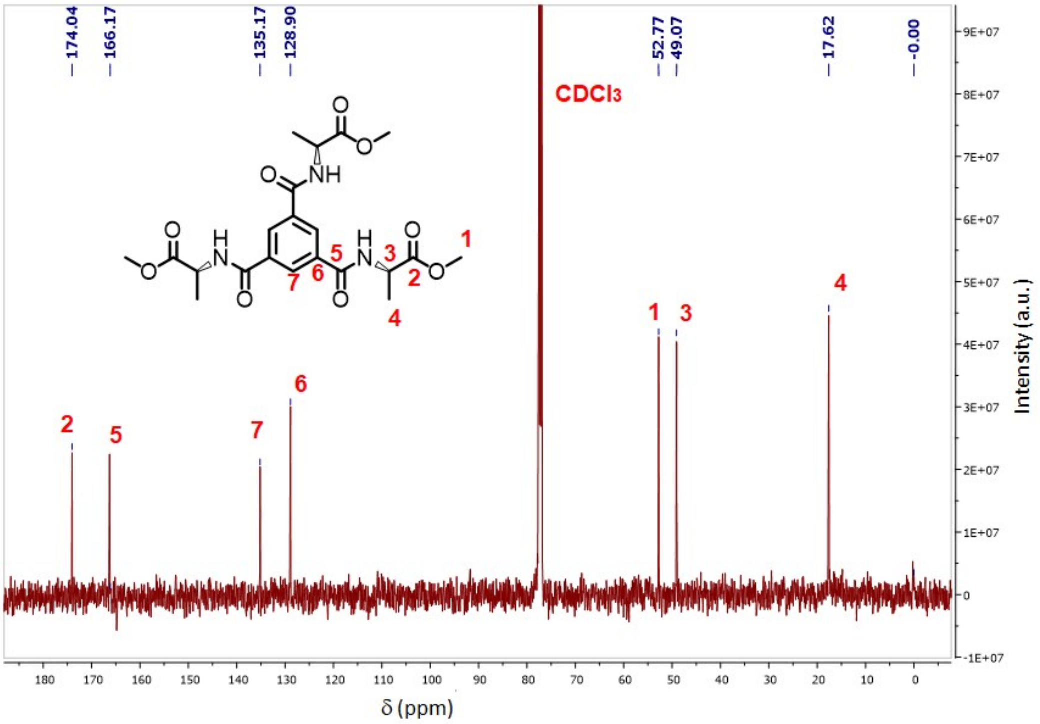

Figure S8b. ^{13}C-NMR (125 MHz, $CDCl_3$, 298K) spectrum for the compound (6) recorded at room temperature.

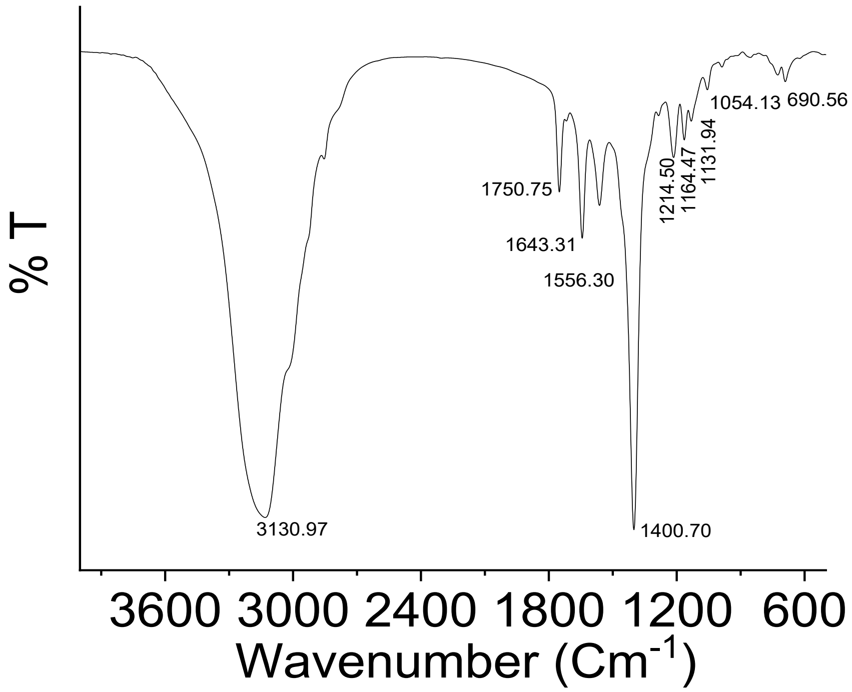

Figure S8c. IR spectrum of compound (6) recorded in solid state.

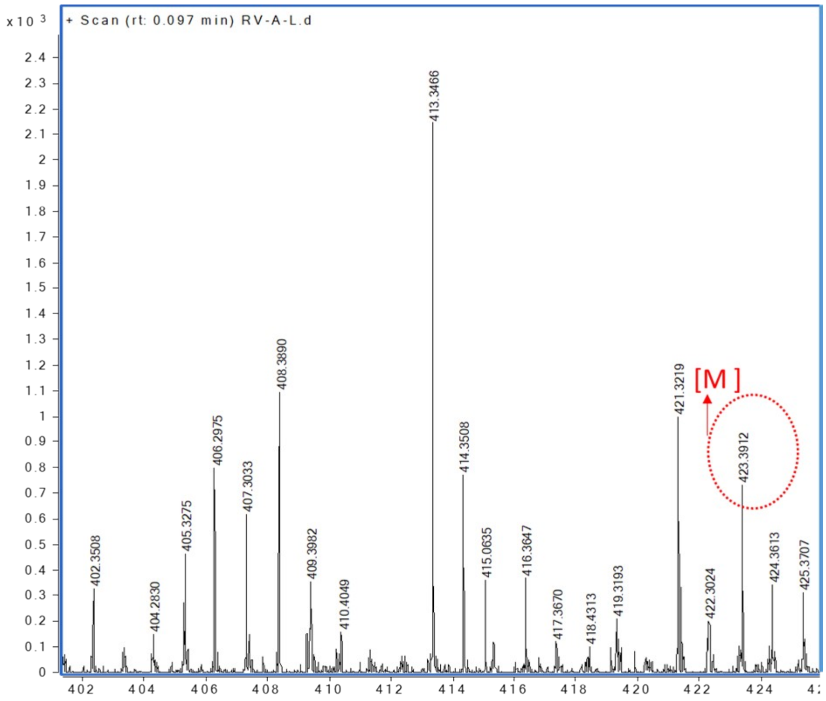

Figure S8d. LCMS of compound (6) recorded in (MeOH) solvent.

11. ¹H NMR, ¹³C NMR, IR and MS spectra of (7):

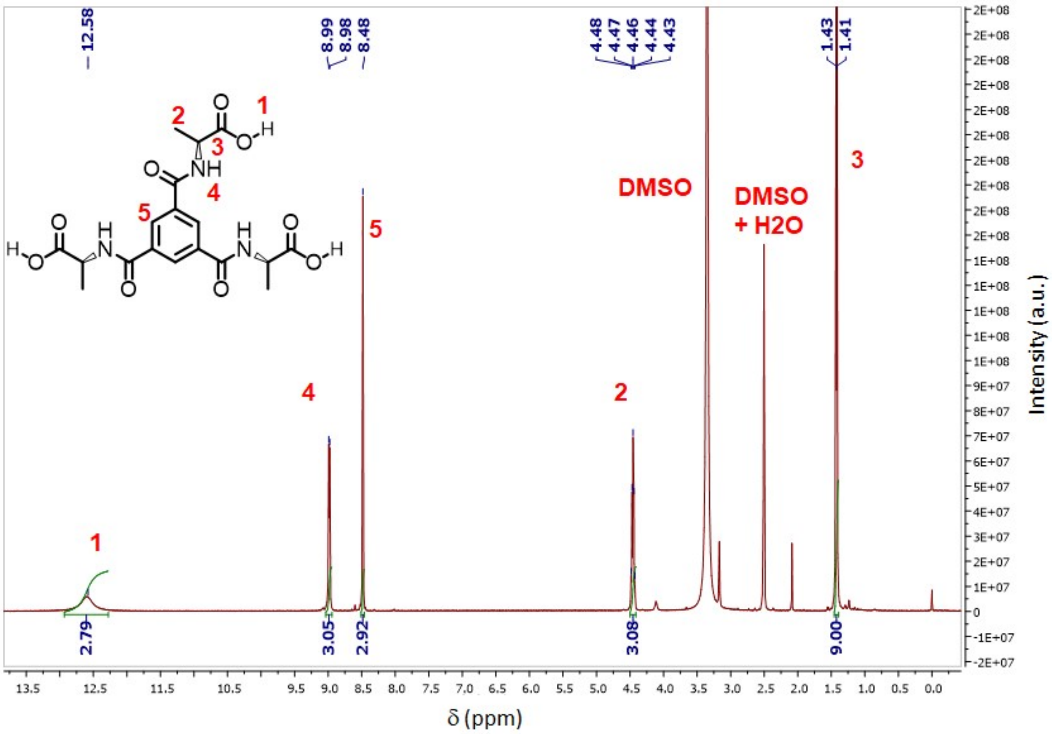

Figure S9a. ¹H-NMR (500 MHz, DMSO-d_6, 298K) spectrum for the compound (7) recorded at room temperature.

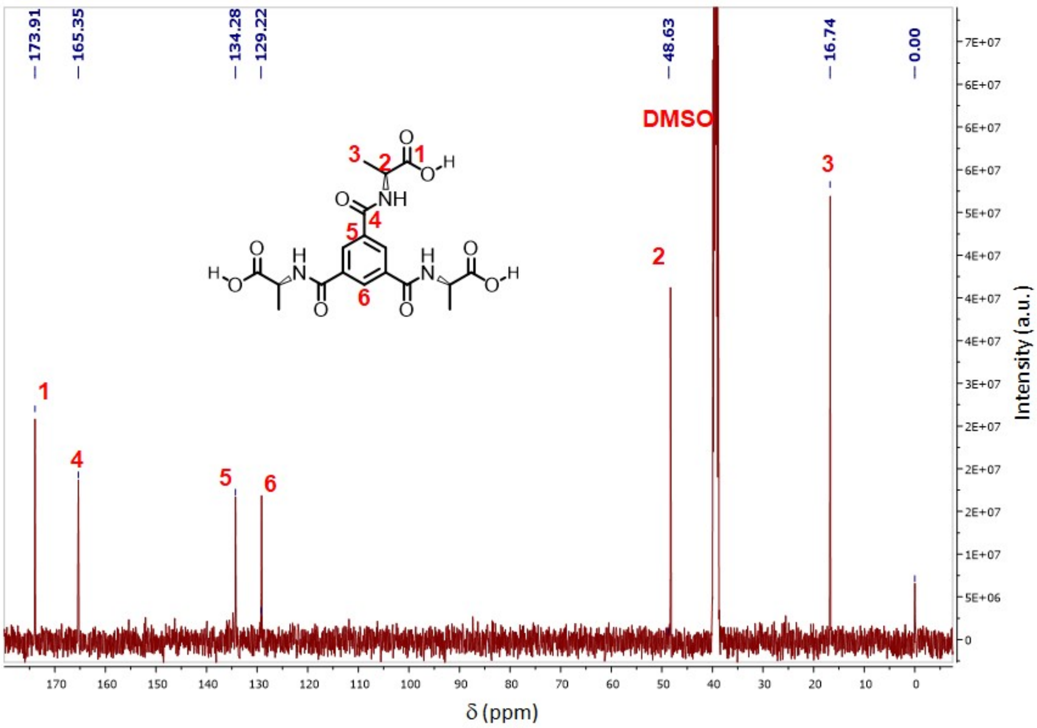

Figure S9b. ^{13}C-NMR (125 MHz DMSO-d_6, 298K) spectrum for the compound (7) recorded at room temperature.

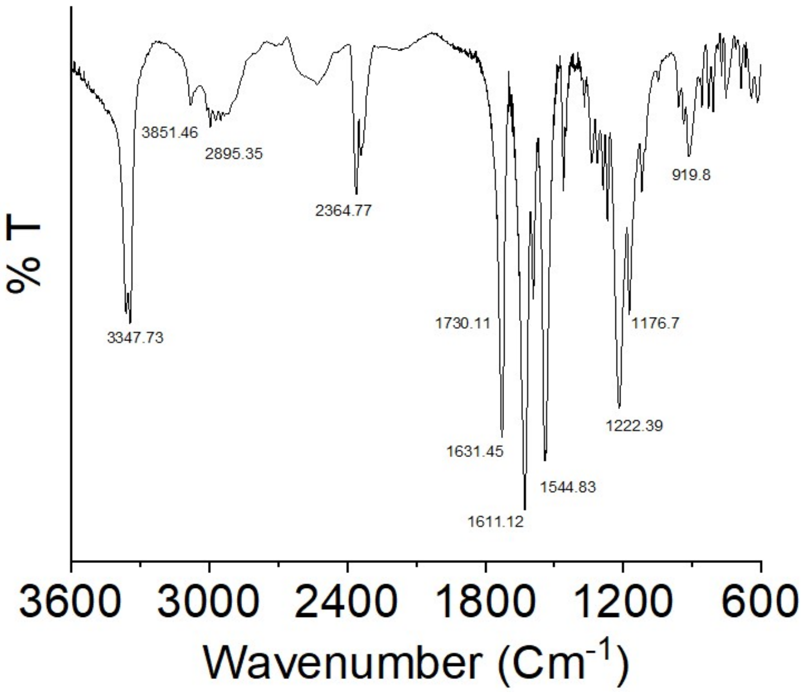

Figure S9c. IR spectrum of compound (7) recorded in solid state.

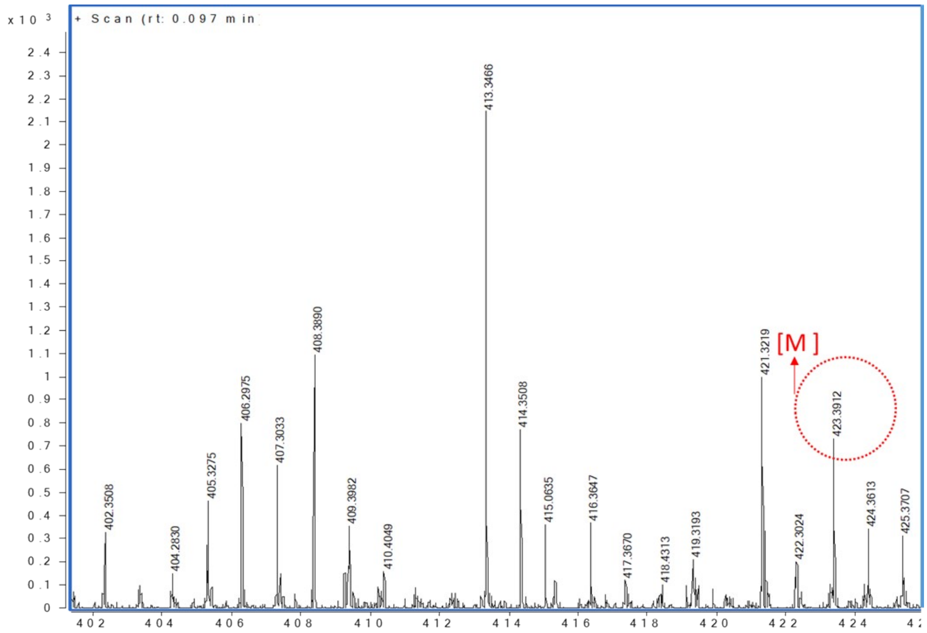

Figure S9d. LCMS of compound (7) recorded in (MeOH) solvent.

12. ^{1}H NMR, ^{13}C NMR, IR and MS spectra of (AM-5):

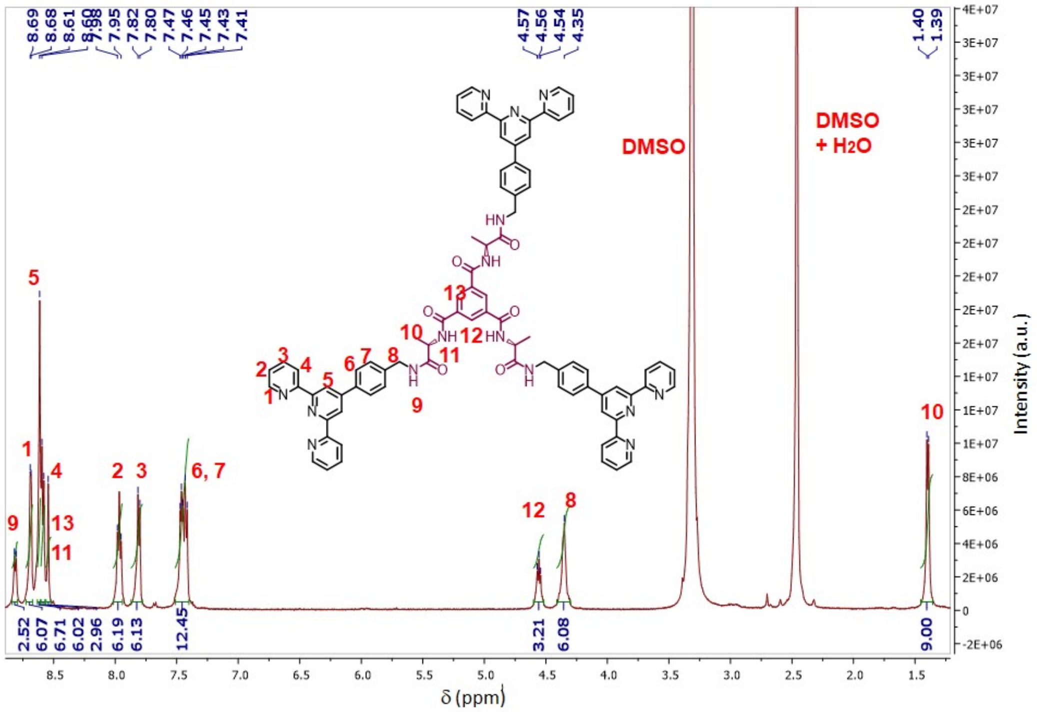

Figure S10a. ^{1}H-NMR (500 MHz, DMSO-d_6, 298K) spectrum for the compound (AM-5) recorded at room temperature.

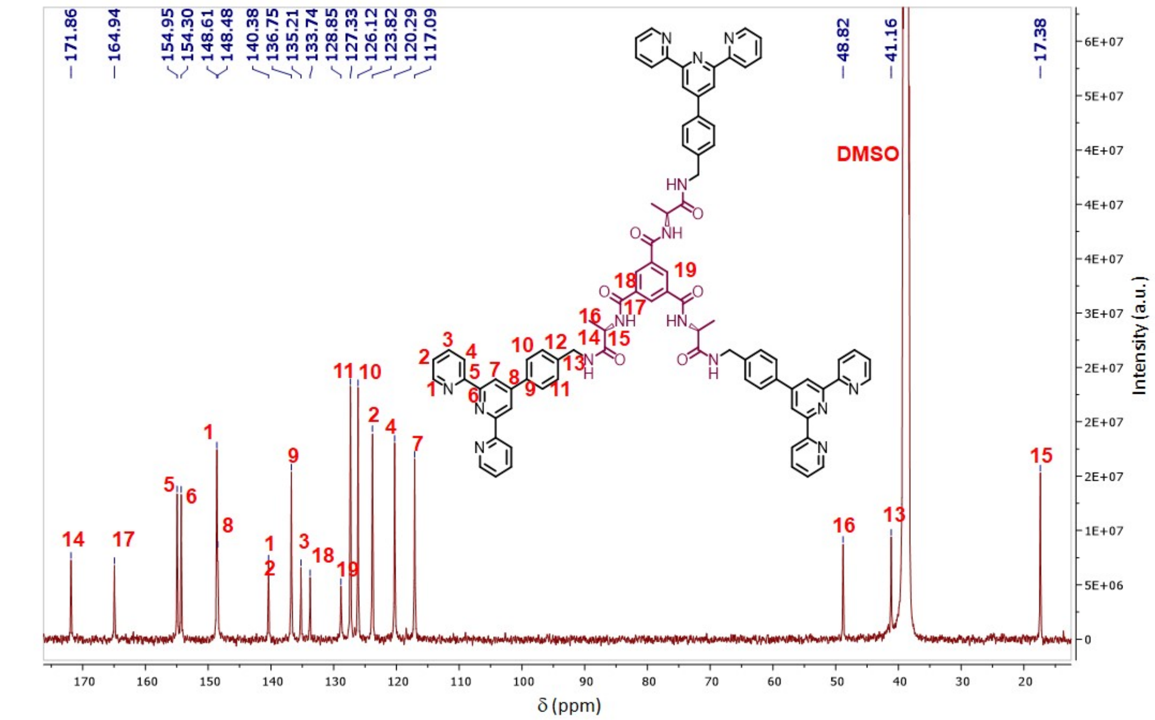

Figure S10b. ^{13}C-NMR (125 MHz, DMSO-d_6, 298K) spectrum for the compound (AM-5) recorded at room temperature.

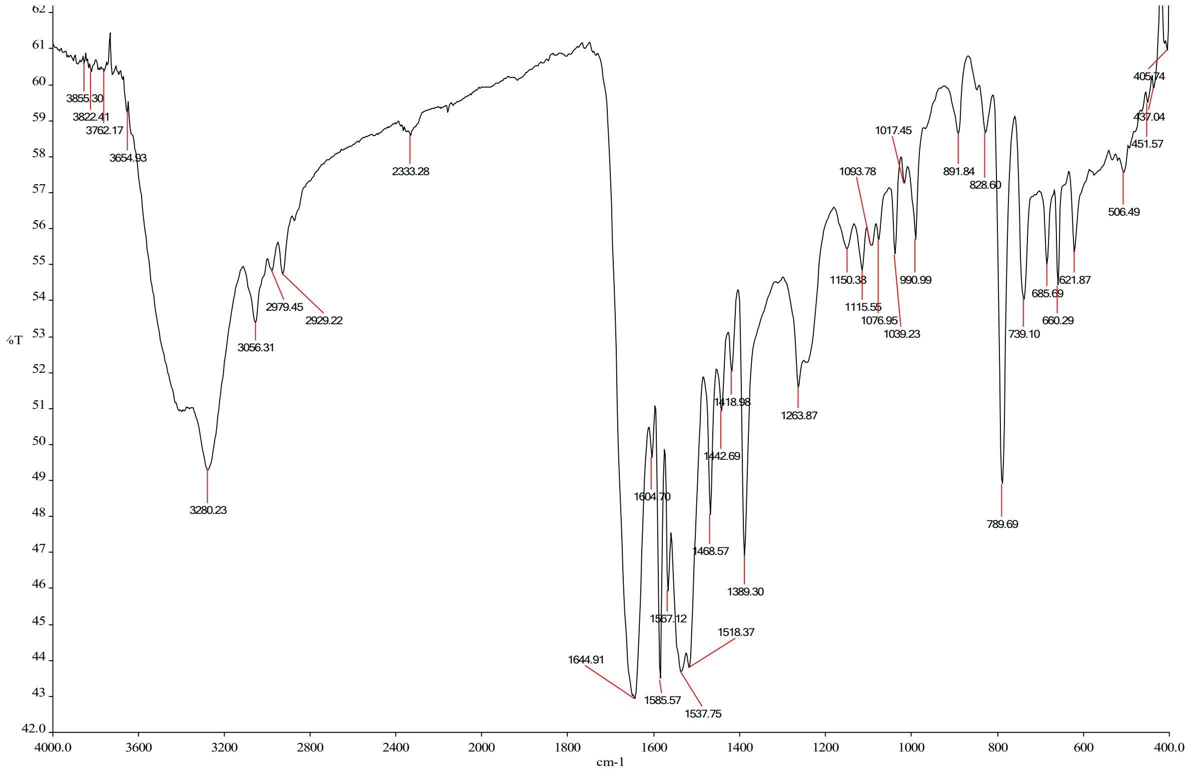

Figure S10c. IR spectrum of compound (AM-5) recorded in solid state.

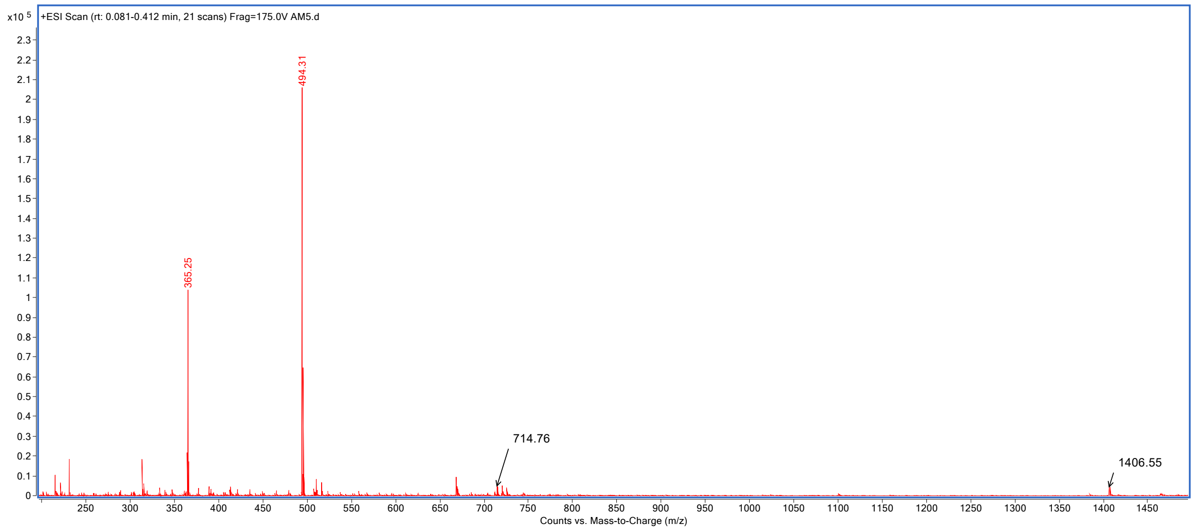

Figure S10d. LCMS of compound (AM-5) recorded in (DMF/MeOH) solvent.

13. ^{1}H NMR, ^{13}C NMR, IR and MS spectra of (AM-6):

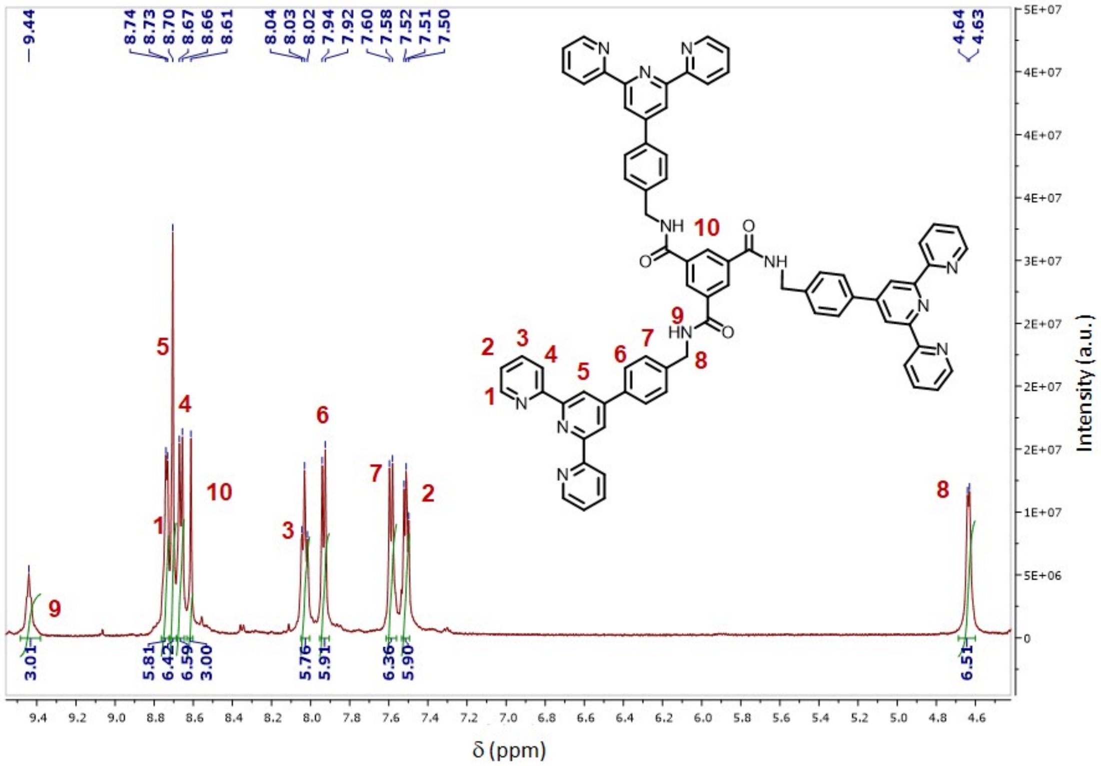

Figure S11a. ^{1}H-NMR (125 MHz, DMSO-d_6, 298K) spectrum for the compound (AM-6) recorded at room temperature.

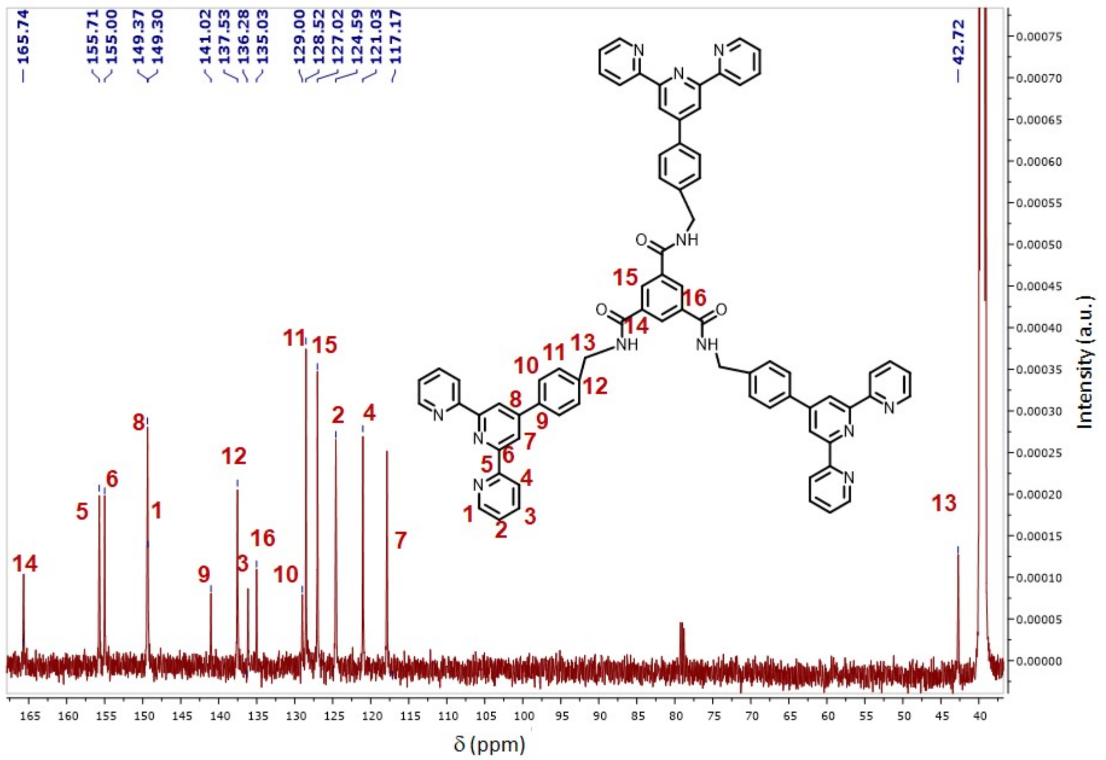

Figure S11b. ^{13}C-NMR (125 MHz, DMSO-d_6, 298K) spectrum for the compound (AM-6) recorded at room temperature.

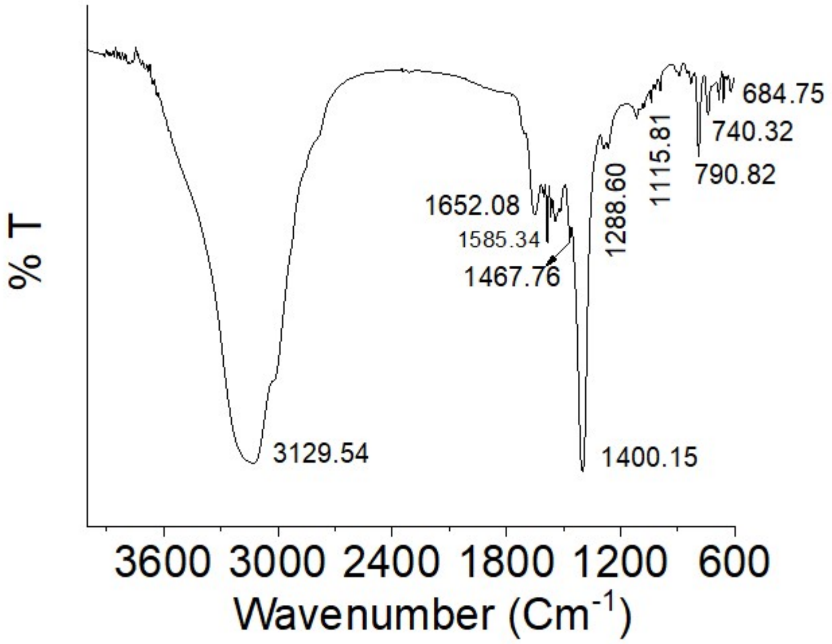

Figure S11c. IR spectrum of compound (AM-6) recorded in solid state.

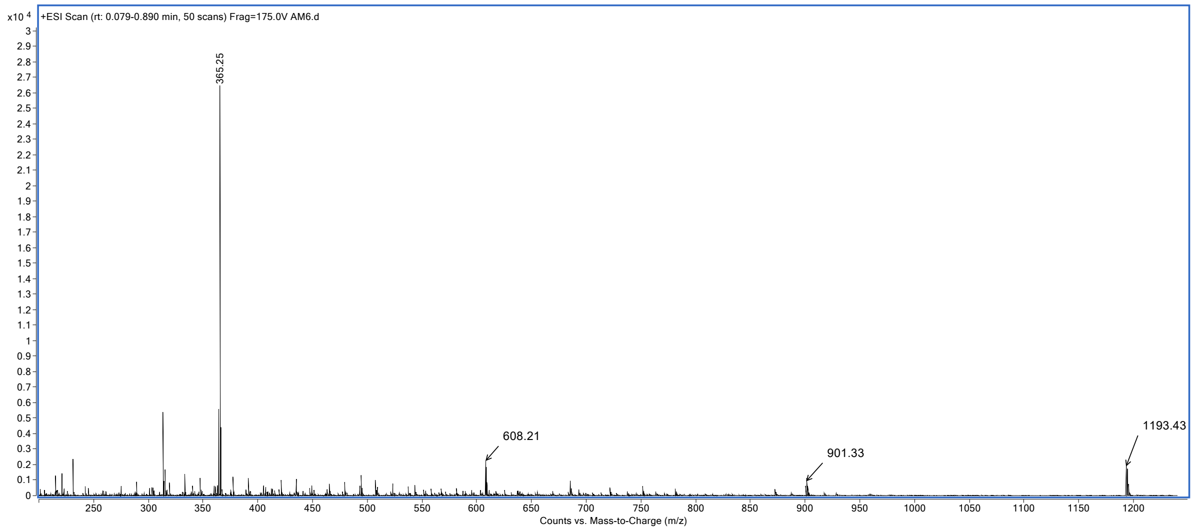

Figure S11d. LCMS of compound (AM-6) recorded in (DMF/MeOH) solvent.

14. UV−vis and fluorescence response:

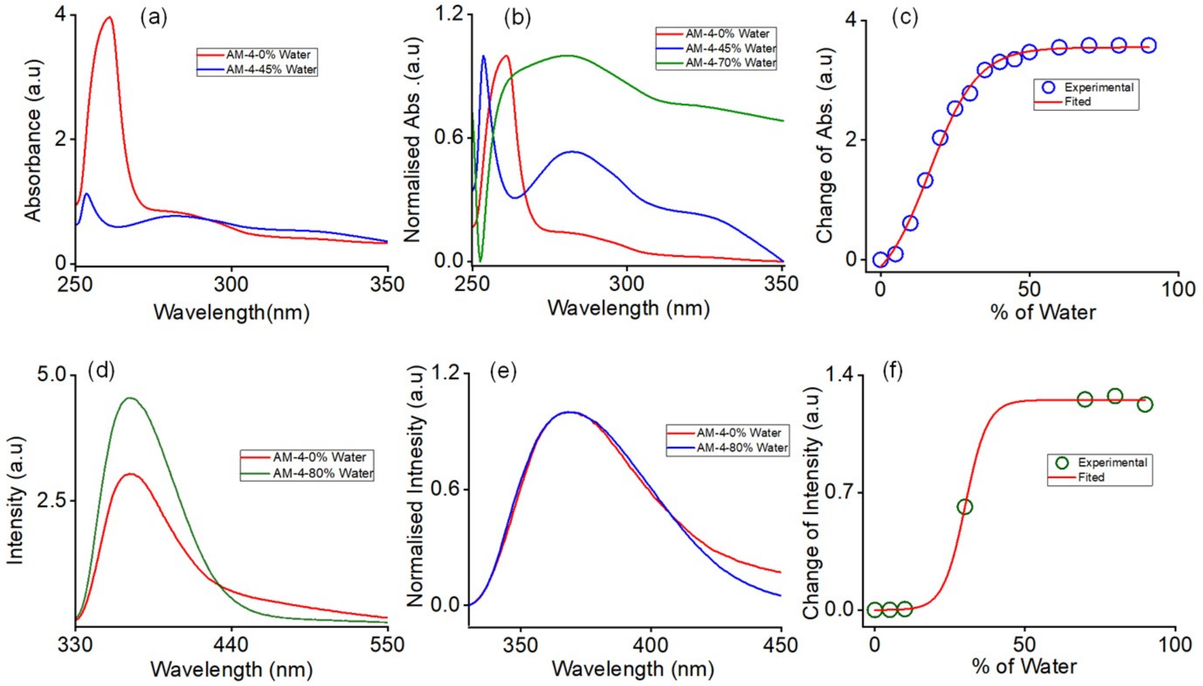

Figure S12. (a) UV-vis absorption spectra of AM-4 in DMF/water (100/0, red line; 55/45, blue line) solvent system. (b) Normalized UV-vis absorption spectra of AM-4 in DMF/water (100/0, red line; 55/45, blue line, and 30/70, green line) solvent system. (c) Corresponding change in absorbance of AM-4 in DMF with varying water contents. (d) Fluorescence spectra of AM-4 in DMF/water (100/0, red line; 20/80, green line) solvent system. (e) Normalized fluorescence spectra of AM-4 in DMF/water (100/0, red line; 20/80, blue line) solvent system. (f) Corresponding change in fluorescence intensity of AM-4 in DMF with varying water contents.

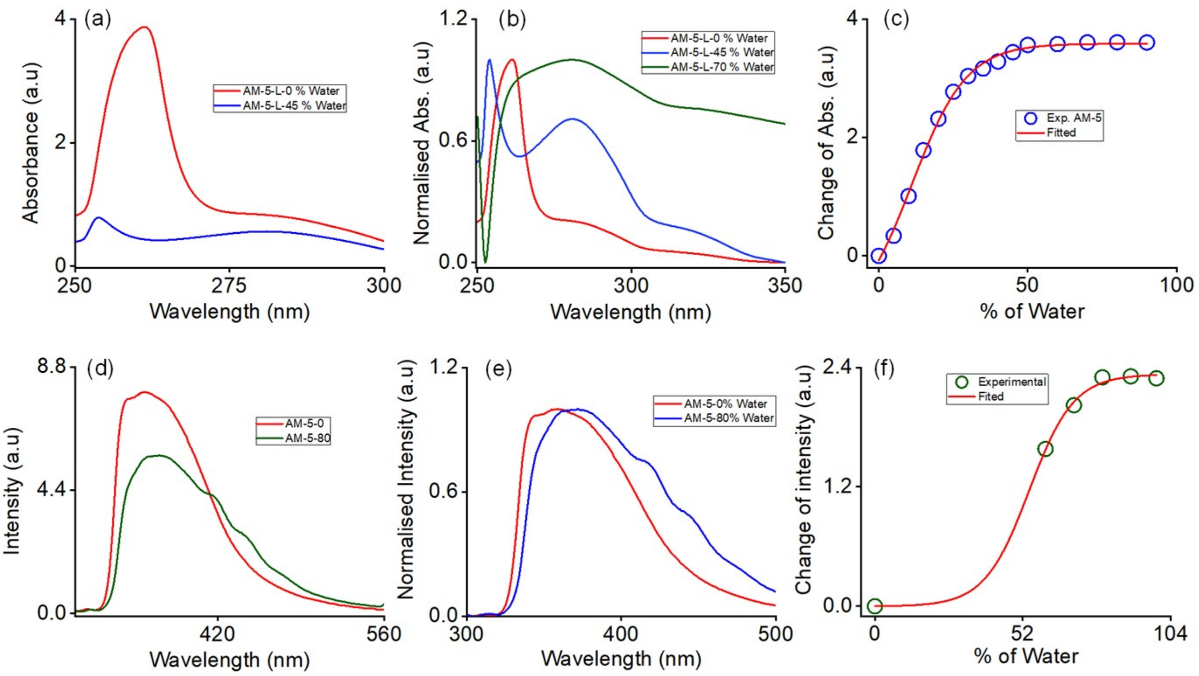

Figure S13. (a) UV-vis absorption spectra of AM-5 in DMF/water (100/0, red line; 55/45, blue line) solvent system. (b) Normalized UV-vis absorption spectra of AM-5 in DMF/water (100/0, red line; 55/45, blue line, and 30/70, green line) solvent system. (c) Corresponding change in absorbance of AM-5 in DMF with varying water contents. (d) Fluorescence spectra of AM-5 in DMF/water (100/0, red line; 20/80, green line) solvent system. (e) Normalized fluorescence spectra of AM-5 in DMF/water (100/0, red line; 20/80, blue line) solvent system. (f) Corresponding change in fluorescence intensity of AM-5 in DMF with varying water contents.

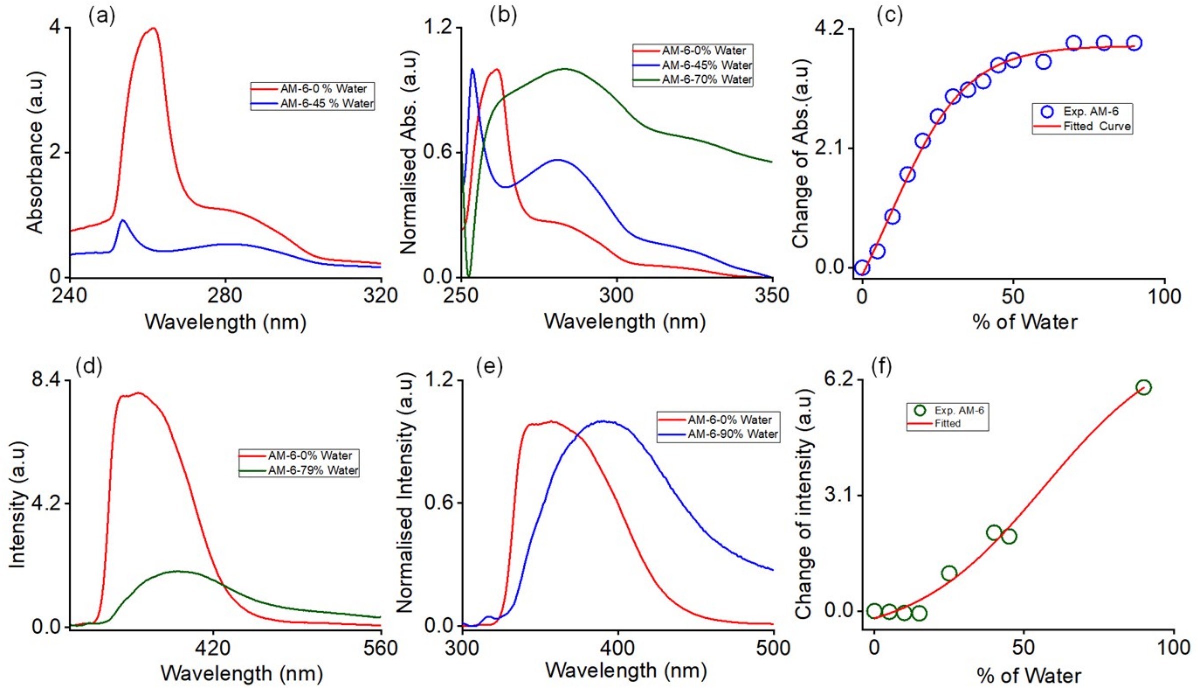

Figure S14. (a) UV-vis absorption spectra of AM-6 in DMF/water (100/0, red line; 55/45, blue line) solvent system. (b) Normalized UV-vis absorption spectra of AM-6 in DMF/water (100/0, red line; 55/45, blue line, and 30/70, green line) solvent system. (c) Corresponding change in absorbance of AM-6 in DMF with varying water contents. (d) Fluorescence spectra of AM-6 in DMF/water (100/0, red line; 20/80, green line) solvent system. (e) Normalized fluorescence spectra of AM-6 in DMF/water (100/0, red line; 20/80, blue line) solvent system. (f) Corresponding change in fluorescence intensity of AM-6 in DMF with varying water contents.

15. Determination of the degree of aggregation (α_{agg}):

The degree of aggregation (α_{agg}) was calculated from the temperature-dependent UV-Vis spectral data by using equation (1) as mentioned below.

$$\alpha\,(T) = \frac{\varepsilon\,(T)\; - \varepsilon_M}{\varepsilon_A - \;\varepsilon_M} \quad \text{.......................................(1)}$$

Where $\varepsilon(T)$ is the measured extinction coefficient at temperature T; ε_M and ε_A are the extinction coefficients of the monomer and fully aggregated state, respectively. The latter two values were determined from the spectral data at high and low temperatures, respectively.

The experimentally determined degree of aggregation was fitted temperature-dependent isodesmic self-assembly model accordingly with equation (2) as mentioned below, to obtain the enthalpy value ΔH.

$$\alpha(T) = \frac{1}{1 + exp[\,-0.908\Delta H\frac{T - T_m}{RT_m^2}]} \quad \text{.................. (2)}$$

Where α is the degree of aggregation, and T_m is the melting temperature defined as the temperature at α = 0.5 at $T = T_m$. R is the Boltzmann constant.

From the experimentally determined degree of aggregation, the number-averaged degree of polymerization, DP_N, calculated temperature-dependent isodesmic self-assembly model accordingly with equation (3), as mentioned below.

$$DP_N(T) = \frac{1}{\sqrt{1 - \alpha(T)}} \text{........................(3)}$$

From the number-averaged degree of polymerization, DP_N, and the known concentration of molecules, c_T, the equilibrium constant K_e was determined as a function of temperature using equation (4), as mentioned below.

$$DP_N(T) = \frac{1}{2} + \frac{1}{2}\sqrt{4K_e(T)C_T + 1} \quad \text{......................... (4)}$$

16. Temperature dependent UV-Vis study:

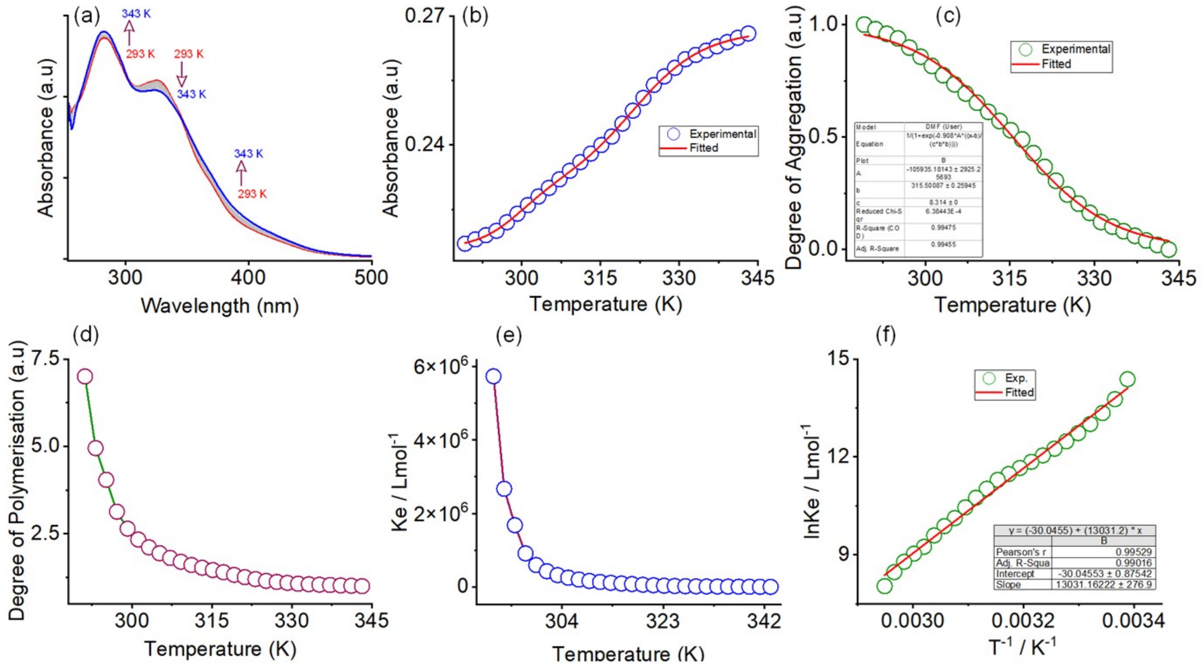

Figure S15. (a) Variable temperature UV-Vis spectra of AM-4 in DMF/water (1:1, v/v). (b) Corresponding plot of change of absorbance with temperature. (c) Temperature-dependent degree of aggregation, calculated from UV-Vis spectral change and corresponding isodesmic fit. (d) Corresponding plot of the degree of polymerization, DP_N, as a function of temperature. (e) Corresponding plot of the equilibrium constant, K_e, as a function of temperature. (f) Corresponding Van't Hoff plot for AM-4.

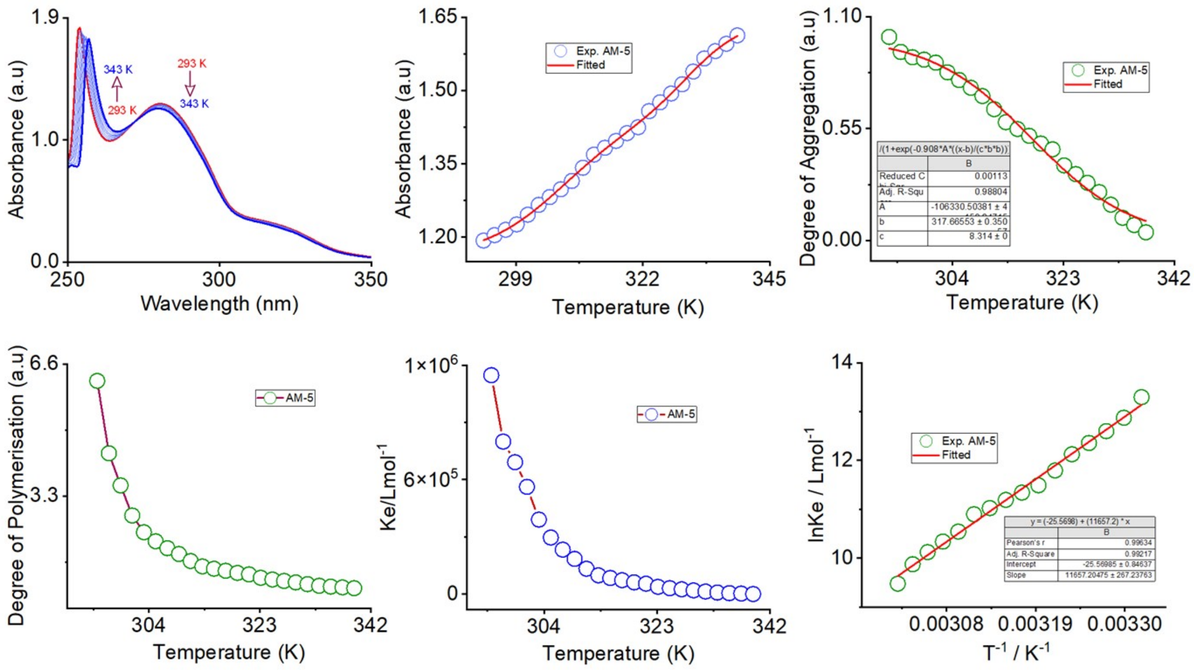

Figure S16. (a) Variable temperature UV-Vis spectra of AM-5 in DMF/water (1:1, v/v). (b) Corresponding plot of change of absorbance with temperature. (c) Temperature-dependent degree of aggregation, calculated from UV-Vis spectral change and corresponding isodesmic fit. (d) Corresponding plot of the degree of polymerization, DP_N, as a function of temperature. (e) Corresponding plot of the equilibrium constant, K_e, as a function of temperature. (f) Corresponding Van't Hoff plot for AM-5.

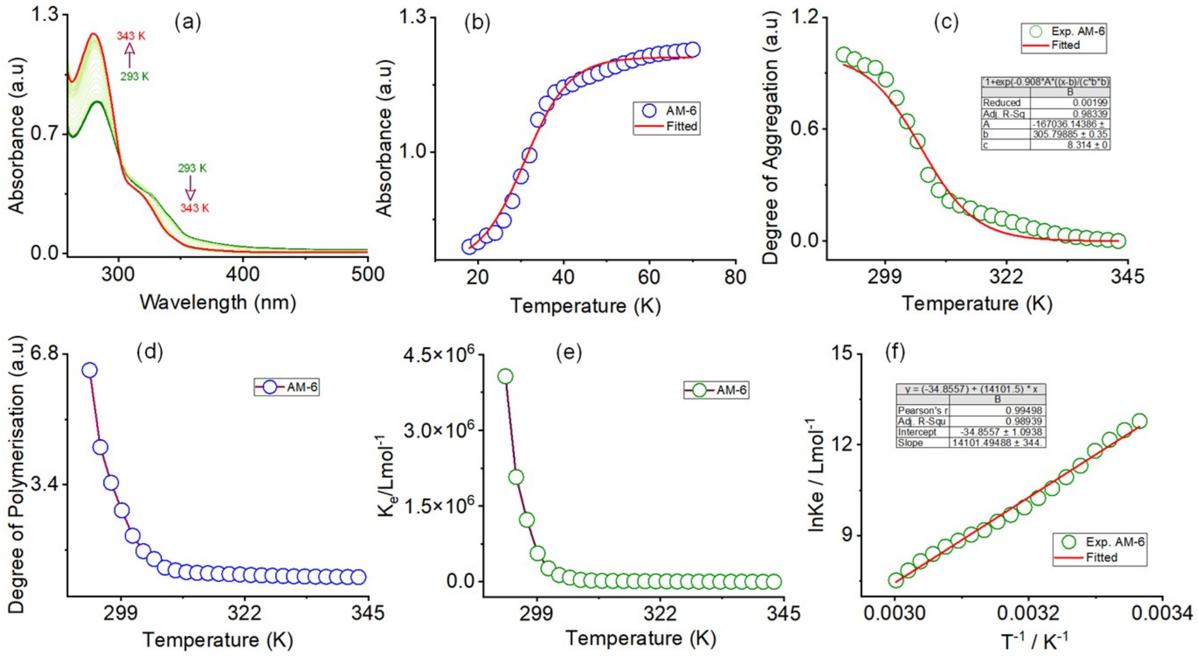

Figure S17. (a) Variable temperature UV-Vis spectra of AM-6 in DMF/water (1:1, v/v). (b) Corresponding plot of change of absorbance with temperature. (c) Temperature-dependent degree of aggregation, calculated from UV-Vis spectral change and corresponding isodesmic fit. (d) Corresponding plot of the degree of polymerization, DP_N, as a function of temperature. (e) Corresponding plot of the equilibrium constant, K_e, as a function of temperature. (f) Corresponding Van't Hoff plot for AM-6.

17. Computationally optimized geometries:

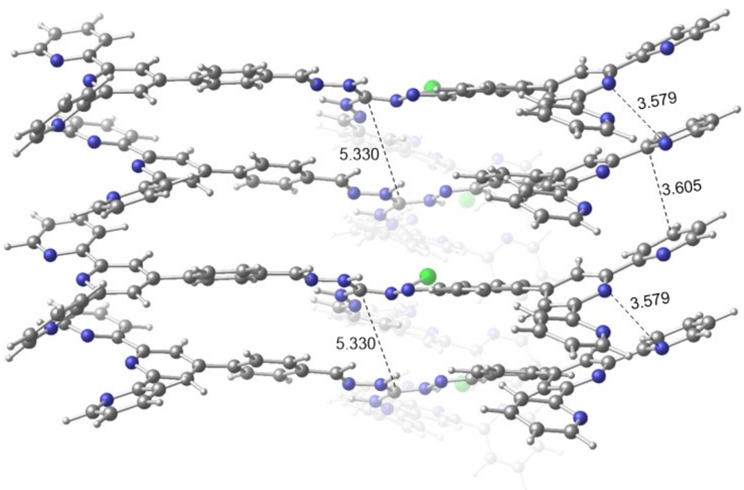

Figure S18. Computationally optimized geometries with possible noncovalent interactions for SONs of AM-4. The unit of bond distances are in Å. [C: grey, N: blue, H: white, Cl: green].

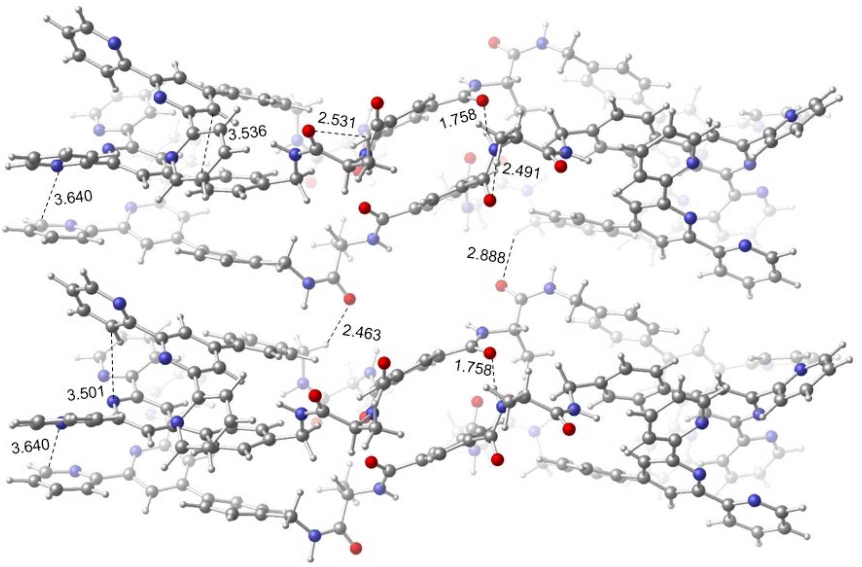

Figure S19. Computationally optimized geometries with possible noncovalent interactions for SONs of AM-5. The unit of bond distances are in Å. [C: grey, N: blue, H: white, O: red].

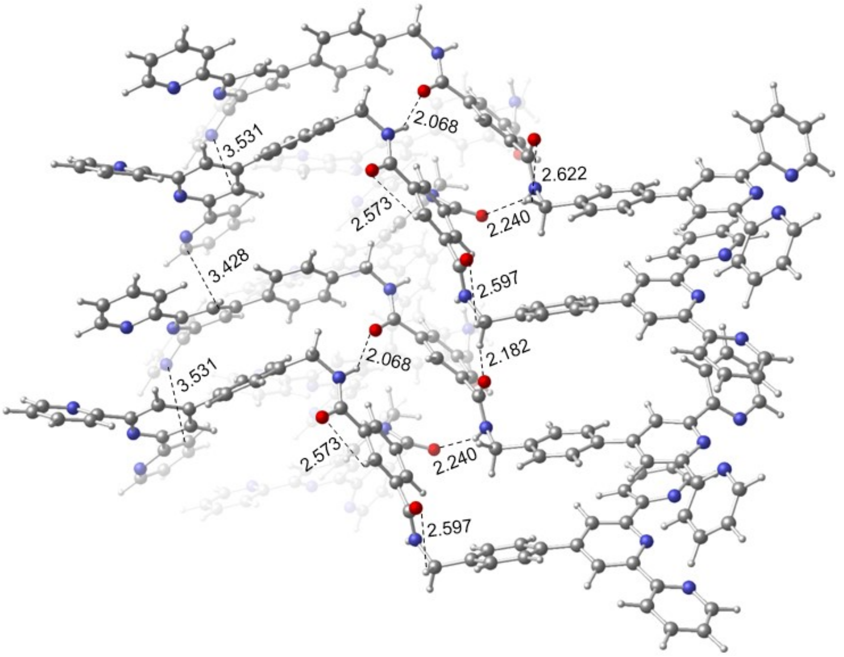

Figure S20. Computationally optimized geometries with possible noncovalent interactions for SONs of AM-6. The unit of bond distances are in Å. [C: grey, N: blue, H: white, O: red].

18. XPS of SONs of AM-4, AM-5 and AM-6:

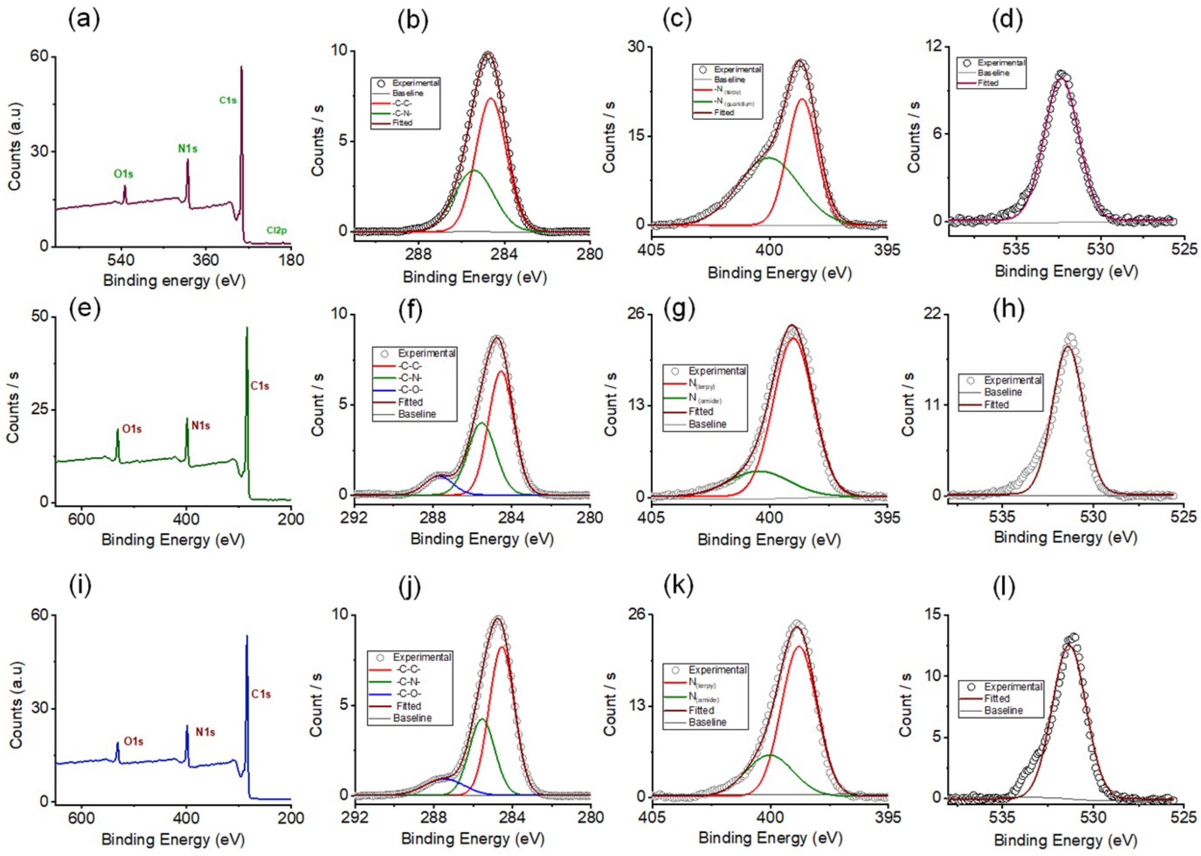

Figure S21: XPS survey spectra of SONs of (a) AM-4, (e) AM-5, and (i) AM-6, respectively. C 1s core-level XPS spectra of (b) AM-4, (f) AM-5, and (j) AM-6, respectively. N1s core level spectra of (c) AM-4, (g) AM-5, and (k) AM-6, respectively. O1s core level spectra of (d) AM-4, (h) AM-5, and (l) AM-6, respectively.

19. TGA profile for SONs of AM-4, AM-5 and AM-6:

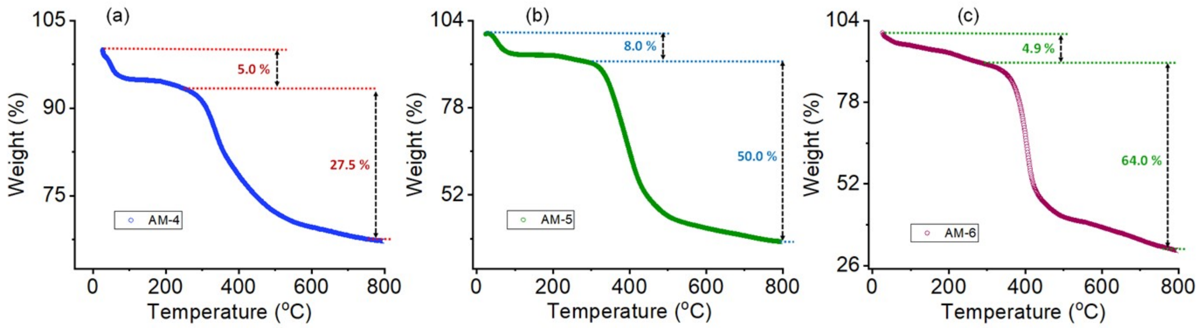

Figure S22: Thermogravimetric analysis (TGA) curves for SONs of (a) AM-4, (b) AM-5 and (c) AM-6, respectively.

20. Mechanical study of the composit films:

Uniaxial tensile tests of all membranes were performed on a texture analyser. All tests were carried out under ambient conditions (temperature 20–25 °C and relative humidity 20–50%) maintained by the building's heating, ventilation and air conditioning system. The Xforce P tensile testing machine with

sensitivity around 2mV/V (Germany, Zwick/Roell) was used to measure mechanical properties to characterise the mechanical strength of the as-prepared membrane by using two methods (1 and 2), wherein elongation and resulting load were recorded. The tests ended when the specimens fractured. The Universal Testing Machine Xforce P by Zwick/Roell was equipped with a force measuring range of up to 500N.

We hahve prepared the composite films of SONs assemblies to study the mechanical property through the following methods. First, a solution of all three molecules (AM-4, AM-5, and AM-6) in DMF (10 mg/mL) was exposed to 1 mL of deionized water. The solution was mixed thoroughly and left undisturbed for about 10 minutes to complete the automated self-assembly process. This SONs dispersion was added to a stirred solution of 10 ml of (1:1 DMF/water, (50 mg/ml) polyethylene oxide (PEO). The homogeneous viscous solution was cast on a petri dish and dried in the casting chamber for 24 hrs to obtain composite films. The dimensions of the as-prepared thin film were 4 cm (length) and 1 cm (width). The thickness of the films were 108.0 μm (AM-4) 110.0 μm (AM-5), 113.0 μm (AM-6), and 109.0 μm (PEO film), respectively. The elongation rate was 20 mm/min.

Table S1: Mechanical properties of the SONs composit film of AM-4, AM-5, AM-6 and PEO.

Sample Name	Upper yield point (MPa)	Young's Modulus (E) (MPa)	Modulus of Resilience (M_R) (kJ/m³)	Ultimate tensile strength ($\sigma_{ult.}$) (MPa)	Fracture strength (MPa)	Fracture strain / ductility (ε_f) (%)	Modulus of Toughness (M_T) (kJ/m³)
AM-4	6.57 ± 0.11	770.8 ± 32	255.5 ± 13	8.9 ± 0.8	6.2 ± 0.08	79.2 ± 4	6321 ± 324
AM-5	2.80 ± 0.07	320.3 ± 23	207.7 ± 19	4.3 ± 0.05	2.8 ± 0.06	79.4 ± 5	2554 ± 183
AM-6	18.18 ± 0.14	1050.5 ± 38	1196.2 ± 36	22.7 ± 0.12	16.3 ± 0.11	79.3 ± 4	15666 ± 423
PEO	1.81 ± 0.04	384.3 ± 26	58.9 ± 6	3.8 ± 0.04	2.3 ± 0.04	79.2 ± 3	2736 ± 195

21. Sample preparation for Li-ion conductivity:

The samples for the Li-ion conductivity measurement are prepared as follows. A solution of all three molecules (AM-4, AM-5, and AM-6) in DMF (500 μL, 1.0 mg/mL) was exposed to 500 μL of deionized water containing 1, 2, 3 and 6 equivalents of $LiClO_4$ at 298 K. The solution was mixed thoroughly and left undisturbed for about 10 min to complete the automated self-assembly process. An aliquot of these suspensions was drop-casted on the surface of a Whatman filter membrane separator and remove the residue solvent. The prepared samples were allowed to dry at 50 °C under vaccume and sandwiched in between the SS electrode. Then we proceed to measure the conductivity.

22. Details of conductivity measurements:

The conductivity study has been done using the electrochemical work station (VSP Biologic, France). Ionic conductivity study was carried out through stainless steel electrode. The radius of the SS electrode was 7 mm and the area was calculated as 1.5386 cm². Whatman filter paper was used as a separator and the thickness of the separator was measured by screw gauge method. The thickness of the separator was 0.095 cm. The prepared samples are drop casted over the Whatman separator surface and the separator

was allowed to dry at 50 ^{o}C under vacuumed. After that, the sample was subjected to *ac* impedance study. Finally, the conductivity of the desired samples was calculated via the known standard equation: $\sigma = d/RA$. Where, d = Thickness (cm), R = Resistance (ohm) and A = Area (cm^2).

23. Conductivity data:

Table S2: Resistance and lithium ionic conductivity values for SONs of AM-4 with varying $LiClO_4$ concentration at room temperature.

Sample ID	Equivalents of $LiClO_4$	Area (A) (cm^2)	Thickness (d) (cm)	Resistance (R) (ohm)	Conductivity (σ) (S cm^{-1})
AM-4-Li-1	1	1.5386	0.095	456.45	1.352×10^{-4}
AM-4-Li-2	2	1.5386	0.095	380.75	1.621×10^{-4}
AM-4-Li-3	3	1.5386	0.095	360.69	1.711×10^{-4}
AM-4-Li-6	6	1.5386	0.095	261.28	2.363×10^{-4}

Table S3: Resistance and lithium ionic conductivity values for SONs of AM-5 with varying $LiClO_4$ concentration at room temperature.

Sample ID	Equivalents of $LiClO_4$	Area (A) (cm^2)	Thickness (d) (cm)	Resistance (R) (ohm)	Conductivity (σ) (S cm^{-1})
AM-5-Li-1	1	1.5386	0.095	667.23	9.253×10^{-5}
AM-5-Li-2	2	1.5386	0.095	453.37	1.361×10^{-4}
AM-5-Li-3	3	1.5386	0.095	425.44	1.451×10^{-4}
AM-5-Li-6	6	1.5386	0.095	209.98	2.941×10^{-4}

Table S4: Resistance and lithium ionic conductivity values for SONs of AM-6 with varying $LiClO_4$ concentration at room temperature.

Sample ID	Equivalents of $LiClO_4$	Area (A) (cm^2)	Thickness (d) (cm)	Resistance (R) (ohm)	Conductivity (σ) (S cm^{-1})
AM-6-Li-1	1	1.5386	0.095	593.02	1.041×10^{-4}
AM-6-Li-2	2	1.5386	0.095	475.14	1.299×10^{-4}
AM-6-Li-3	3	1.5386	0.095	354.42	1.742×10^{-4}
AM-6-Li-6	6	1.5386	0.095	197.90	3.121×10^{-4}

24. Comparable table of Li-ion Conductivity Data:

Table S5: Comparable chart for Lithium-ion conductivity values of reported materials.

Materials	Li-ion Conductivity (S/cm^{-1})	Description	Ref.
Organic nanorod	3.12×10^{-4} at 298 K	Self-assembled organic nanorods	This work
organic nanosheets	3.42×10^{-4} at 298 K	Self-assembled cationic organic nanosheets	Chem. Sci., 2021, 12, 13878
Organic Nanotubes	3.91×10^{-5} at 298 K	Self-Assembled Organic Nanotubes	J. Am. Chem. Soc. 2021, 143, 17655−17665
Zwitterionic Nano sheets	5.1×10^{-5} at 298 K	Self-assembled zwitterionic organic nanosheets	ACS App. Mater. & Inter., 2020, 12, 58122-58131.
Supramolecular polymer	1.2×10^{-4} S cm^{-1} at 298 K.	Supramolecular polymer electrolytes.	Nat. Commun. 2019, 10, 5384.
Solid polymer electrolyte	2.5×10^{-5} at 298 K.	Solid polymer electrolyte.	Angew. Chem. Int. Ed. 2014, 53, 3631 –3635
Polymerized ionic networks.	5.32×10^{-3} At 295 K.	Polymerized ionic networks.	Adv. Mater. 2015, 27, 8088-8094.
Self-assembled Nano sheets	2.4×10^{-7} at 298 K	Self-assembled nanosheets from positively charged polycyclic aromatic hydrocarbons	Angew. Chem. 2011, 123, 2843 −2846
MOF	5.5×10^{-5} at 300 K	Mg_2(dobdc)·0.35LiOiPr·0.25$LiBF_4$· EC·DEC (EC = ethylene carbonate; DEC = diethyl carbonate). MOF + liquid organic molecule (on the film)	J. Am. Chem. Soc. 2011, 133, 14522.
MOF	1.8×10^{-5} at 293 K.	LiOtBu-grafted UiO-66 (MOF+ Post-surface Modification)	Chem. Eur. J. 2013, 19, 5533.
MOF	10^{-6} at 295K	MOF (10wt%)+LiTFSI+PEO (MOF-loaded polymer Electrolytes)	J. Mater. Chem. A 2014, 2, 9948.
COF	6.04×10^{-6} at 313K	Polyelectrolyte Covalent Organic Frameworks	J. Am. Chem. Soc. 2018, 140, 7429–7432
COF	2.7×10^{-5} S cm^{-1} at 298 K	Lithium sulfonated COF	J. Am. Chem. Soc. 2019, 141, 5880−5885
COF	3.05×10^{-5} at 298 K	Ionic COF with spiroborate Linkage	Angew. Chem. Int. Ed. 2016, 55, 1737 –1741
COF	7.2×10^{-3} at 298 K	Lithium Imidazolate COF	J. Am. Chem. Soc. 2019, 141, 7518−7525
COF	5.74×10^{-5} at 300 K	Guanidium based cationic COF	J. Am. Chem. Soc. 2018, 140, 896−899

25. Calculation of Li-ion transference number (t_{Li+}):

We employed the Bruce-Vincent method to calculate the Li-ion transference number, using the symmetric cell Li/AMLi/Li and allowing the DC current to flow through the cell. The potentiostatic polarisation study's steady state current and the associated spectral curve are shown below in Figures S23a (Li/AM-4Li/Li), S24a (Li/AM-5Li/Li), and S25a (Li/AM-6Li/Li), respectively.[1] Here, it is also evidenced that the electrochemical stability of material with the less degradation observed from the stability curves. The cell's impedance was examined both before and after the potentiostatic polarization investigation. The measured impedance curves for Li/AM-4Li/Li, Li/AM-5Li/Li, and Li/AM-6Li/Li are

shown in Figures S23b, S24b, and S25b, respectively. The required values needed for the calculation of transference number (t_{Li+}) were tabulated in Table S6. The Li-ion transference number was calculated by the following equation as:

$$t_{\mathrm{Li}^+} = \frac{I_{\mathrm{ss}}(\Delta V - I_0 R_0)}{I_0(\Delta V - I_{\mathrm{ss}} R_{\mathrm{ss}})}$$

Where, I_{ss} is the steady state current, ΔV is the applied potential, I_0 is the initial current, R_0 and R_{SS} are the interfacial resistance before and after potentiosatatic polarization study respectively.

Table S6. The observed values for t_{Li+} calculation measured through potentiostatic polarization method.

Sl.No	SONs	I_{ss} (mA)	ΔV (V)	I_0 (mA)	R_0 (Ω)	R_{SS} (Ω)	t_{Li+}
1	AM-4	0.80	0.05	0.73	35.48	41.1	0.8
2	AM-5	1.05	0.05	0.55	24.76	56.8	0.4
3	AM-6	0.90	0.05	1.03	27.54	33.6	0.8

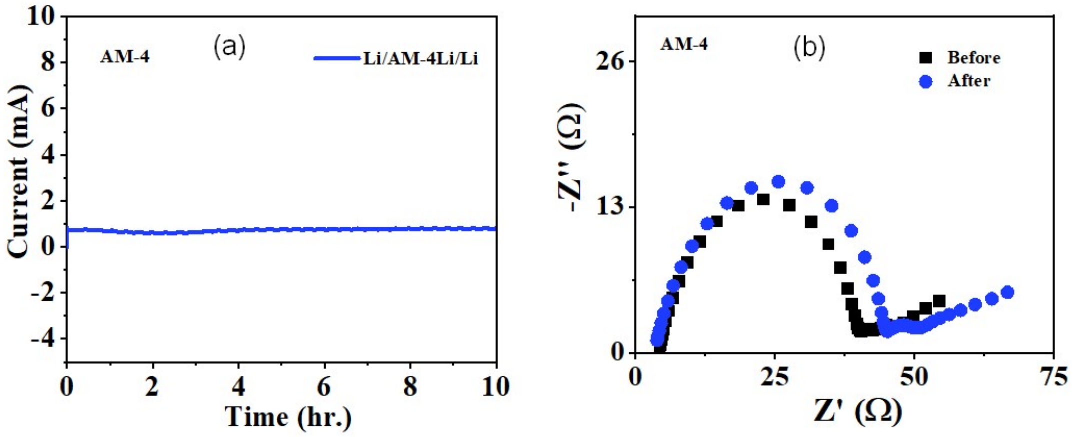

Figure S23: (a) The potentiostatic curve of Li/AM-4Li/Li. (b) The pre and post impedance curve of Li/AM-4Li/Li.

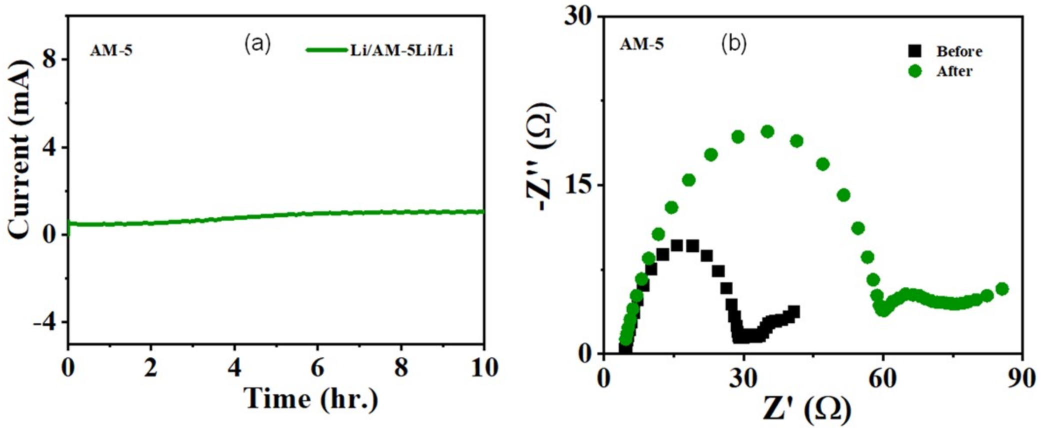

Figure S24: (a) The potentiostatic curve of Li/AM-5Li/Li. (b) The pre and post impedance curve of Li/AM-5Li/Li.

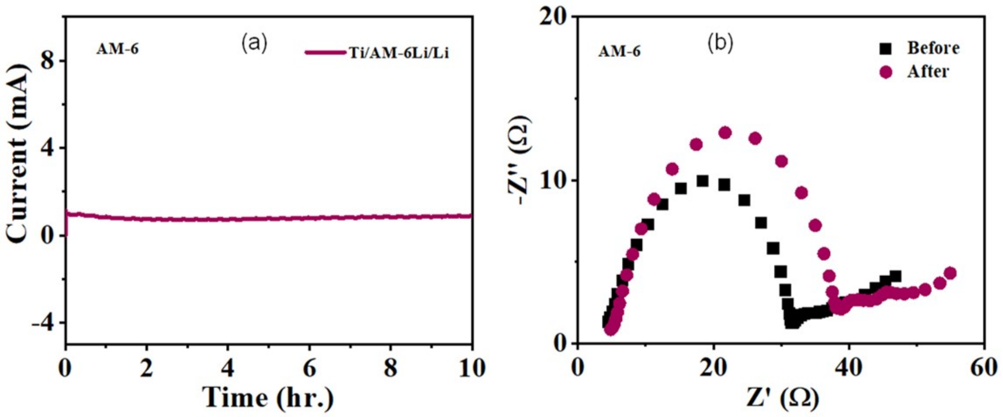

Figure S25: (a) The potentiostatic curve of Li/AM-5Li/Li. (b) The pre and post impedance curve of Li/AM-5Li/Li.

26. Electrochemical stability of SONs:

Chronoamperometry (CA) study was carried out using a Ti/AMLi/Li asymmetric cell to examine the electrochemical stability of the synthesised materials, and the corresponding potentiostatic spectra for AM-4, AM-5, and AM-6 are shown in Figures S26, S27, and S28, respectively. Here, in every instance, it demonstrates improved stability over a 10-hour period with little curve degradation. Under asymmetric cell setup, though the charge transfer process of Li/Li^+ is highly restricted, the stability of materials in potential-static condition is ensured and which is well accordance with the steep observed curves.[1b, 2]

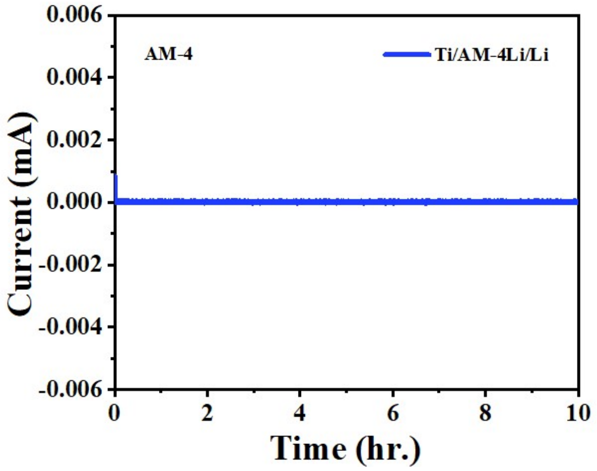

Figure S26: Potentiostatic curve of Ti/AM- 4Li/Li.

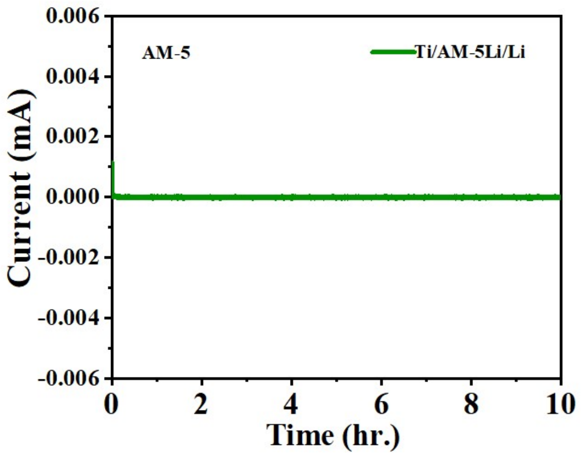

Figure S27: Potentiostatic curve of Ti/AM- 5Li/Li.

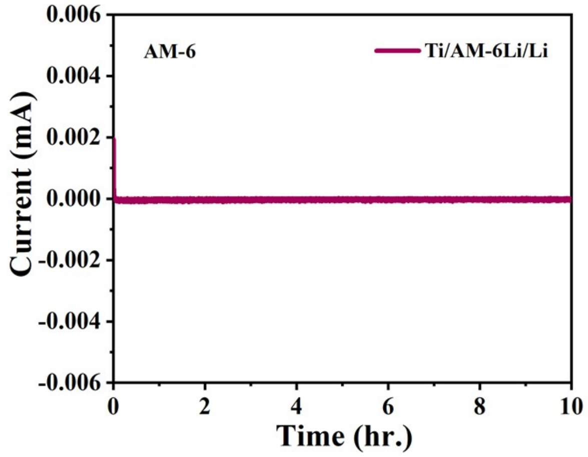

Figure S28: Potentiostatic curve of Ti/AM- 6Li/Li.

27. Computational details:

Periodic boundary condition calculations were carried out using the Vienna *Ab initio* Simulation Package (VASP) with a plane-wave basis set.[3,4] The X-ray diffraction (XRD) was simulated using the optimized geometries by the VESTA (Visualization for Electronic and STructural Analysis) software.[5] The computations employed the PBE level of theory, including Grimme's empirical dispersion correction.[6] An *ab initio* molecular dynamics (MD) simulation was performed in the isothermal–isobaric (NPT) ensemble until equilibrium was attained.[7,8] The MD simulation was run at 300 K using a Langevin thermostat and standard pressure, with a timestep of 1 fs.

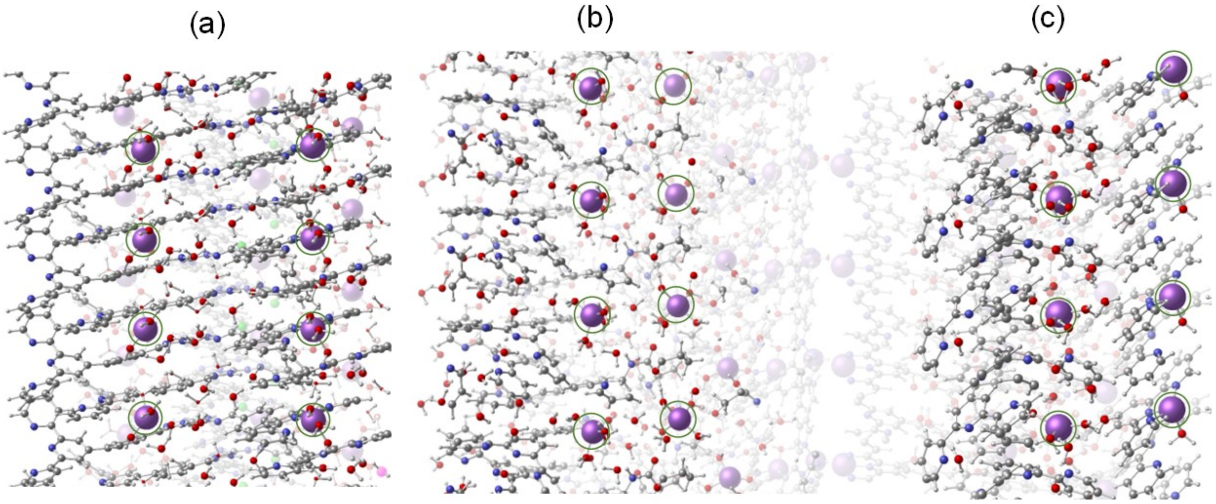

Figure S29: The snapshot is taken from MD simulation for SONs of (a) AM-4, (b) AM-5 and (c) AM-6 with Li-ion and water molecules at five ps. The Li-ion (in violet color) is shown in a ring for better visibility. The optimized geometries display the Li-ion migration along with amide functionality. [C: grey, N: blue, H: white, Li: violet, O: red].

28. SEM images of the composit films:

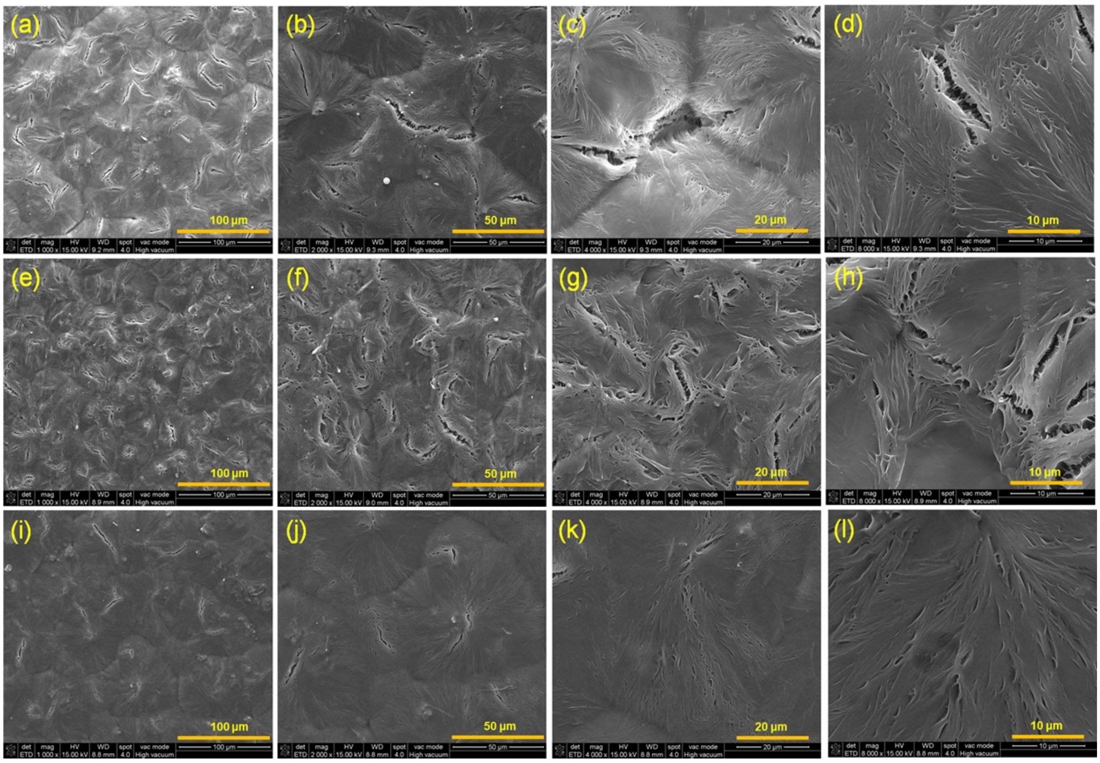

Figure S30. SEM images of the SONs imprinted PEO films for (a-d) AM-4, (e-h) AM-5, and (i-l) AM-6, respectively.

29. PXRD pattern of the composit films:

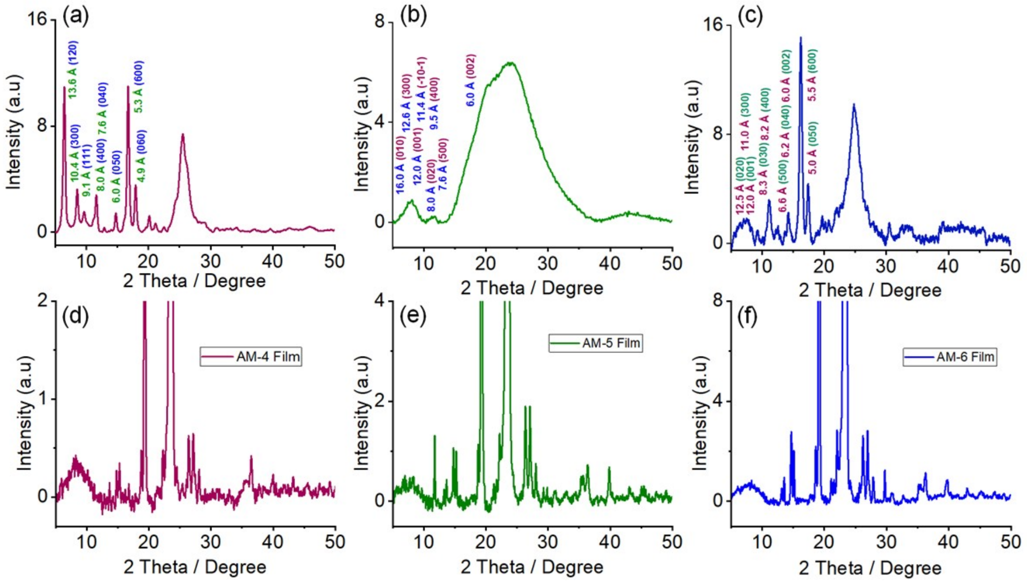

Figure S31. XRD profile of the SONs and SONs imprinted PEO films for (a) SONs of AM-4, (d) SONs imprinted PEO films of AM-4, (b) SONs of AM-5, (e) SONs imprinted PEO films of AM-5, and (c) SONs of AM-6, (f) SONs imprinted PEO films of AM-6, respectively.

30. X-ray refinements and sample preparation for PXRD measurements:

We have optimized all geometries in a periodic condition using VASP software and the theoretical X-ray diffraction (XRD) is simulated by the VESTA Crystallographic Software.

VESTA is an essential tool for visualizing and analysing refinement results. It offers an intuitive interface for generating theoretical powder X-ray diffraction (XRD) patterns directly from crystal structure data. This functionality is crucial for phase identification, structure verification, and understanding the relationship between a material's crystal structure and its diffraction pattern.

The software simulates theoretical X-ray diffraction patterns using a Cu $K\alpha_1$ radiation source (λ = 1.54 Å). VESTA works in conjunction with RIETAN-FP (F. Izumi and K. Momma, *Solid State Phenom.,* **2007**, *130*, 15.) to simulate both X-ray and neutron powder diffraction patterns based on the provided structural and lattice parameters.

Visualization of Simulated Powder Diffraction Patterns:

- ✓ **Integration with RIETAN-FP:**
  VESTA supports interaction with the RIETAN-FP Rietveld refinement package. It can automatically generate input files (*.ins) for RIETAN-FP based on the currently loaded crystal structure.
- ✓ **Displaying Simulated Patterns:**
  After executing RIETAN-FP in simulation mode (NMODE = 1), VESTA can read and display the resulting powder diffraction pattern. This allows direct visual comparison between simulated and experimental data.
- ✓ **Exporting for External Graphing:**
  VESTA can generate output files such as *.itx for use in plotting software like Igor Pro. These files typically contain intensity versus 2θ angle data, along with a list of calculated reflections, including their Miller indices (hkl), 2θ positions, d-spacings, and relative intensities.

Interpreting the Simulated Output:

- ✓ **Peak Positions:**
  The positions of peaks (in 2θ) correspond to the interplanar spacing's of the crystallographic planes, calculated via Bragg's Law: $\lambda = 2d \sin\theta$, where λ is the wavelength, d is the interplanar spacing, and θ is the Bragg angle. VESTA computes these d-spacing's from the unit cell parameters.
- ✓ **Peak Intensities:**
  Peak intensities are influenced by the types and arrangements of atoms in the unit cell. Heavier atoms with more electrons scatter X-rays more effectively. The calculated structure factor—based on atomic positions and scattering powers—determines the reflection intensities. VESTA also accounts for systematic absences due to lattice centering and space group symmetries.
- ✓ **Miller Indices (hkl):**
  Each peak corresponds to a specific set of crystallographic planes, represented by Miller indices (hkl). VESTA displays these indices for each reflection, aiding in the identification of which planes contribute to each observed peak.

Experimental powder X-ray diffraction (PXRD) pattern was recorded on sedimentation of the self-assembled morphology to acquire information about the molecular arrangements in SONs. To prepare the sample for that, first we prepare the self-assembled morphology of SONs. A solution of all three molecules (AM-4, AM-5, and AM-6) in DMF (2.5 mL, 5.0 mg/mL) was exposed to 2.5 mL of deionized water at 298 K (maintaining the same solvent ratio as we used

to achieve the self-assembled morphology). The dispersion was mixed thoroughly and left undisturbed to complete the automated self-assembly process. After 72 hr. sedimentation of the self-assembled morphology occur at the bottom of the vial. Then we remove the excess solvent. The residue was dried at room temperature under vacuum and submitted for PXRD measurements.

31. Reference:

[1] a) D. Wu, R. Liu, W. Pisula, X. Feng, K. Müllen, *Angew. Chem. Int. Ed.* **2011**, *50*, 2791-2794; b) A. Dey, V. R. Ramlal, S. S. Sankar, S. Kundu, A. K. Mandal, A. Das, *Chem. Sci.* **2021**, *12*, 13878-13887; c) M. Doyle, T. F. Fuller, J. Newman, *Electrochimica Acta* **1994**, *39*, 2073-2081.

[2] K. Jeong, S. Park, G. Y. Jung, S. H. Kim, Y.-H. Lee, S. K. Kwak, S.-Y. Lee, *J. Am. Chem. Soc.* **2019**, *141*, 5880-5885.

[3] G. Kresse and J. Furthmiiller, *Comput. Mater. Sci.*, **1996**, *6*, 15–50.

[4] G. Kresse and D. Joubert, *Phys. Rev. B.*, **1999**, *59*, 11–19.

[5] K. Momma and F. Izumi, *J. Appl. Crystallogr.*, **2011**, *44*, 1272-1276.

[6] S. Grimme, S. Ehrlich and L. Goerigk, *J. Comput. Chem.*, **2011**, *32*, 1456–1465.

[7] A. R. M. Parrinello, *Phys. Rev. Lett.*, **1980**, *45*, 1196.

[8] M. Parrinello and A. Rahman, *J. Appl. Phys.*, **1981**, *52*, 7182–7190.
